# Stimuli‐Responsive Afterglow from Luminescent Liquid Crystal Elastomers

**DOI:** 10.1002/adma.202516922

**Published:** 2025-11-20

**Authors:** Lansong Yue, Michael G. Debije, Albert P. H. J. Schenning

**Affiliations:** ^1^ Stimuli‐Responsive Functional Materials and Devices (SFD) Department of Chemical Engineering and Chemistry Eindhoven University of Technology (TU/e) Groene Loper 3 Eindhoven 5612AE The Netherlands; ^2^ Institute for Complex Molecular Systems (ICMS) Eindhoven University of Technology (TU/e) Groene Loper 3 Eindhoven 5612AE The Netherlands; ^3^ Interactive Polymer Materials (IPM) Eindhoven University of Technology (TU/e) Groene Loper 3 Eindhoven 5612AE The Netherlands

**Keywords:** active afterglow, luminescent actuator, polyurethane liquid crystal elastomer, stimuli responsive materials, ultralong lifetime

## Abstract

Stimuli‐responsive luminescent polymeric materials which combine afterglow emission with mechanical flexibility and adaptability to external stimuli are well‐suited for dynamic optical devices, encoding, anticounterfeiting, and soft robotics. However, current systems rely on static matrices and organic phosphors, which limit their afterglow duration, flexibility, processability, and responsiveness to various stimuli. Here, an active glow luminescent system for encoding, signaling, and tracking in dark environments by integrating inorganic ultralong afterglow phosphors with adaptive liquid crystal elastomer actuators is demonstrated. The resulting material exhibits ultralong emission (up to 1200 s) in the dark, enabling reprogrammable information encryption and reversible scattering‐to‐transparent transition upon mechanical, thermal, or light stimuli, allowing for multimodal decoding. A bicolor, spatially encoded phosphorescent soft actuator is fabricated that appears plain and unmarked under daylight, but reveals structured emission in the dark while simultaneously exhibiting shape change under heat or light. Incorporation of an additional fluorescent dye further endows the system with light‐environment sensitivity, enabling luminescent rolling wheels and fiber actuators capable of self‐signaling and recovery tracking. This work not only demonstrates constructing active luminescent materials but also provides valuable insights toward adaptive and interactive glowing optical devices.

## Graphical abstract

An active glow luminescent system for encoding, signaling, and tracking in dark environments has been developed by integrating inorganic ultralong afterglow phosphors with adaptive liquid crystal elastomer actuators.

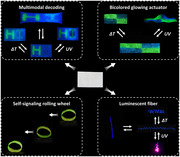

## 1 Introduction

Materials that autonomously glow in the dark without constant external excitation are being actively developed for uses requiring persistent optical signals. These materials, commonly known as phosphorescent, enable photon energy storage and delayed visible emission, with emerging applications in sensing,^[^ ^1^ ^]^ optical encryption,^[^ ^2^, ^3^ ^]^ anti‐counterfeiting,^[^ ^4^, ^5^, ^6^ ^]^ and night‐time display,^[^ ^7^ ^]^ among others. Phosphorescent materials are typically categorized into organic and inorganic phosphors. Among them, rare‐earth‐doped inorganic phosphors such as SrAl_2_O_4_:Eu^2+^, Dy^3+^ with green and Sr_2_MgSi_2_O_7_:Eu^2+^, Dy^3+^ with blue phosphorescence exhibit exceptional photo‐ and thermal‐stabilities and ultralong afterglows for minutes or even hours,^[^ ^8^, ^9^ ^]^ making them especially attractive in lighting,^[^ ^10^ ^]^ light or stress sensors,^[^ ^11^, ^12^, ^13^ ^]^ bioimaging,^[^ ^14^ ^]^ solar cells,^[^ ^15^ ^]^ and wearable fabrics.^[^ ^16^ ^]^

The growing demand for flexible, adaptive optical devices has recently intensified interest in phosphorescent polymeric materials that combine mechanical deformability with sustained emission. Current strategies primarily focus on molecular engineering,^[^ ^17^, ^18^, ^19^ ^]^ host–guest complexation,^[^ ^20^, ^21^ ^]^ H‐aggregation,^[^ ^22^ ^]^ crystallization,^[^ ^23^ ^]^ or self‐assembly,^[^ ^24^ ^]^ which often rely on rigid domains to stabilize the chromophores’ triplet states, leading to poor processability and flexibility.

Doping phosphors into polymers offers a straightforward alternative. This method typically involves embedding or dispersing phosphors into a polymeric host via polymerization or physical blending without chemical modification.^[^ ^25^, ^26^, ^27^, ^28^ ^]^ For example, ionic phosphorescent polyacrylamide and polyvinyl alcohol were incorporated into polydimethylsiloxane, preparing elastomers with robust phosphorescent performance even under repeated mechanical deformations.^[^ ^29^ ^]^ Another work doped triphenylene‐based aromatic secondary amines into polyurethanes (PUs), producing a phosphorescent film with reversible mechano‐response.^[^ ^30^ ^]^ Other polymer matrices, including poly(methyl methacrylate),^[^ ^31^, ^32^ ^]^ epoxy polymers,^[^ ^33^, ^34^ ^]^ styrene‐ethylene‐butylene‐styrene,^[^ ^35^ ^]^ and polyacrylamide,^[^ ^36^ ^]^ have also been explored. However, most existing polymer hosts are passive, unable to actively respond to external stimuli. Rather, they must be mechanically manipulated to deform. These systems typically incorporate organic phosphors with relatively shorter lifetimes, lower emission intensity, with a fixed, single color emission output. These challenges underscore the need for an active system that combines persistent phosphorescence with the active adaptability of responsive soft polymer materials.

Liquid crystal elastomers (LCEs) are soft adaptive materials combining the anisotropy of liquid crystals with the elasticity of rubbers, capable of large and reversible shape deformations, and switchable transparency under external triggers.^[^ ^37^, ^38^, ^39^, ^40^ ^]^ These triggers disrupt the liquid crystalline order, resulting in anisotropic contraction or expansion,^[^ ^41^, ^42^, ^43^, ^44^ ^]^ making them particularly attractive as soft robotics,^[^ ^45^ ^]^ wearable devices,^[^ ^46^, ^47^ ^]^ or haptic devices.^[^ ^48^, ^49^ ^]^ However, most LCE studies have focused on their shape‐changing behaviors, with limited exploration into optical functionalities. In particular, to the best of our knowledge, luminescent LCEs with significant afterglow have not yet been reported.^[^ ^2^, ^50^, ^51^ ^]^ Integrating phosphorescent properties into LCEs would not only enable energy storage and release through afterglow but also allow actuation to be visualized and tracked in real‐time, or even lighting the surrounding environment in the dark. Furthermore, it opens the door to bridge sensing and signaling, turning actuation into communication, mimicking nature's use of light, where organisms such as fireflies or deep‐sea cephalopods use bioluminescent pulses to attract, evade, or communicate.^[^ ^52^ ^]^ Such a combination may enable new generations of soft robotics or interfaces that not just sense and act, but signal and “speak.”

Herein, we present an active luminescent system that combines the phosphorescence of inorganic phosphors with the adaptability of liquid crystal elastomers. SrAl_2_O_4_:Eu^2+^, Dy^3+^ and Sr_2_MgSi_2_O_7_:Eu^2+^, Dy^3+^ were selected for providing ultralong afterglow, while a thermoplastic LCE with low activation temperature was prepared to enable actuation, optical modulation, and processability. The resulting composite can be shaped into scattering films that exhibit rewritable phosphorescent encryption either via maskless photopatterning or simple handwriting. These films enable scattering‐to‐transparent transitions under three different stimuli, allowing for information encoding and multimodal decoding, setting them apart from conventional polymer systems with fixed brightness. We further demonstrate an encoded bicolored phosphorescent actuator, featuring regions visually indistinguishable under daylight that reveal distinct multicolor phosphorescence and shape changes when activated in the dark. Moreover, fluorescent dye incorporation extends the optical modulation, allowing the development of luminescent rolling wheels and fiber actuators with self‐tracing functionalities. These actuators can dynamically adjust their emission color in response to ambient light, enabling real‐time tracking and signaling in dark environments.

## 2 Results and Discussion

**Figure 1:**
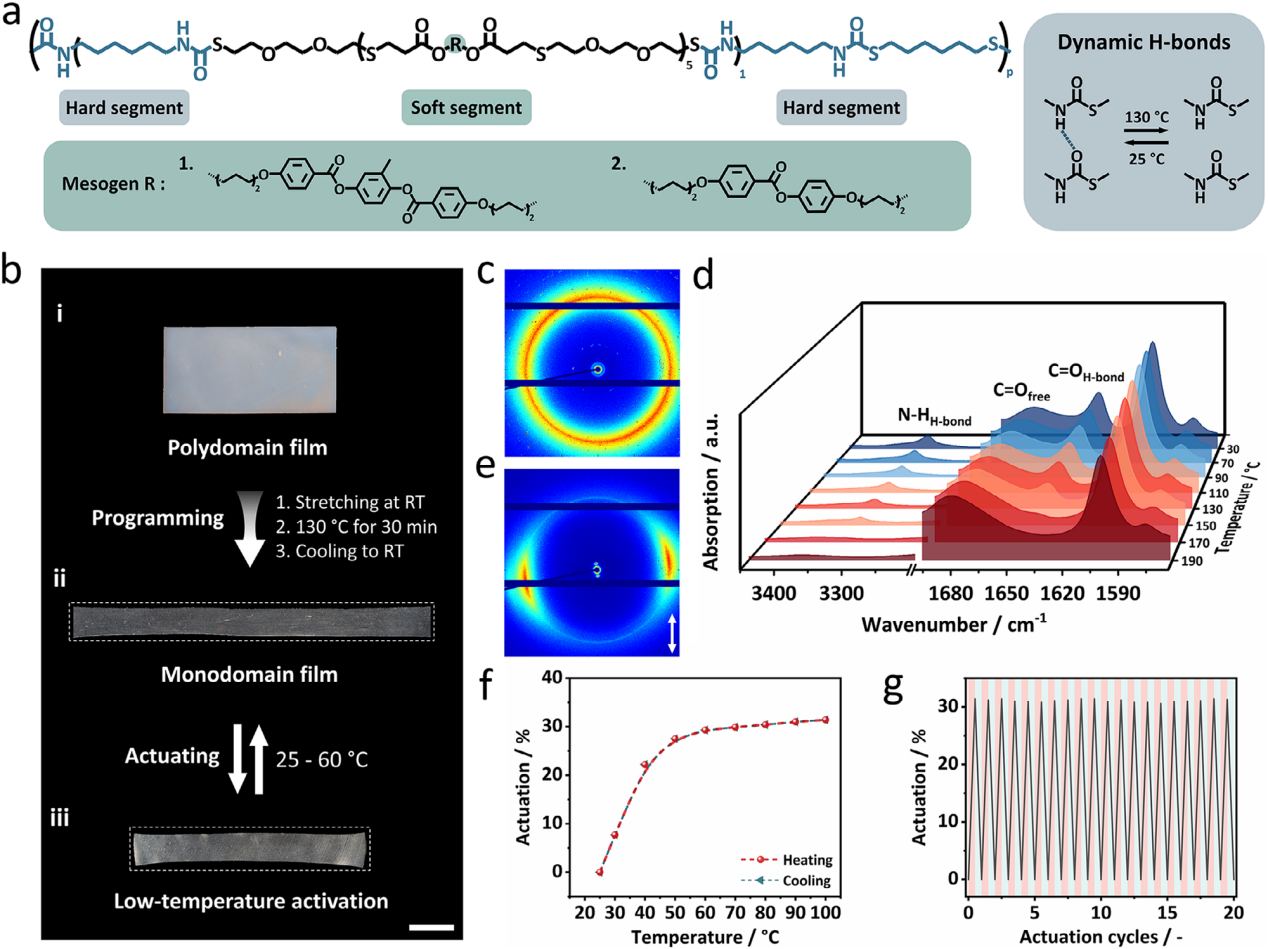
a) Schematic illustration of the chemical structure of the PULCE. The liquid crystal monomers used are shown at the bottom (green background). The dynamic hydrogen bonding is highlighted on the right (gray background). b) Photographs of (i) the polydomain PULCE film, (ii) the monodomain film after uniaxial stretching, and (iii) the deformed film after heating to 60 °C, showing contraction along the alignment direction and expansion perpendicular to it (scale bar = 1 cm). c) 2D‐WAXS pattern of the polydomain PULCE film, showing an isotropic diffraction ring. d) Temperature‐dependent FT‐IR spectra of the PULCE from 30 to 190 °C, highlighting changes in the hydrogen‐bonded N─H and C═O stretching bands upon heating. e) 2D‐WAXS pattern of the monodomain PULCE film, exhibiting strong diffraction anisotropy and confirming molecular alignment. f) Thermal actuation of the aligned film between 25 and 100 °C, showing reversible dimensional changes. g) Actuation stability over 20 heating‐cooling cycles, demonstrating consistent and repeatable shape transformation without performance loss. Rose color denotes heating and light blue regions represent cooling periods.

To develop stimuli‐responsive luminescent polymers, a new hydrogen‐bonded thermoplastic polyurethane‐based liquid crystal elastomer (PULCE) with 90 wt% liquid crystalline “soft” segments and 10 wt% thio‐urethane “hard” domains was synthesized (**Figure** **1**a).^[^ ^53^ ^]^ For the soft segment, acrylate mesogens containing two and three benzene units were chosen to enable actuation around body temperature.^[^ ^54^, ^55^ ^]^ The synthesis includes a sequential thiol‐acrylate and thiol‐isocyanate addition polymerization (Figure S1, Supporting Information). Initially, diacrylate mesogens containing two and three benzene units were reacted with a dithiol to form thiol‐terminated oligomers, which were subsequently capped with a diisocyanate and extended via an additional dithiol derivative to yield the final PULCE. The successful formation of the desired polymer was confirmed via ^1^H‐NMR spectroscopy (Figure S2, Supporting Information). Gel permeation chromatography (GPC) analysis revealed a high number‐average molecular weight (*M*_n_) of ≈59 kg mol^−1^ with a relatively low polydispersity index (PDI) of 1.9 (Figure S3, Supporting Information). The presence of hydrogen bonding was investigated by Fourier‐transform infrared (FT‐IR) spectroscopy (Figure S4, Supporting Information), which displayed characteristic hydrogen‐bonded amine stretching bands (N─H_H‐bond_) at 3315 cm^−1^ and carbonyl stretching bands (C═O_H‐bond_) at 1640 cm^−1^. Thermal characterization via differential scanning calorimetry (DSC) demonstrated a glass transition (*T*_g_) at −29 °C and a melting point (*T*_m_) of 170 °C (Figure S5a, Supporting Information). Thermogravimetric analysis (TGA) indicated thermal decomposition onset (*T*_d_) at ≈260 °C, ensuring processability (Figure S5b, Supporting Information). Polarized optical microscopy (POM) revealed a birefringent texture at room temperature which gradually disappeared upon heating and reappeared upon cooling, confirming a nematic‐isotropic transition (*T*_ni_) at ≈60 °C (Figure S6, Supporting Information). Notably, compared with our previously reported PULCE which exhibited *T*_m_ of ≈175 °C and *T*_ni_ of ≈80 °C,^[^ ^53^ ^]^ the new polymer showed a markedly lower *T*_ni_ while maintaining comparable melting behavior, enabling actuation at much lower operating temperatures.

A free‐standing polydomain PULCE film was prepared via hot pressing at 170 °C (Figure 1b‐i). The resulting film exhibited white opacity and displayed uniform isotropic ring patterns in 2D wide‐angle X‐ray scattering (2D‐WAXS) (Figure 1c), consistent with an unoriented mesogen distribution. Tensile testing indicates an elongation up to 2500%, underscoring the robust elasticity of the polymer film (Figure S7, Supporting Information). The thermal reversibility of hydrogen bonds was tested by temperature‐dependent FT‐IR measurements (Figure 1d). As temperature increases above 110 °C, the integrated area of both hydrogen‐bonded amine and carbonyl stretching bond peaks exhibited a sudden decrease. A shoulder band near 1680 cm^−1^, corresponding to free carbonyl groups, became increasingly prominent. Disappearance of the hydrogen‐bonded absorption occurred near 190 °C, indicating full dissociation of the dynamic linkages. Notably, upon subsequent cooling, the hydrogen‐bonded amine and carbonyl peaks reappeared, demonstrating the recovery of supramolecular interactions (Figure S8, Supporting Information). Dynamic mechanical thermal analysis (DMTA) reveals a significant modulus drop above 120 °C (Figure S9, Supporting Information), consistent with the dissociation of physical cross‐links. Based on these observations, an aligned PULCE sample was made from the polydomain film. The film was stretched 200% uniaxially at room temperature, followed by annealing at 130 °C for 30 min, enabling the rearrangement of molecular alignment upon cooling, resulting in an aligned monodomain film (Figure 1b‐ii). To verify the molecular alignment, 2D‐WAXS characterization was conducted which exhibited a pronounced diffraction signal perpendicular to the stretching direction (Figure 1e). The birefringence of the aligned film under crossed polarizers further corroborated this alignment (Figure S10, Supporting Information). Thermo‐actuation studies of the aligned PULCE film demonstrated a fully reversible actuation strain of ≈30% across the *T*_ni_ (≈60 °C) with contraction along the director field and expansion perpendicular to it (Figure 1b‐iii,f; Video S1, Supporting Information), maintaining excellent cycling stability over at least 20 cycles (Figure 1g). This macroscopic deformation originates from the thermally induced order‐disorder (nematic‐isotropic) transition of the liquid crystal segments.^[^ ^53^ ^]^ Importantly, in contrast to our earlier PULCE films which required heating above 80 °C to reach maximum actuation,^[^ ^53^ ^]^ the present system responds at significantly lower temperatures, reaching ≈30% strain already at ≈60 °C, and even near body temperature, it still deforms by ≈20%. Such a combination of mechanical robustness, reversible hydrogen bonding networks, and low‐temperature activation demonstrates that the PULCE is well‐suited for constructing stimuli‐responsive phosphorescent actuators.

**Figure 2:**
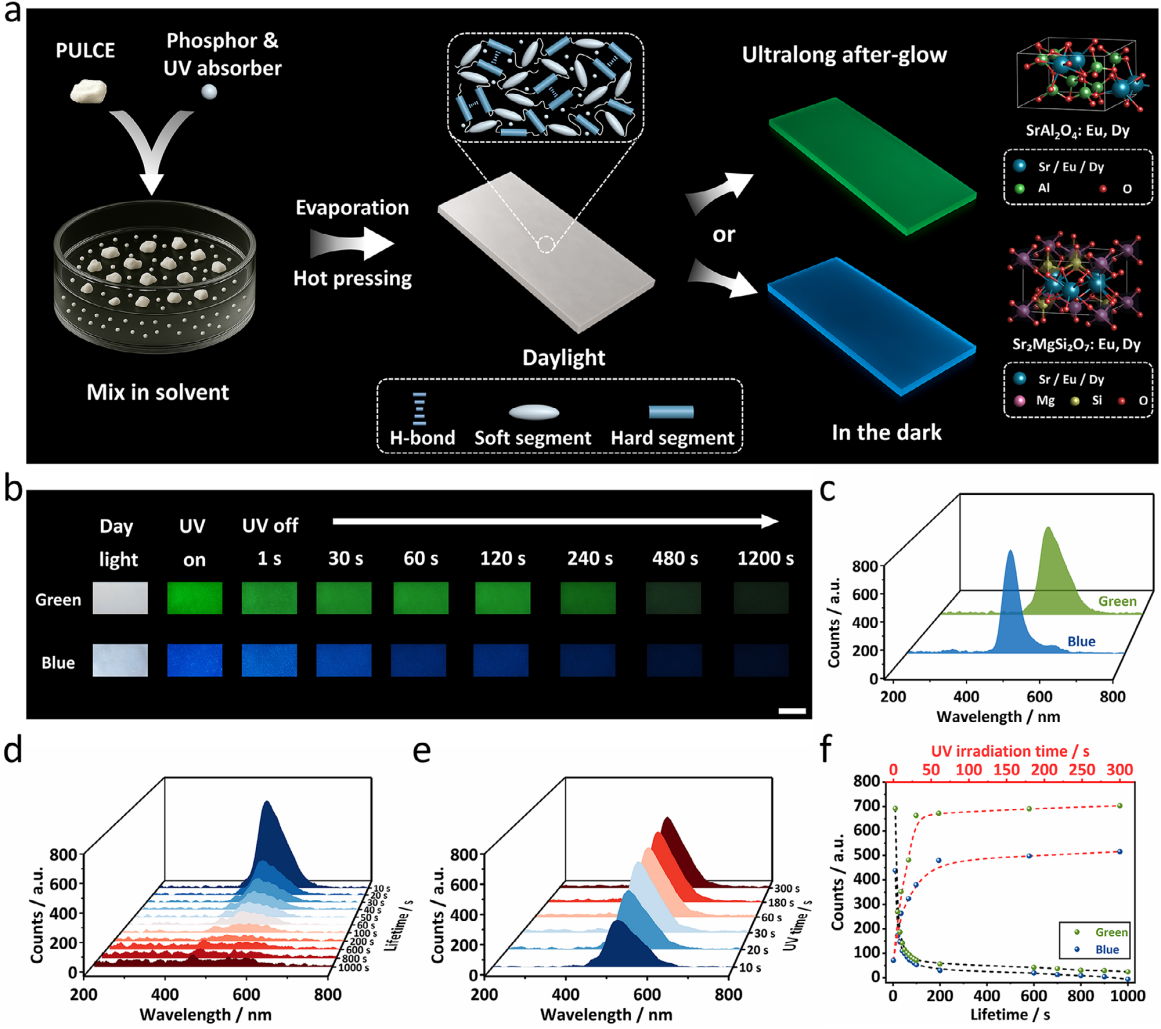
a) Schematic depicting the fabrication of the phosphorescent PULCE films. The crystal structures of the two phosphors are shown on the right. b) Photographs of PULCE‐G and PULCE‐B films under daylight, under UV excitation (365 nm, 20 mW cm^−2^), and at various afterglow times after UV illumination (365 nm, 20 mW cm^−2^ for 30 s) (scale bar = 1 cm). c) Normalized phosphorescence spectra of PULCE‐G and PULCE‐B films. d) Normalized delayed phosphorescence spectra of PULCE‐G film after UV irradiation. e) Normalized phosphorescence spectra of PULCE‐G film measured immediately after different UV exposure durations. f) Normalized phosphorescence intensity versus lifetime (bottom axis) and UV irradiation time (top axis) for PULCE‐G and PULCE‐B films.

To prepare stimuli‐responsive phosphorescent PULCEs, two commercially available ultralong after‐glow inorganic phosphors with green and blue emission colors were used. The green‐emitting phosphor (G), primarily composed of SrAl_2_O_4_:Eu^2+^, Dy^3+^ with a monoclinic structure, exhibits a broad after‐glow emission peak at ≈500 nm,^[^ ^56^, ^57^ ^]^ while the blue‐emitting phosphor (B), Sr_2_MgSi_2_O_7_:Eu^2+^, Dy^3+^ with a tetragonal structure emits at ≈470 nm (**Figure** **2**a, right).^[^ ^58^, ^59^, ^60^ ^]^ Upon photoexcitation, Eu^2+^ ions absorb photon energy and are promoted to an excited state, generating electron–hole pairs. Some of these charge carriers are captured by adjacent Dy^3+^ ions acting as trapping centers.^[^ ^13^, ^61^ ^]^ Upon removing the illumination, the trapped electrons are thermally released over time, recombining with Eu^2+^ ions and returning the electrons to the ground state. This decay leads to the delayed emission of photons, observed as ultralong afterglow. Both phosphors appear white under daylight. Scanning electron microscopy (SEM) showed both phosphors possess irregular polyhedral morphologies with average sizes of ≈8 ± 2 µm (G) and ≈15 ± 5 µm (B), respectively (Figure S11, Supporting Information). Phosphorescence lifetime measurements revealed that the green‐emitting phosphor exhibited a peak luminous intensity of ≈11 150 mcd m^−2^ with emission visible for over 12 h in the dark (Figure S12a, Supporting Information). In comparison, the blue‐emitting phosphor showed a lower maximum afterglow intensity (≈1879 mcd m^−2^) and a more rapid decay (Figure S12b, Supporting Information). Images for both phosphors were captured under daylight, during ultraviolet (UV) irradiation, and after UV‐off, confirming the blue and green phosphorescence in the dark (Figure S13, Supporting Information). Importantly, both phosphors can be excited by daylight, but UV exposure allows for more efficient photon harvesting, so a UV light (365 nm, 20 mW cm^−2^) was used as an excitation source for all subsequent experiments.

To incorporate the phosphors into the PULCE matrix, a solvent‐casting and hot‐pressing process was used (Figure 2a). A mixture of a phosphor (2 wt%) and a UV absorber (1 wt%) was dispersed in a PULCE solution, followed by solvent evaporation and hot‐pressing the resulting material into uniform, freestanding films. Incorporating the UV photothermal dye enables efficient conversion of UV irradiation to heat, thereby endowing the films with light‐responsive capability. Notably, the polydomain PULCE‐G (green phosphor) and PULCE‐B (blue phosphor) films appeared as white as the undoped PULCE film under daylight, making them visually indistinguishable (Figure 2b). Optical microscopy images confirmed the homogeneous distribution of phosphor particles within the films (Figure S14, Supporting Information): both PULCE‐G and PULCE‐B films exhibited well‐dispersed particles without apparent clustering or aggregation in the observed regions, consistent with the spatially uniform emission observed in Figure 2b. DSC analysis of two doped films revealed that both *T*_g_ (−29 °C) and *T*_m_ (170 °C) remained unchanged (Figure S15a,b, Supporting Information), and TGA confirmed *T*_d_ up to 250 °C (Figure S15c,d, Supporting Information). POM measurements also showed the birefringence textures at room temperature and an unaltered *T*_ni_ at 60 °C (Figure S16, Supporting Information). In addition, no major difference in moduli of the doped films was observed. The modulus decreases associated with hydrogen bonding dissociation above 120 °C indicated the good compatibility between the phosphors and PULCE matrix (Figure S17, Supporting Information). UV–vis absorption spectra of the two doped PULCE films demonstrated absorption throughout the visible spectrum, with a distinct peak near 348 nm attributed to the UV absorber (Figure S18, Supporting Information). To characterize the phosphorescence, the doped films were first kept in darkness 12 h to eliminate residual phosphorescence. Upon UV exposure (20 mW cm^−2^ for 30 s), PULCE‐G and PULCE‐B films exhibited green and blue emissions, respectively (Figure S19, Supporting Information). Upon removing the light source, both films showed bright emission and remained visible even after 20 min in the dark (Figure 2b). The corresponding phosphorescence spectra showed obvious emission peaks at 500 nm for PULCE‐G and 470 nm for PULCE‐B films (Figure 2c). Afterglow lifetime analysis revealed that the PULCE‐G film underwent a sharp decrease in emission intensity within the first 10 s (Figure 2d), followed by a gradual decay across several minutes, consistent with the green phosphor's high initial emission brightness and decay trend. PULCE‐B film revealed similar behavior but with lower emission intensity and detectable phosphorescence for up to 1000 s (Figure S20a, Supporting Information). Additionally, to quantify the UV irradiating efficiency, we exposed the films to UV light for varying durations (10–300 s) and recorded the initial emission intensity. Both doped films showed rapid saturation after 60 s UV exposure (Figure 2e; Figure S20b, Supporting Information). To validate the stability of the phosphorescent performance after doping, phosphorescence decay curves were plotted for both pristine phosphors and corresponding doped films under identical excitation and measurement conditions (Figure 2f; Figure S21, Supporting Information). Both PULCE‐G and PULCE‐B films maintained the ultralong afterglow behavior of the respective phosphors with emission lifetimes exceeding 1000 s, indicating the compatibility and stability between the PULCE matrix and the phosphors.

**Figure 3:**
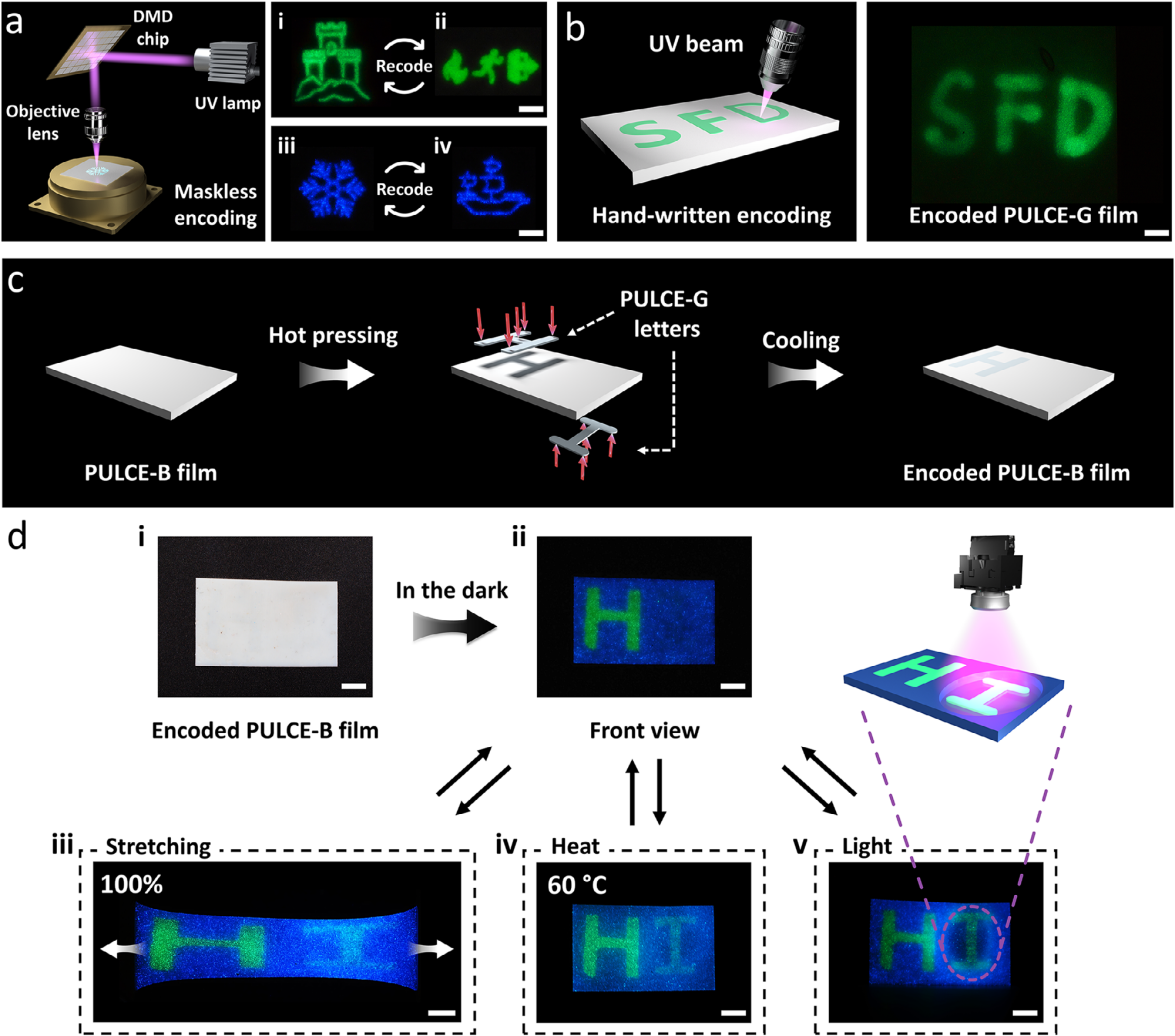
a) Maskless encoding of polydomain phosphorescent PULCE films using a digital micromirror device (DMD) exposure system. Complex patterns such as (i) a castle is written onto a PULCE‐G film and subsequently erased and (ii) reprogrammed with a different design. Similar reversible patterning is demonstrated on PULCE‐B film (iii, iv) (scale bars = 5 mm). b) Direct hand‐written encoding on a PULCE‐G film using a concentrated UV beam as a “pen.” Handwritten “SFD” characters are shown on the right (scale bar = 5 mm). c) Schematic illustrating an encoded phosphorescent PULCE composite system by hot pressing: a PULCE‐B base film is hot pressed with PULCE‐G “H” and “I” segments embedded on opposite sides. d) Photographs of (i) the encoded film, appearing homogeneous under daylight, while (ii) showing glowing “H” in the dark from the front view. Demonstration of three activation strategies for decoding the hidden “I” character: (iii) mechanical stretching induced decoding (100% elongation), (iv) thermally induced decoding (above *T*_ni_), and (v) photothermally induced decoding (localized UV illumination) (scale bars = 5 mm). The violet dashed circles indicate the regions locally irradiated with UV light.

Phosphorescent PULCE films with erasable and rewritable optical pictures can be prepared using a facile photopatterning method. Pattern writing was achieved by irradiating the film surface with a digital micromirror device (DMD)‐based projection system, which eliminates the need for physical masks and enables the high‐resolution inscription of arbitrary images directly through projected UV light (**Figure** **3**a). Upon UV exposure, localized excitation of the embedded phosphors results in afterglow patterns, such as a castle, with high spatial clarity (Figure 3a‐i). The patterns can be thermally erased: gentle heating accelerates the release of trapped electrons, which enhances the recombination rate at Eu^2+^ centers. This leads to rapid quenching of the emission and effective optical “resetting” of the film surface. A new pattern can then be re‐written by subsequent UV exposure (Figure 3a‐ii), confirming the doped film's full re‐writability and emission over multiple cycles (Figure S22a, Supporting Information). A similar reversible encoding capability was also observed in PULCE‐B film, as shown in Figure 3a‐iii and iv. The afterglow patterns remain high‐resolution and thermally assisted erasure again allowed for repeatable re‐inscription with no difference in phosphorescence intensity (Figure S22b, Supporting Information). Additionally, the films also allowed for spontaneous, hand‐written inscription (Figure 3b; Video S2, Supporting Information). By using a concentrated UV light beam as a “pen”, the letters “SFD” were hand‐written onto a PULCE‐G film in the dark, where the glowing writing path can be easily tracked in real time by the human eye. A quick exposure to elevated temperature can also be used to erase letters. Once the glow disappears, the film returns to its pre‐written state, ready for the next message. To evaluate the durability of this rewriting process, 100 write‐erase cycles on both PULCE‐G and PULCE‐B films were performed. Phosphorescence spectra were recorded every 10 cycles under identical excitation and measurement conditions (Figure S23, Supporting Information). The emission profiles remained consistent in shape and peak position throughout the entire cycling process. Additionally, the peak emission intensities showed negligible fluctuation across 100 cycles, confirming that the phosphorescent performance remains stable under repeated write‐erase usage, which is essential for practical long‐term applications.

The afterglow appearance of PULCE films can be triggered by three independent stimuli: mechanical stretching, temperature, and light. In the relaxed state, the polydomain white colored films (thickness: 700 µm) strongly scatter light and conceal any underlying information, making them unreadable under daylight or even during afterglow in the dark. Under uniaxial stretching (up to 100% strain) in the dark, the thickness of the film decreases, and the polymer segments align in the stretching direction, leading to optical transparency, allowing the viewer to visually read the hidden “SFD” text under the film's own emission without external lighting (Figures S24 and S25, Supporting Information). When the strain was released, the film returned to its original scattering state, re‐concealing the background information. No loss in emissive intensity was observed over repeated stretching‐releasing cycles, confirming the durability of both the phosphors and the PULCE matrix. Thermal activation offers a second route to switch on the afterglow appearance of the PULCE films: upon heating above *T*_ni_, the initially opaque film undergoes a nematic‐isotropic phase transition, enabling the modulation of the visual appearance of the film's afterglow in the dark (Figures S26, Supporting Information). Importantly, a new phenomenon was observed when a dimly glowing film in its scattering state was suddenly placed on a hot stage at 80 °C. As shown in Figure S27 and Video S3 (Supporting Information), an immediate and intense flash of phosphorescence followed by a gradual decay was observed. This flash may be attributed to the stimulated release of trapped charge carriers. Specifically, the sudden high temperature promotes the activation of deeper trap states, enabling electrons to escape and recombine with enhanced efficiency. Incorporation of a UV absorber enables a third optically driven strategy for dynamic glow appearance. Upon UV irradiation (150 mW cm^−2^ for 30 s), both phosphorescent films reached a temperature exceeding 70 °C due to the photothermal conversion, well above the *T*_ni_ threshold (Figure S28, Supporting Information). This light‐induced thermal activation triggers the same phase transition mechanism as direct heating. By spatially controlling UV light illumination, localized optical switching was also demonstrated (Figure S29, Supporting Information).

Based on these three strategies for optical switching, a multicolor polymer film was constructed that can store and reveal encoded information (Figure 3c). Two PULCE‐G films shaped like the green phosphorescent letters “H” and “I” were placed on a blue phosphorescent PULCE‐B film (1.5 × 2.5 cm^2^, 700 µm). The “H” was placed on the front‐left side, while the “I” was placed on the back‐right side of the PULCE‐B film. By hot pressing, a uniform, flat white colored film was obtained that gave no visible protrusions or embedded patterns under daylight (Figure 3d‐i). When exposed to UV light in darkness, the front information “H” became visible, glowing green against the emissive blue background, while the information encoded on the back (“I”) remained completely hidden (Figure 3d‐ii) because of scattering of the film. The hidden “I”, however, can be decoded in the dark using the three different stimuli described above. 1) Stretching decoding: upon uniaxial stretching, the film became thinner and aligned to the stretching direction, reducing scattering. The encoded “I” gradually appeared, especially at 100% strain, while the “H” remained undisturbed (Figure 3d‐iii; Figure S30a, Supporting Information). Owing to the intrinsic elasticity of PULCE matrix, this mechanochromic process is fully reversible. 2) Heat decoding: heating the entire film above *T*_ni_ induces a transparency change, enabling the simultaneous visualization of both the front “H” and formerly hidden “I” (Figure 3d‐iv; Figure S30b, Supporting Information). 3) Light decoding: by locally irradiating the region over the encoded “I” with UV light, the exposed area was photoheated above *T*_ni_, offering a contactless way for decoding the information (Figure 3d‐v; Figure S30c, Supporting Information). These dynamic glow materials demonstrate their potential for dynamic anti‐counterfeiting applications.

To prepare a phosphorescent soft actuator that can be monitored in the dark, a uniaxially aligned PULCE‐G sample was prepared by stretching the doped films to 200% strain and annealing at 130 °C for 30 min. Upon cooling to room temperature, the film exhibited a transparent appearance under daylight, as shown in Figure S31a (Supporting Information). To assess its optical transparency, UV–vis transmission spectroscopy was conducted. The PULCE‐G film showed a high transmittance of ≈90% across the visible range (Figure S31b, Supporting Information), indicating low optical scattering. Additionally, when placed over a patterned background, the PULCE‐G film allowed clear visibility of the underlying features, further confirming its optical transparency and minimal optical interference from the embedded phosphors (Figure S31c, Supporting Information). The molecular alignment was verified by the pronounced diffraction pattern orthogonal to the alignment direction in 2D‐WAXS profiles (Figure S31d, Supporting Information) and the obvious optical changes in the appearance of the birefringent material under POM with crossed polarizers (Figure S31e, Supporting Information). In the dark, the actuator showed an ultralong glowing effect similar to the unaligned film. Remarkably, the glowing actuator exhibited reversible contraction with increasing temperature while continuously emitting green afterglow (Figure S31f and Video S4, Supporting Information), allowing the real‐time actuation to be visualized in the dark. The actuator contracts up to 30% from room temperature to 60 °C and maintains the response over at least 20 thermal cycles without degradation (Figure S31g,h, Supporting Information). Similar thermal actuation behavior (≈30%) was observed in the PULCE‐B actuator, while emitting a blue afterglow in the dark (Figure S32 and Video S4, Supporting Information). These experiments indicate that the incorporation of phosphors does not affect the dynamic shape change behavior of the PULCE matrix. Additionally, the phosphorescent performance of both PULCE‐G and PULCE‐B actuators remained stable after 100 thermal actuation cycles, with no detectable shift in emission spectra, consistent peak intensities (>98% retention ratio), and nearly unchanged decay curves, further confirming the stability of the dynamic afterglow (Figure S33, Supporting Information). In addition to thermal actuation, the incorporation of the UV absorber enables light responsive glowing actuators. Under focused UV irradiation, the films exhibited localized contraction due to photothermal heating and recovered to the original shape once the UV source was removed, as demonstrated in Figure S34 and Video S5 (Supporting Information). These observations illustrate the aligned phosphorescent PULCE films are not only controllable by dual stimuli but also visually traceable in the dark, exemplifying the great potential as multimodal, self‐luminescent actuators. Moreover, both phosphorescent actuators demonstrated excellent environmental robustness. Actuators stored under ambient conditions for over one year maintained both consistent phosphorescence performance and thermal actuation behavior, exhibiting long‐term stability in air (Figure S35, Supporting Information). In addition, immersion tests in water for 12, 24, 48, 72, and 96 h revealed no significant loss in emission brightness, indicating strong tolerance to moisture exposure (Figure S36, Supporting Information). Such results suggest that the phosphorescent PULCE system remains functional under realistic environmental conditions, further supporting its suitability and potential for practical use in real‐world applications, including outdoor devices.

**Figure 4:**
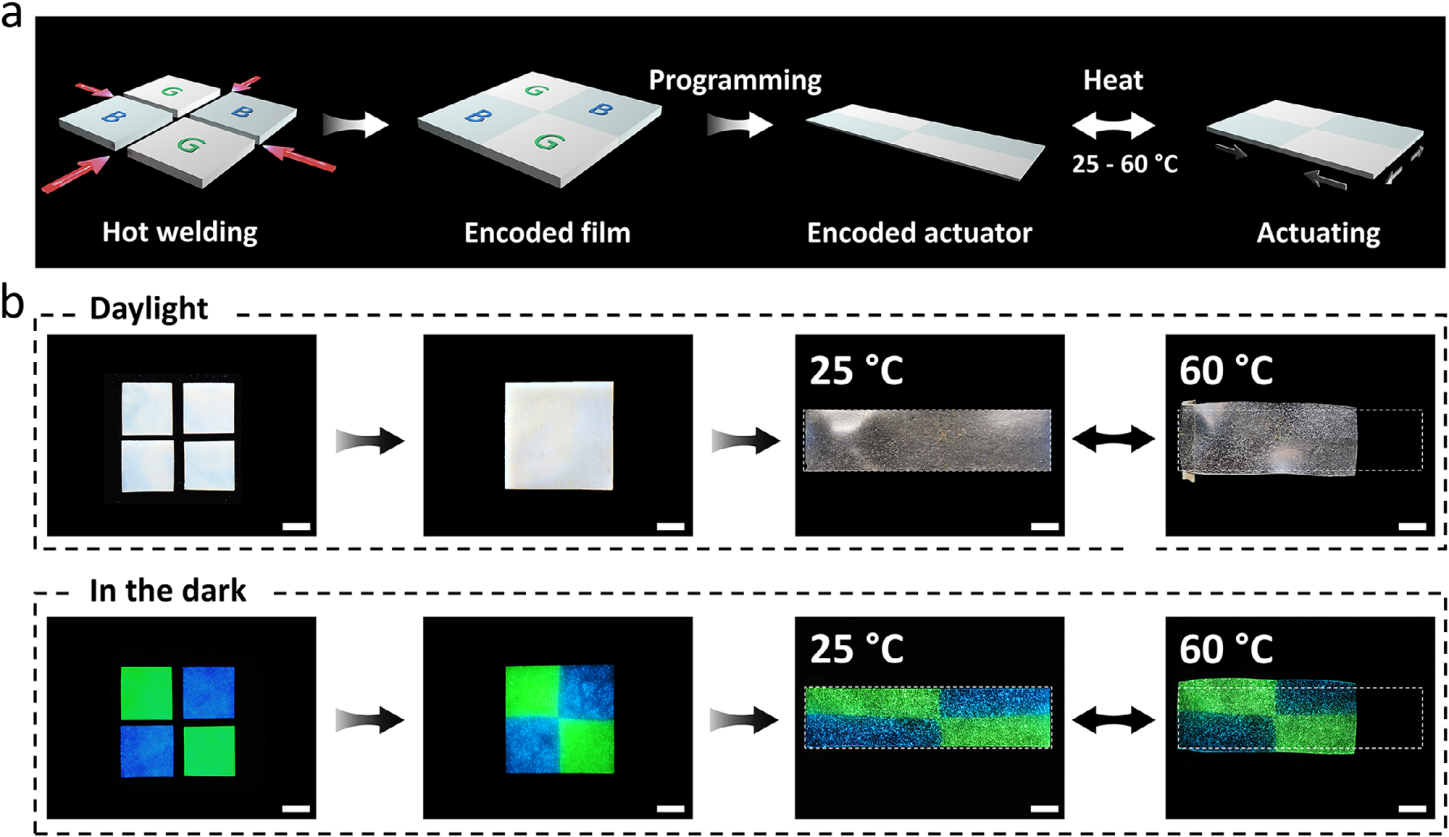
a) Schematic depicting the fabrication of a hot‐welded encoded bicolored phosphorescent actuator: two square PULCE‐G films and two square PULCE‐B films are orthogonally arranged and fused under hot pressing. The resulting film exhibits a seamless interface with no visible junctions under daylight. The welded film can subsequently be uniaxially programmed, enabling thermal response. b) Photographs under daylight and in the dark corresponding to each stage of fabrication, programming and thermal actuation of the hot‐welded encoded phosphorescent actuator (scale bars = 5 mm).

Next, we prepared spatially programmable, encoded, bicolored phosphorescent actuators. Four individual square films (two PULCE‐G films and two PULCE‐B films) were first prepared and arranged in a cross‐shaped layout (**Figure** **4**a). Using hot welding, these units were seamlessly fused into one film with no welding lines under daylight. Subsequent uniaxial stretching (200%) followed by thermal annealing at 130 °C yielded a uniformly oriented film, as confirmed by 2D‐WAXS patterns and POM images (Figure S37a–c, Supporting Information). The soft actuator maintained 30% reversible deformation for temperature cycling between 25 °C and 60 °C and also responding rapidly to local UV exposure (Figure S37d–g, Supporting Information). Importantly, in the dark, the embedded spatial coding became visible through crossed green and blue afterglow regions, showcasing dynamic multicolor phosphorescence (Figure 4b; Video S6, Supporting Information). The ability to weld PULCE films enables freeform combination of various emissive units, allowing customized phosphorescent maps that remain completely hidden under daylight but emerge in the dark. While the current demonstration employed a basic four‐block arrangement, this strategy can be readily scaled to embed more complex optical data such as QR codes or barcodes for privacy‐sensitive labeling.

**Figure 5:**
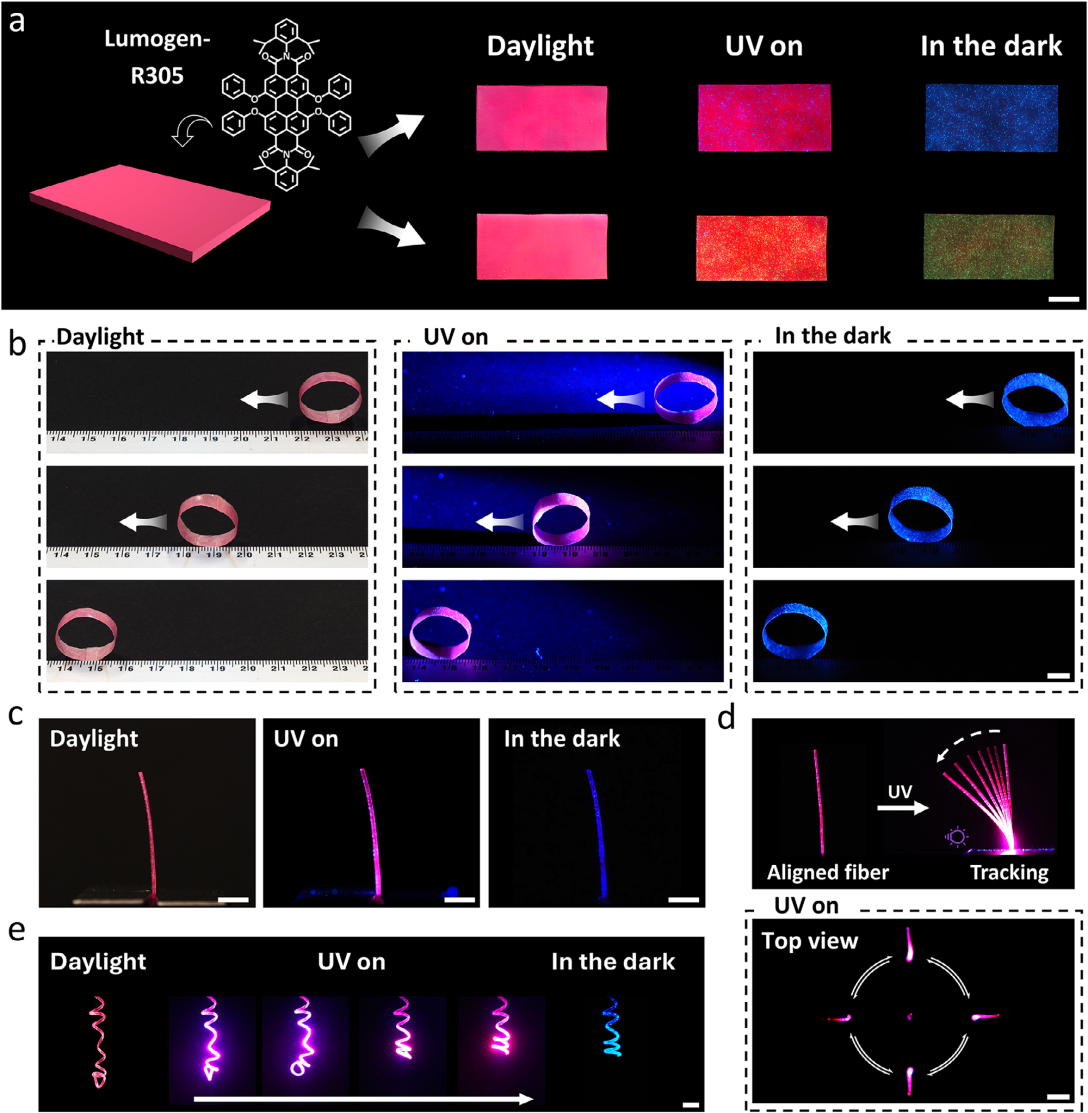
a) Photographs of PULCE‐B and PULCE‐G films doped with the fluorescent dye Lumogen R305 under daylight, UV illumination, and in the dark. The chemical structure of Lumogen R305 is shown inset (scale bar = 5 mm). b) Photographs of the self‐signaling rolling wheel under daylight (left), UV illumination from the right (middle), and in the dark exhibiting its phosphorescent emission and showing its position after movement (right) (scale bar = 5 mm, total time = 30 s). c) Photographs of a luminescent fiber under daylight (left), UV excitation (middle), and in the dark (right) (scale bars = 2 mm). d) Photographs showing UV illumination inducing bending of a luminescent fiber toward the light source (top). Top‐view images showing the fiber sequentially bending toward four different directions (bottom) (scale bar = 1 mm). e) Photographs of a reprogrammed coiled fiber under daylight (left), UV illumination (middle), and in the dark (right), exhibiting a traceable motion (scale bar = 2 mm).

To expand the optical versatility of the phosphorescent PULCE, a fluorescent dye was incorporated, creating a dual emissive responsive material. As shown in **Figure** **5**a, doping the phosphorescent PULCE with the organic fluorescent dye Lumogen R305 yielded a ternary system exhibiting entirely different visual states depending on external lighting. The films exhibited a deep red color dominated by the fluorescent dye absorption under daylight. Under UV irradiation, an intense fluorescent red emission appeared, with subtle fluorescent green or blue emission from the green or blue phosphors (Figure S38a, Supporting Information). Upon removing the UV source, the fluorescence decayed instantaneously, exhibiting the persistent afterglow of either green or blue‐dominated phosphorescence (Figure S38b, Supporting Information). This sequential transition between daylight, excitation, and afterglow states enables a single film to encode various visual optical information under different environments. Importantly, the incorporation of the fluorescent dye did not influence the rearrangement of the supramolecular network. Both PULCE‐G and PULCE‐B composites were successfully programmed into monodomain actuators via hydrogen bonding reconfiguration, exhibiting ≈30% reversible actuation strain between 25 and 60 °C (Figures S39 and S40, Supporting Information). The glowing films also preserved their photo‐thermal actuation behavior, enabling deformation triggered by localized UV irradiation.

Expanding beyond 2D light‐emitting sheets, a 3D self‐signaling luminescent moving actuator whose motion could be tracked in the dark without any external sensors was designed. Using the aligned PULCE‐B film, a rolling wheel was fabricated by bonding the ends of the strip together (Figure S41, Supporting Information). When exposed to UV light, the actuator exhibited an intense fluorescent red, while emitting a blue afterglow upon removing the light source, defining its geometry in the dark. As shown in Figure 5b and Video S7 (Supporting Information), locally illuminating a focused UV beam at the right side of the rolling wheel induced asymmetric deformation, shifting the center of the gravity and inducing rolling of the wheel. Such behavior demonstrates the actuator's position and deformation can be tracked and recorded optically in real time, even in the dark, unlocking practical opportunities for dynamic signaling devices and self‐reporting robotic components. Extending the same design to PULCE‐G film (Figure S42 and Video S8, Supporting Information) further highlights the adaptability and possibility of the color‐tunable self‐emitting actuator for adaptive optical encoding or responsive indicators with multicolor emission channels.

To underscore the luminescent PULCE processability, direct ink writing (DIW) was used to transform the luminescent PULCE into fibrous actuators (Figure 5c; Figure S43, Supporting Information). The melt‐extrusion process preserved both the alignment induced by shear force and optical functionality of the matrix, producing continuous fibers that exhibited identical color response to their corresponding film actuators under different light environments. The printed fiber (diameter: 400 µm) maintained ultralong afterglow while showing ≈30 % reversible contraction upon heating to 60 °C (Figure S44, Supporting Information). Illuminated from one side with a UV beam, the fiber exhibited a distinct photothermal bending response, curving toward the incident beam while simultaneously emitting a fluorescent trail (Figure 5d, top). The fiber gradually increased in curvature with light intensity, reaching a maximum bending angle of ≈45° at 130 mW cm^−2^ (Figure S45a, Supporting Information). When the beam was removed, the fiber left behind a fading luminous memory of where the light stimulus had been. The fiber maintained consistent bending performance after 50 actuation cycles, indicating robust photothermal stability (Figure S45b, Supporting Information). The fiber actuator not only followed the light but also encoded the path of excitation into a visible afterglow that remains even after the stimulus is removed. The directional bending recorded in the top‐view images (Figure 5d, bottom) demonstrates the potential for encoding spatial light traces directly into the material's geometry and emission, as the fiber bends predictably in response to the spatial movement of the UV source. Such a combination of light‐guiding actuation and phosphorescent feedback is particularly promising for autonomous navigation systems, dynamic photonic circuits, and optical communication devices where both movement and data transmission can be adaptively rewritten on demand.

The printed glowing fiber actuator can be reprogrammed into a coiled shape by winding it with a controlled pitch around a metal rod and annealing at 130 °C to allow network reconfiguration (Figure S46a, Supporting Information). Upon cooling, the fiber retained the coiled shape while maintaining multicolor emission under different light environments, with both thermal response under heat and photo response under UV illumination. When thermally cycled across 60 °C in the dark, the coiled fiber exhibited ≈30% contraction, visually transforming mechanical motion into an optical signal (Figure S46b, Supporting Information). Under local UV excitation, the initially loosely coiled fiber shortened, resulting in a visibly tightened helical configuration. The coiled fiber gradually relaxed back to its original length upon removal of the light, with its recovery path traceable in the dark (Figure 5e). Finally, to further highlight the processability and architectural versatility of the luminescent PULCE system, a previously reported vacuum thermoforming approach was used to fabricate a glowing hemisphere (Figure S47, Supporting Information).^[^ ^62^ ^]^ The resulting dome emitted an even, persistent phosphorescence across its entire surface in the dark, similar to the planar actuators and fibers, highlighting the potential of glowing actuators in applications where simple or complex geometries are required.

## 3 Conclusion and Outlook

We have demonstrated stimuli‐responsive luminescent materials by integrating thermoplastic liquid elastomer actuators with inorganic ultralong afterglow phosphors. These materials not only emit persistent green and/or blue afterglow in the dark but also offer multiple pathways for programmable and reversible information encryption. Utilizing maskless and direct hand‐written encoding, we achieved erasable and rewritable phosphorescent patterns via thermal activation. The polydomain phosphorescent films enable switchable transitions between optically scattering and transparent states, modulated by mechanical stretching, heating, or light. Building on this strategy, we have developed a composite system capable of storing and dynamically revealing encoded information. Beyond static displays, we transformed phosphorescent films into programmable actuators that preserve their afterglow while achieving reversible deformation up to 30% under heat (60 °C) or UV light. Through hot welding, we have constructed an encoded bicolored phosphorescent actuator exhibiting a visually indistinguishable appearance under daylight yet detectable in the dark by the region‐specific emissions. Furthermore, by incorporating a fluorescent dye, we expanded the optical versatility of the phosphorescent film to create distinct optical output under different lighting conditions. With this, we fabricated a luminescent light‐driven rolling wheel capable of self‐locating in the dark, as well as DIW‐printed fiber actuators that track light direction with their recovery path traceable. Our work introduces versatile strategies for highly processable active afterglow soft materials and devices that can be encoded and reprogrammed, with potential applications in dynamic reconfigurable photonic devices, anti‐counterfeiting, visual encryption, and soft robotics.

Looking forward, these glowing materials could find more exotic uses. Their activity is similar to bioluminescence found among various living creatures, including the firefly and the angler fish,^[^ ^52^ ^]^ and could be employed for inter‐robot communication. Since the luminophores may be incorporated into LCEs without disrupting their function, one could also envision using them as traceable markers in micro‐actuators and robot devices,^[^ ^42^ ^]^ normally difficult to detect optically, but now providing a means of observation in the dark.

## 4 Experimental Section

### Materials

1,4‐Bis‐[4‐(3‐acryloyloxypropyloxy)benzoyloxy]‐2‐methylbenzene (**1**, ≥97%) was obtained from Merck. 4‐(6‐(acryloyloxy)hexyloxy)phenyl‐4‐(6‐ (acryloyloxy)hexyloxy) benzoate (**2**, ≥97%) was purchased from Synthon. 2,2′‐(Ethylenedioxy)diethanethiol (**3**, ≥97%), Hexamethylene diisocyanate (**5**, ≥98%), Triethylamine (**6**, ≥99%), and 1,6‐Hexanedithiol (**7**, ≥97%) were purchased from Tokyo Chemical Industry (TCI). Dimethylphenylphosphine (**4**, 99%) and N,N‐Dimethylacetamide (DMAc, ≥99%) were purchased from Sigma–Aldrich. Diethyl ether (Et_2_O, ≥99.5%) and 1,1,3,3,3‐hexafluoro‐2‐propanol (HFIP) were acquired from Biosolve. Phosphor materials were supplied by Glowup, Jolin Corporation (China). UV absorber (Tinuvin 328) and fluorescent dye (Lumogen R305) were sourced from BASF. Chloroform‐d (CDCl_3_, 0.03% v/v TMS, 99.8 atom% D) for proton nuclear magnetic resonance (^1^H‐NMR) was purchased from Sigma‐Aldrich. All reagents were used as received without any further purification. The chemical structures of the main component structures can be found in Figure S1 (Supporting Information).

### Characterizations

^1^H‐NMR spectra were acquired using a Bruker Avance III HD NanoBay 400 MHz. Samples were prepared using a solvent mixture of CDCl_3_ and HFIP (95/5% v/v). Fourier‐transform infrared (FT‐IR) analyses were conducted utilizing a Varian 670 IR spectrometer equipped with an attenuated total reflectance (ATR) accessory. Spectra were recorded from 3500 to 700 cm^−1^ with 50 scans at a spectral resolution of 4 cm^−1^. Differential scanning calorimetry (DSC) analyses were performed utilizing a TA Instruments Q2500 DSC instrument equipped with a cooling accessory from −50 to 200 °C at 10 °C min^−1^ under nitrogen in hermetic T‐zero aluminum sample pans with 10 mg product. Transition temperatures were determined from the third cycle. Thermomechanical characterization was made via dynamic mechanical thermal analysis (DMTA) using a TA Instruments Q800. The analysis was performed on a compression‐molded sample (5.0 × 5.0 × 0.5 mm^3^) in vertical tension mode. Thermographs were collected between −50 and 200 °C at a heating rate of 5 °C min^−1^ with 0.01 N preload force, 10 µm amplitude, and a 1 Hz oscillating frequency. Stress–strain curves were recorded on a Zwick/Roell universal testing system equipped with a 1000 N load cell. Tests were conducted at 25 °C under a constant strain rate of 500 mm min^−1^, with an initial preload of 0.1 N. Thermogravimetric analysis (TGA) was conducted on a NETZSCH STA 499 Jupiter system using polymer samples 40 ± 0.5 mg heated from 25 to 800 °C at 5 min^−1^. Gel permeation chromatography (GPC) was carried out on an Agilent 1260 Infinity II system equipped with a PSS PFG column (7 µm, 8 × 50 mm) and two PFG linear XL columns (7 µm, 8 × 300 mm) in series at 40 °C. 1,1,3,3,3‐hexafluoro‐2‐propanol (HFIP) containing potassium trifluoroacetate (3 g L^−1^) served as the mobile phase at a flow rate of 1.0 mL min^−1^. Samples were prepared at a concentration of 1 mg mL^−1^ in HFIP, with 200 ppm toluene included as a flow marker. Polarized optical microscopy (POM) analyses were performed on a Leica DM2700 M equipped with crossed polarizers and a Linkam THMS600 hot stage for temperature control. 2D wide‐angle X‐ray scattering (WAXS) was performed on a Ganesha instrument from SAXSLab. The flight tube and sample holder are all under vacuum in a single housing, with a GeniX‐Cu ultra‐low divergence X‐ray generator. The source produces 0.154 nm X‐rays with flux of 1×10^8^ p s^−1^. Scattered X‐rays were captured on a 2D Pilatus 300 K detector with 487 × 619 of 172 × 172 µm^2^ pixel resolution. The sample‐to‐detector distance was 0.084 m (WAXS mode). Scanning electron microscopy (SEM) images were obtained using an FEI Quanta 3D FEG operating in secondary electron mode. Samples were sputter‐coated with a gold layer before imaging using Q150T (Quorum). The absolute luminance of the phosphor was measured using a Konica Minolta LS‐150 luminance meter. Ultraviolet–visible (UV–vis) transmission and absorption spectra were acquired using a PerkinElmer Lambda 750 spectrophotometer. Fluorescent spectra, phosphorescent spectra and lifetime spectra were measured by using an AVANTES Spectrometer (AvaSpec‐ULS2048CL‐EVO). UV illumination was accomplished using a 365 nm LED (Thorlabs M780LP1) and driver (Thorlabs DC2200). Temperatures of the samples were monitored by a Fluke Ti32 Infrared Camera. Photographs and videos were taken with a digital camera (Olympus OM‐D E‐M10 Mark III) in manual mode and an Apple iPhone smartphone.

### Synthesis of the PULCE

The PULCE was prepared following a modified method detailed previously.^[^ ^53^ ^]^ Specifically, diacrylate mesogens **1** and **2** were first fully dissolved in DMAc (50 wt%) under an argon atmosphere at 50 °C. Upon cooling the solution to room temperature, the dithiol chain extender **3** was added under continuous stirring, followed by the addition of a nucleophilic catalyst **4** (0.1 wt%). The mixture was left to react at room temperature for 2 h. Subsequently, a solution of diisocyanate **5** in DMAc (50 wt%) was added, immediately followed by the base catalyst **6** (0.1 wt%). The mixture was stirred at room temperature for 15 min. As viscosity increased, an extra portion of DMAc (30 wt%) was added. Dithiol **7** was then added dropwise; once the addition was complete, the reaction mixture was maintained at 60 °C and left to proceed overnight. Following the reaction, the viscous mixture was slowly poured into cold Et_2_O (500 mL) while stirring, precipitating the polymer as a granular solid. The crude solid was then transferred to fresh Et_2_O and stirred for a further overnight to remove residual solvent. Finally, after decanting the solvent, the final polymer was dried under vacuum at 40 °C. The obtained product appeared as a white solid with an overall yield exceeding 97%.

### Fabrication of the PULCE Film Actuator

To prepare an aligned PULCE film actuator, the dried PULCE granulate was first sandwiched between two sheets of polytetrafluoroethylene (PTFE) film and then placed between the plates of a hot press (Tribotrak), where the temperature was set to 170 °C. A pressure of 10 kg was applied and maintained for 5 min to ensure complete melting of the polymer. Upon removal from the hot press, the polymer was allowed to cool to room temperature, yielding an optically scattering and smooth film. Subsequently, the polymer film was cut into a rectangular shape (1 × 2 cm^2^) and uniaxially stretched to 200% at room temperature. While maintaining this elongation, the sample was transferred into a preheated oven set at 130 °C and annealed for 30 min. Finally, the film was rapidly cooled to room temperature, resulting in a well‐aligned PULCE film.

### Preparation of the Phosphorescent PULCE Film

PULCE granules were initially dissolved at room temperature in HFIP (1 g/15 mL). Once complete dissolution was achieved, phosphors (2.0 wt%) and UV absorber (1.0 wt%) were directly added to the polymer solution to make a homogeneous dispersion. While continuously stirring, the solvent was gradually evaporated until two‐thirds of its volume had been removed to reach a moderate viscosity. The mixture was then transferred into a large PTFE petri dish to facilitate further solvent removal. Once the surface of the mixture appeared dry, the petri dish was placed in a vacuum oven at 40 °C overnight to ensure complete removal of the residual solvent, resulting in a PULCE film with phosphors evenly distributed throughout the polymer matrix.

### Maskless Photopatterning Encoding Process

To encode phosphorescent patterning, a digital micromirror device (DMD) was used. Specifically, a binary image corresponding to the desired pattern was imported into the DMD system, where each micromirror can be individually switched between “on” and “off” states according to the input image. The mirrors in the “on” state selectively reflect UV light to form a spatially modulated illumination, which is then projected onto the film. As a result, precise and complex phosphorescent patterns could be readily written onto the film without a physical mask.

### Hand‐Written Encoding and Erasing Process

The hand‐written encoding and erasing experiments were carried out using PULCE film (3.0 × 3.0 cm^2^, thickness: 150 µm) with green phosphors prepared via hot pressing. The film without daylight or UV exposure was placed in the dark. A UV LED light source, combined with a condenser lens, was employed as an excitation beam. By moving the lens assembly across the surface, individual letters “S”, “F” and “D” in this demonstration were written directly onto the film. During the writing process, the paths traced by the UV light could be observed in real‐time. To erase the written patterns, the film was transferred onto a hot stage at 80 °C. Once the patterns had been completely erased, the sample was allowed to cool back to room temperature. Following cooling, the film could be re‐addressed, enabling multiple write‐erase cycles.

### Mechanical Stretching of the Polydomain Phosphorescent PULCE Film

A polydomain phosphorescent PULCE film (thickness: 700 µm) was fixed within a uniaxial stretching device. A white paper with the printed red characters “SFD” was placed directly behind the film. In the undeformed state, the underlying message cannot be observed either under daylight or in the dark. The film was then gradually stretched up to 100% strain. During deformation, the film thinned and became transparent. The hidden “SFD” text became clearly visible through the film both under daylight and under the film's phosphorescent emission in the dark.

### Encoding Process of the Phosphorescent PULCE Composite System by Hot Pressing

To encode information, a hot‐pressed polydomain PULCE film doped with blue phosphors (1.5 × 2.5 cm^2^, thickness: 700 µm) was used as the base substrate. Additionally, two separate polydomain PULCE films doped with green phosphors were precision‐cut into the shapes of the letters “H” and “I” (each 1.0 × 1.0 cm^2^, thickness: 100 µm). The “H” film was placed on the front left of the blue‐phosphor‐doped base film, while the “I” film was placed on the back right. The stacked films were sandwiched between two sheets of PTFE to prevent adhesion and then hot pressed at 180 °C under 2 kg pressure for ≈3 min, allowing complete melting and fusion of the polymer layers. Upon cooling to room temperature, the resulting film exhibited a smooth surface and a white, scattering appearance under daylight.

### Preparation of the Phosphorescent PULCE Film Actuator

Aligned phosphorescent PULCE film actuator was fabricated following the same procedure used for the fabrication of the aligned PULCE film actuator.

### Hot Welding Process and Preparation of the Encoded Bicolored Phosphorescent Film Actuator

For the fabrication of the bicolored phosphorescent film, two PULCE films (1 × 1 cm^2^ each) doped with blue phosphors and two films (1 × 1 cm^2^ each) doped with green phosphors were prepared. The four films were arranged in an alternating pattern and carefully aligned to form a 2 × 2 cm^2^ square shape. This assembly was sandwiched between two PTFE sheets and hot pressed at 180 °C with a pressure of 1 kg for 3 min to ensure complete melting and welding of the adjacent interfaces. The resulting film was then removed from the hot press and cooled to room temperature, yielding a white, smooth film without any visible seams at the junctions between the original segments. To prepare the bicolored phosphorescent film actuator, the same uniaxial alignment procedure described previously was used.

### Preparation of the Luminescent PULCE Doped with Fluorescent Dye

To prepare luminescent PULCE films incorporating fluorescent dye, PULCE granules were first fully dissolved in HFIP within a glass vial. A UV absorber (1.0 wt%), fluorescent dye (0.01 wt%), and phosphor (2.0 wt%) were then gradually added while stirring. The solution was further mixed for an additional 30 min to ensure uniform dispersion of all components. The solvent was allowed to evaporate until two‐thirds of its volume had been removed, after which the mixture was cast into a large PTFE petri dish until the surface appeared dry. Finally, the PTFE petri dish was placed in a vacuum oven at 40 °C overnight to completely remove the residual solvent, yielding luminescent PULCE films with uniformly distributed fluorescent dye.

### Fabrication of the Luminescent Rolling Wheel

The luminescent rolling wheel was fabricated using a uniaxially aligned rectangular luminescent PULCE film (6 × 0.7 cm^2^, thickness: 150 µm). To form the ring geometry, the two ends of the film strip were brought together and bonded using double‐sided adhesive tape, thereby forming a circular loop structure.

### Preparation of the Luminescent PULCE Fiber Actuator

Luminescent PULCE fibers were fabricated via direct ink writing (DIW) using a custom printer (Eindhoven University of Technology, The Netherlands) where the printhead was attached to the moving XY gantry (CoreXY) of a motion system (ToolChanger Motion system, E3D, UK). The printing process was programmed and executed via G‐code. Before printing, luminescent PULCE granules were dried overnight at 40 °C and then loaded into the glass syringe within a ceramic heater (Triangle‐Lab Prusa MK4 CHC kit, China). The syringe and nozzle assembly were heated to 180 °C for 10 min to ensure complete melting of the polymer. Fiber extrusion was driven by an N_2_ pressure of 0.2 MPa through a 0.4 mm nozzle (MK8 nozzle, 123‐3d.nl, The Netherlands) with a printing speed of 10 mm s^−1^, and a single‐layer deposition. The spacing between adjacent printed fibers was set at 1 mm. After printing, the fibers were peeled off the collector plate, yielding uniaxially aligned fibers exhibiting luminescent properties.

### Preparation of the Luminescent Coiled PULCE Fiber Actuator

Luminescent coiled PULCE fiber was prepared by mechanically wrapping a previously printed luminescent PULCE fiber around a 5 mm diameter metal rod, fixing both ends to maintain a uniform 2 mm pitch distance between adjacent coils. The coil was then placed in an oven at 130 °C and annealed for 30 min. Upon removal from the oven and cooling to room temperature, the fiber exhibited a loosely coiled structure.

### Fabrication of the Luminescent PULCE Hemisphere

A luminescent PULCE hemisphere was fabricated using vacuum thermoforming as previously reported.^[^ ^62^ ^]^ A luminescent PULCE film was placed between a top ring and an aluminum mold (6 × 6 × 6 cm^3^) with a 2 cm radius hemispherical cavity coated with PTFE spray. After securing tightly, a vacuum was applied at room temperature until the film formed fully to the cavity shape. Maintaining vacuum, the mold was heated on a 150 °C hotplate for 30 min and then cooled to room temperature. The hemispherical film was subsequently demolded, exhibiting precise replication of the mold structure.

## Conflict of Interest

The authors declare no conflict of interest.

## Supporting information



Supporting Information

Supplemental Video 1

Supplemental Video 2

Supplemental Video 3

Supplemental Video 4

Supplemental Video 5

Supplemental Video 6

Supplemental Video 7

Supplemental Video 8

## Data Availability

The data that support the findings of this study are available from the corresponding author upon reasonable request.
