# Supplementary material for: Stimuli‐Responsive Afterglow from Luminescent Liquid Crystal Elastomers
Source: Adv Mater. 2025 Nov 20;38(7):e16922. doi: 10.1002/adma.202516922 (PMC12862749; doi:10.1002/adma.202516922)
Supplement: Supplementary file 1 — Supporting Information [file ADMA-38-e16922-s003.docx]

Supporting Information

Stimuli-Responsive Afterglow from Luminescent Liquid Crystal Elastomers

Lansong Yue, Michael G. Debije*, and Albert P. H. J. Schenning*

L. Yue, M. G. Debije, A. P. H. J. Schenning

Stimuli-responsive Functional Materials and Devices (SFD)

Department of Chemical Engineering and Chemistry

Eindhoven University of Technology (TU/e)

Groene Loper 3, Eindhoven 5612AE, The Netherlands

E-mail: a.p.h.j.schenning@tue.nl, m.g.debije@tue.nl

M. G. Debije, A. P. H. J. Schenning

Institute for Complex Molecular Systems (ICMS)

Eindhoven University of Technology (TU/e)

Groene Loper 3, Eindhoven 5612AE, The Netherlands

M. G. Debije, A. P. H. J. Schenning

Interactive Polymer Materials (IPM)

Eindhoven University of Technology (TU/e)

Groene Loper 3, Eindhoven 5612AE, The Netherlands

**The Supplementary Information includes:**

Supplementary Text

Figures S1-S47

Videos S1-S8

**Captions for Videos**

**Video S1.**

Thermal actuation of the PULCE film actuator

**Video S2.**

Direct hand-written encoding and heat erasing

**Video S3.**

Intense emission of green/blue phosphorescence

**Video S4.**

Thermal actuation of the PULCE-G/B film actuators in the dark

**Video S5.**

Photo-response of the PULCE-G/B film actuators in the dark

**Video S6.**

Dynamic multicolor phosphorescence under heat/light

**Video S7.**

Controllable movement of the blue-emitting rolling wheel in the dark

**Video S8.**

Controllable movement of the green-emitting rolling wheel in the dark


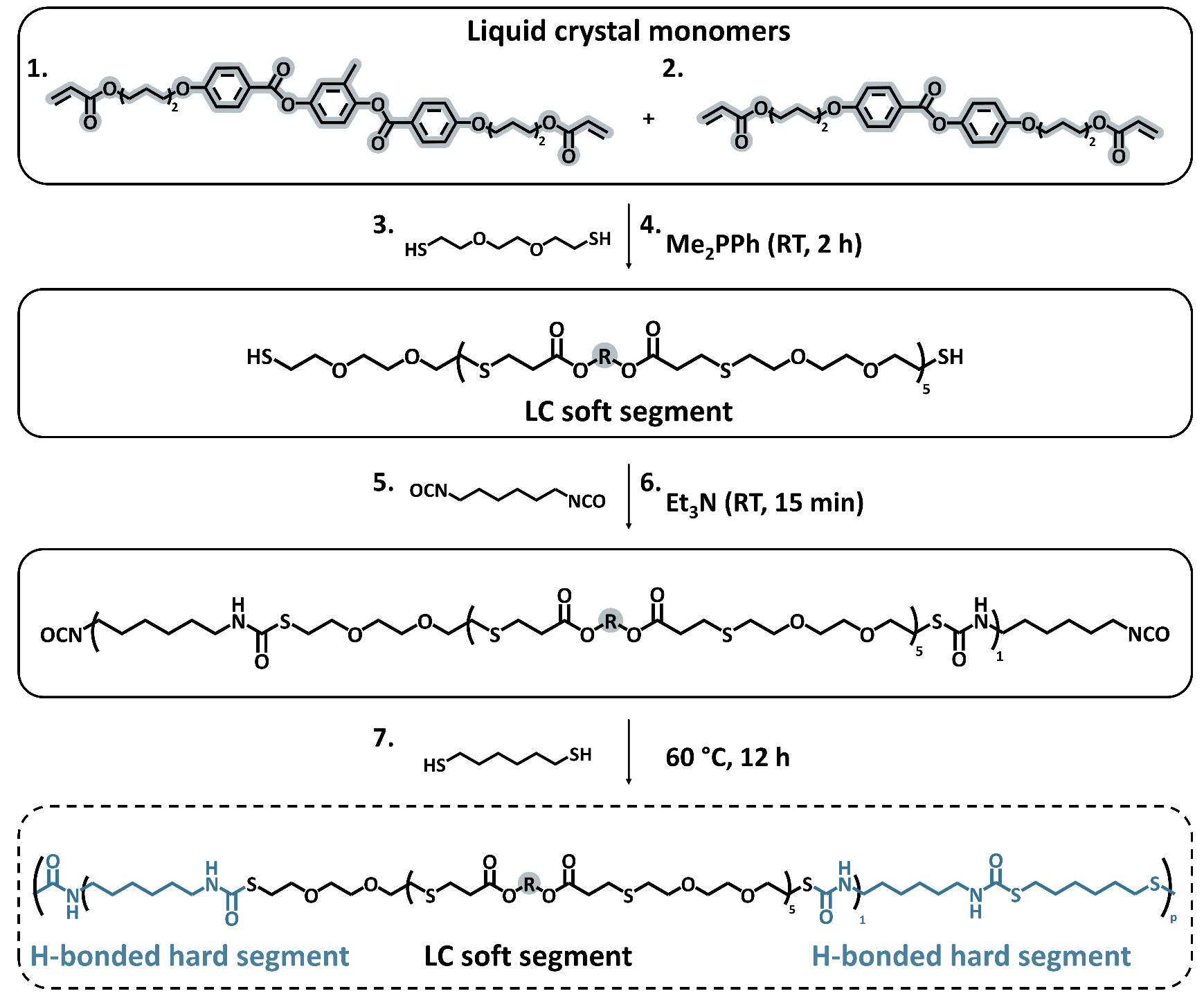


**Figure S1.** Synthesis of the PULCE. The network is constructed via sequential thiol-acrylate and thiol-isocyanate addition, yielding a hydrogen-bonded supramolecular system.

**
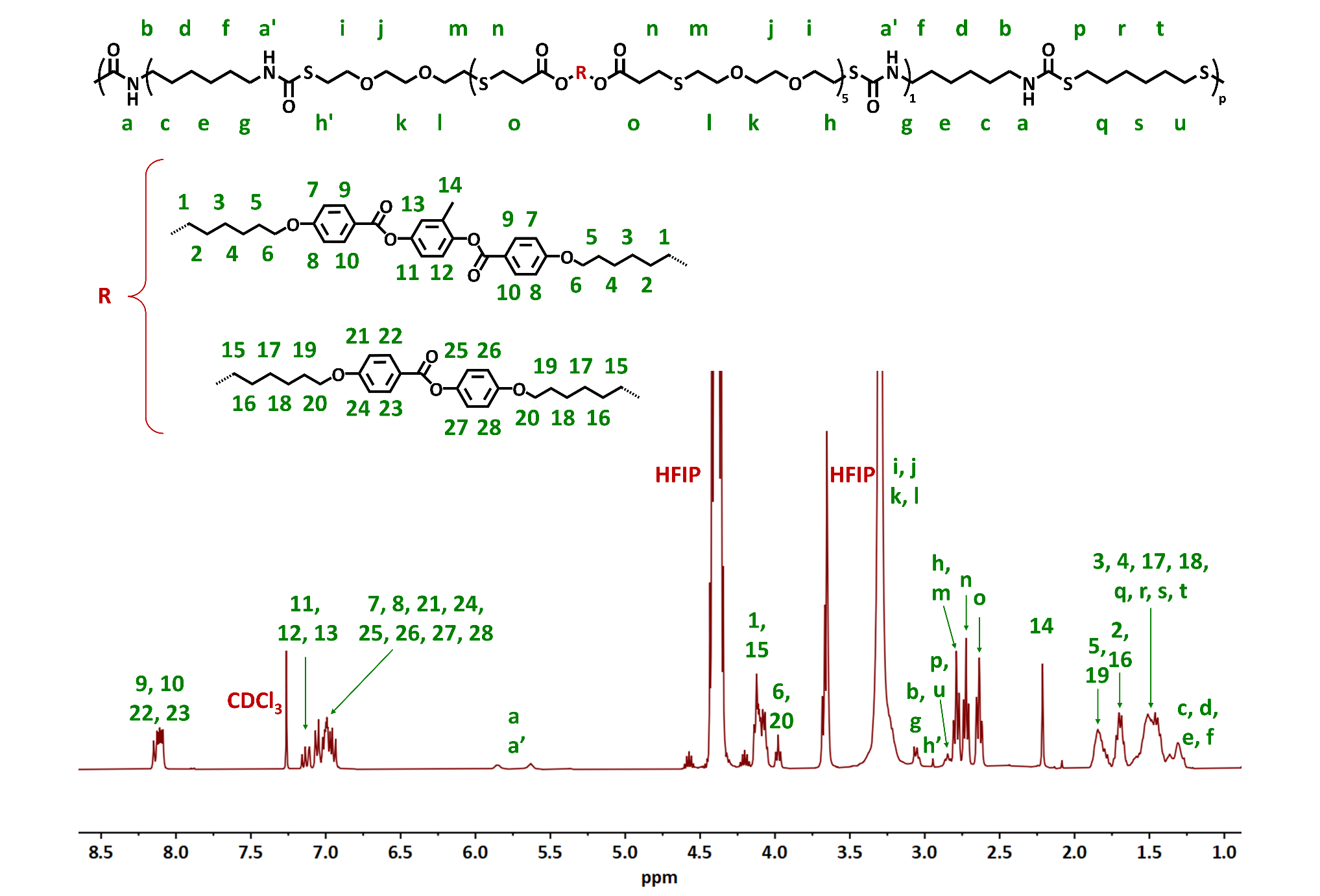
**

**Figure S2.** ^1^H NMR spectrum of the PULCE in CDCl_3_/HFIP (95/5% v/v).


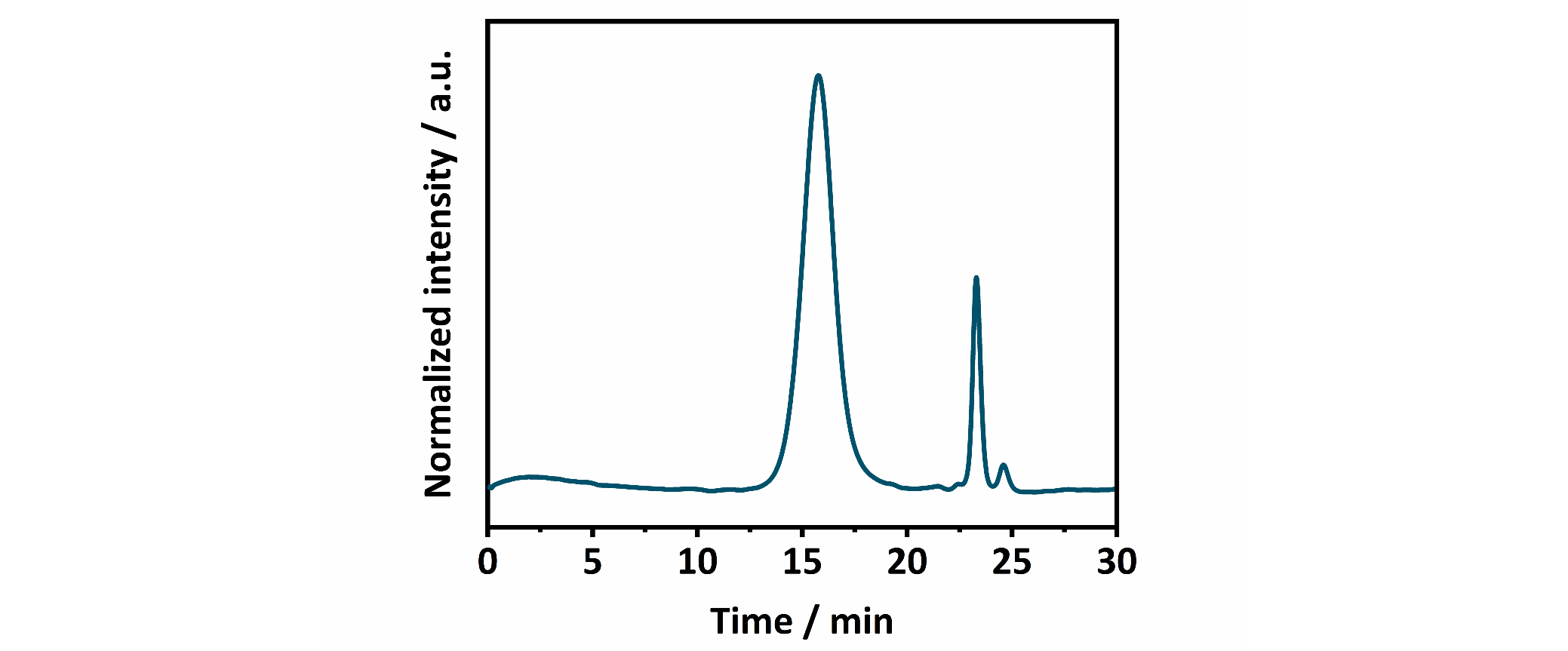


**Figure S3.** Gel permeation chromatography (GPC) curve of PULCE showing a number-average molecular weight (Mn) of approximately 59 kg mol^-1^ with a polydispersity index (PDI) of 1.9. The peak observed around 23 min originates form the toluene used as a flow marker.


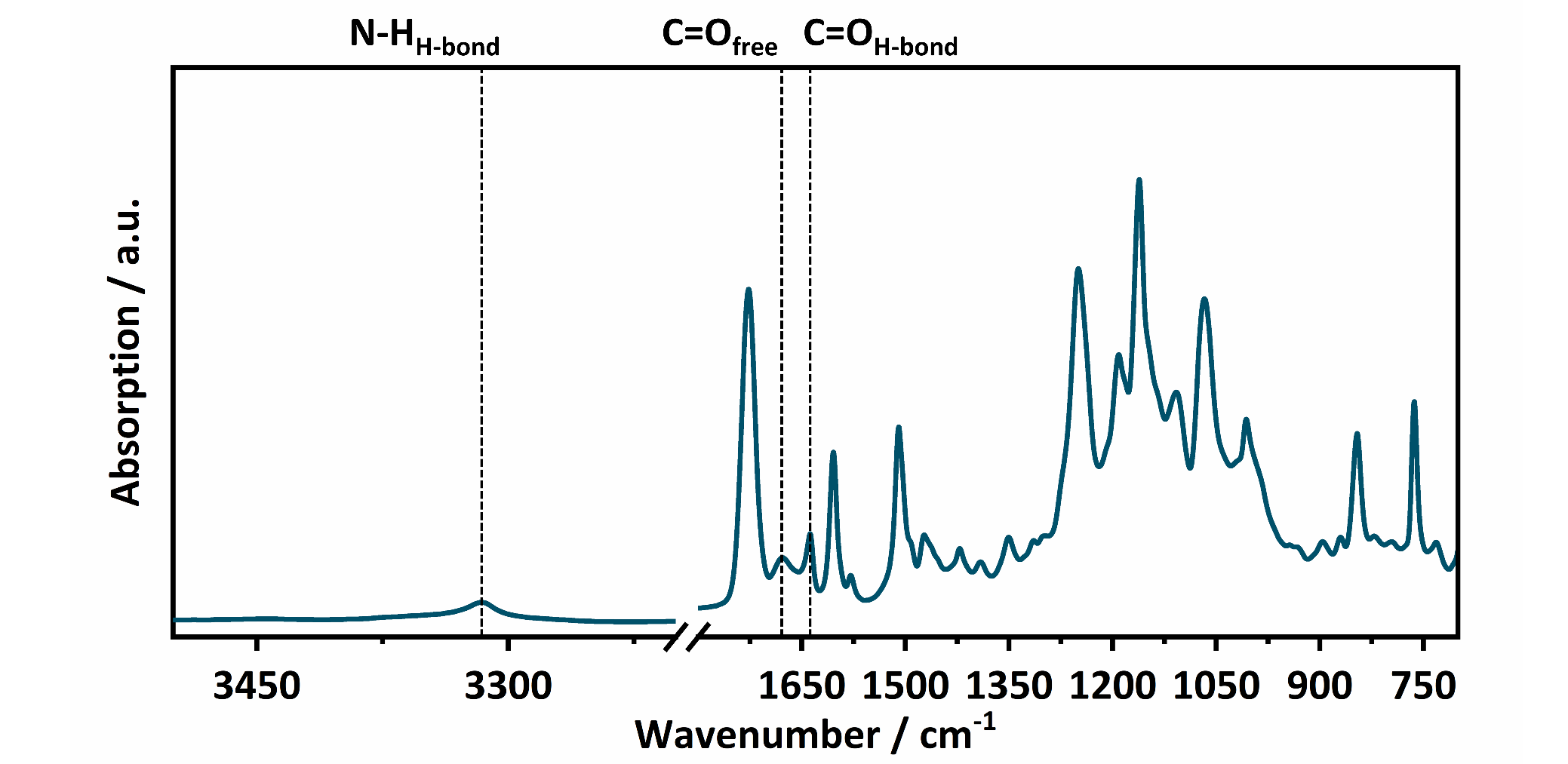


**Figure S4.** Fourier-transform infrared (FT-IR) spectrum of PULCE at room temperature showing hydrogen-bonded amine stretching bands (~3315 cm^-1^) and carbonyl stretching bands (~1640 cm^-1^), confirming the presence of supramolecular interactions.


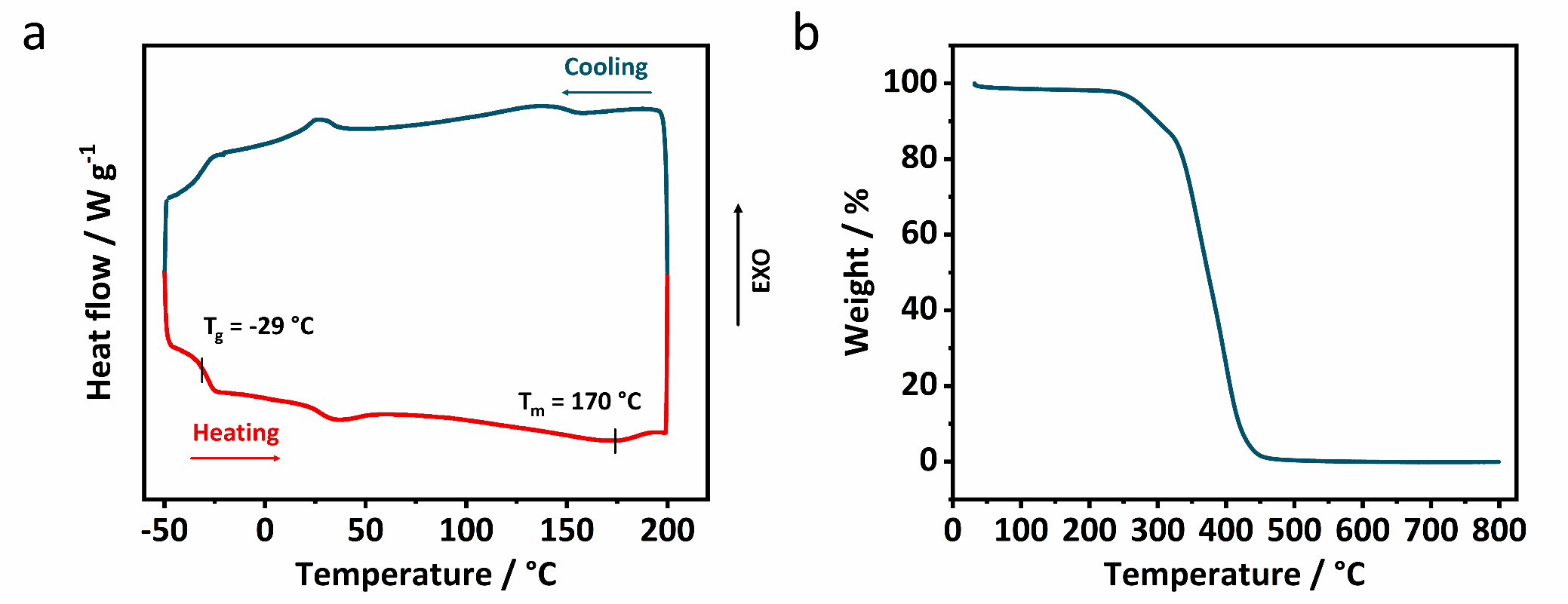


**Figure S5.** a) Differential scanning calorimetry (DSC) of PULCE between -50 and 200 °C showing a glass transition temperature at -29 °C and melting point at 170 °C. b) Thermogravimetric analysis (TGA) curve indicating thermal stability of PULCE with decomposition onset near 260 °C.


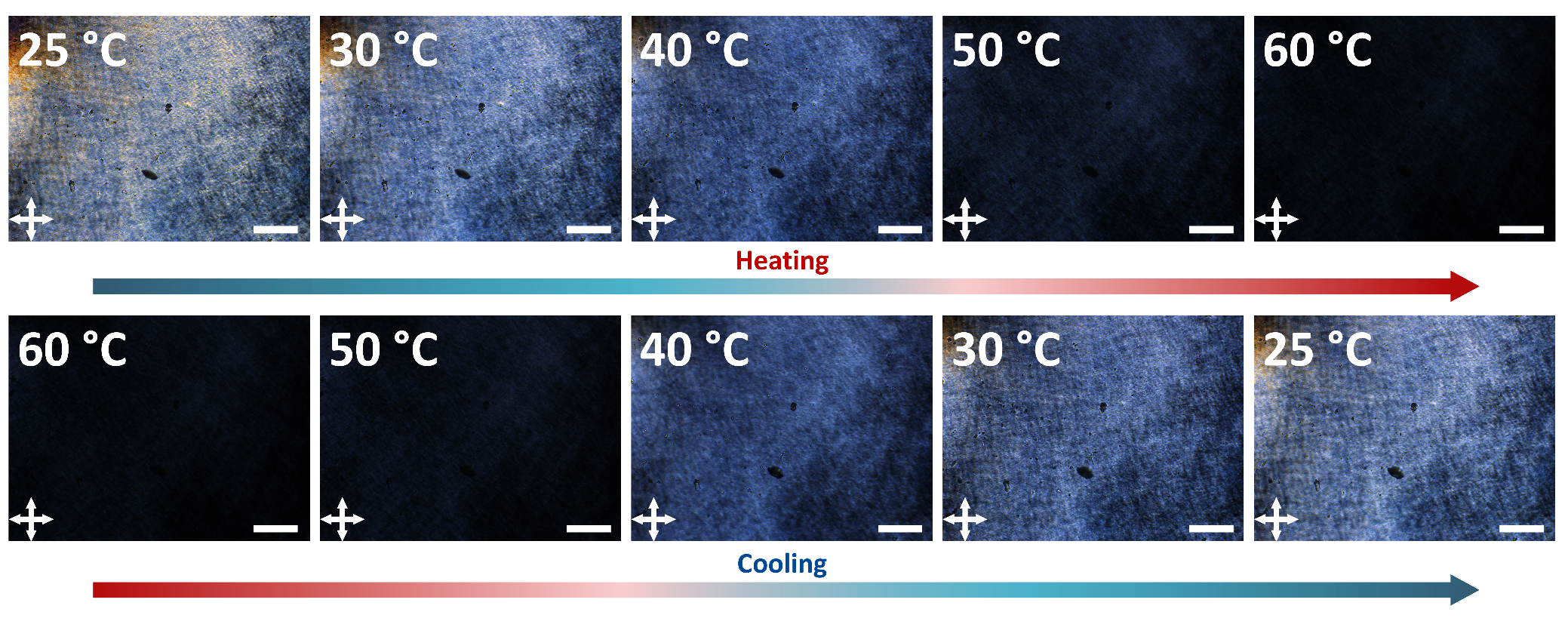


**Figure S6.** Polarized optical microscopy (POM) images of PULCE during heating and cooling between 25 and 60 °C. The disappearance of birefringence near 60 °C marks the nematic-isotropic transition (*T*_ni_) (scale bars = 75 µm).


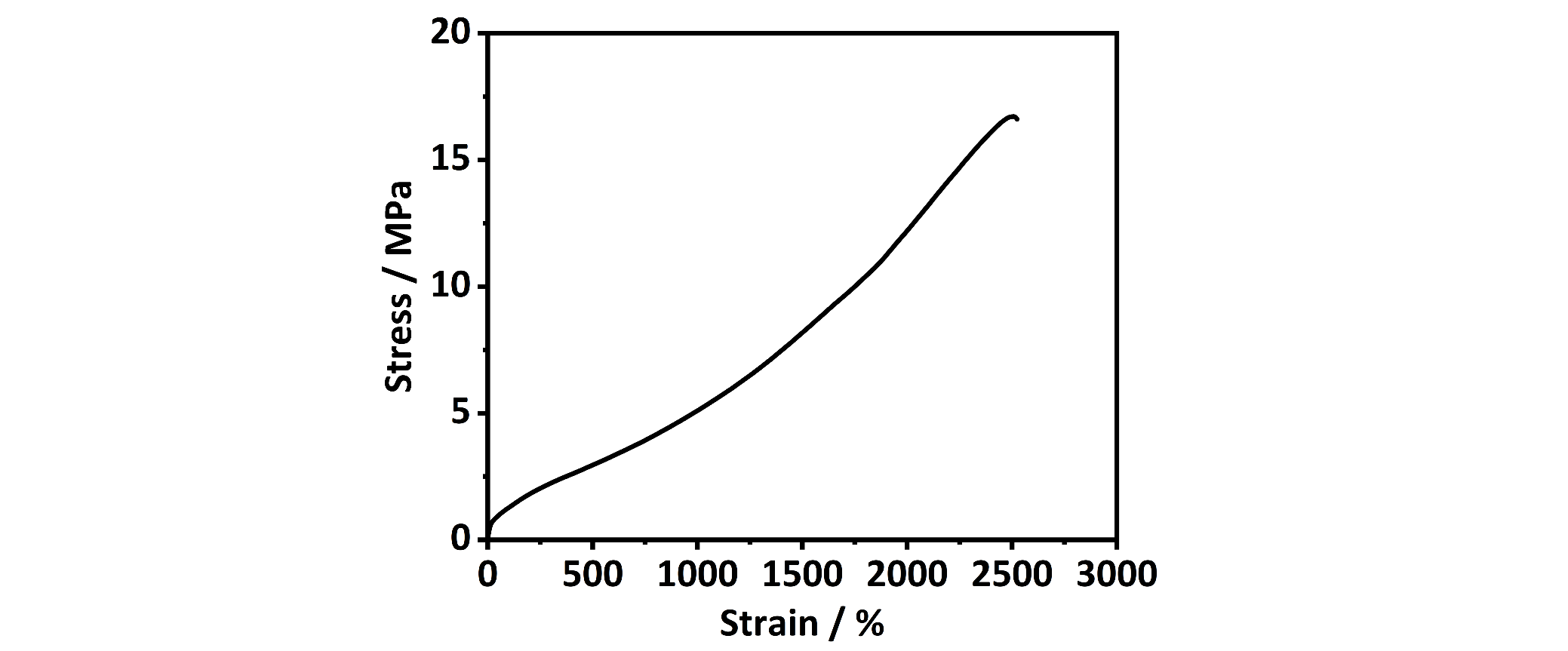


**Figure S7.** Stress-strain curve of PULCE demonstrating elongation exceeding 2500%.


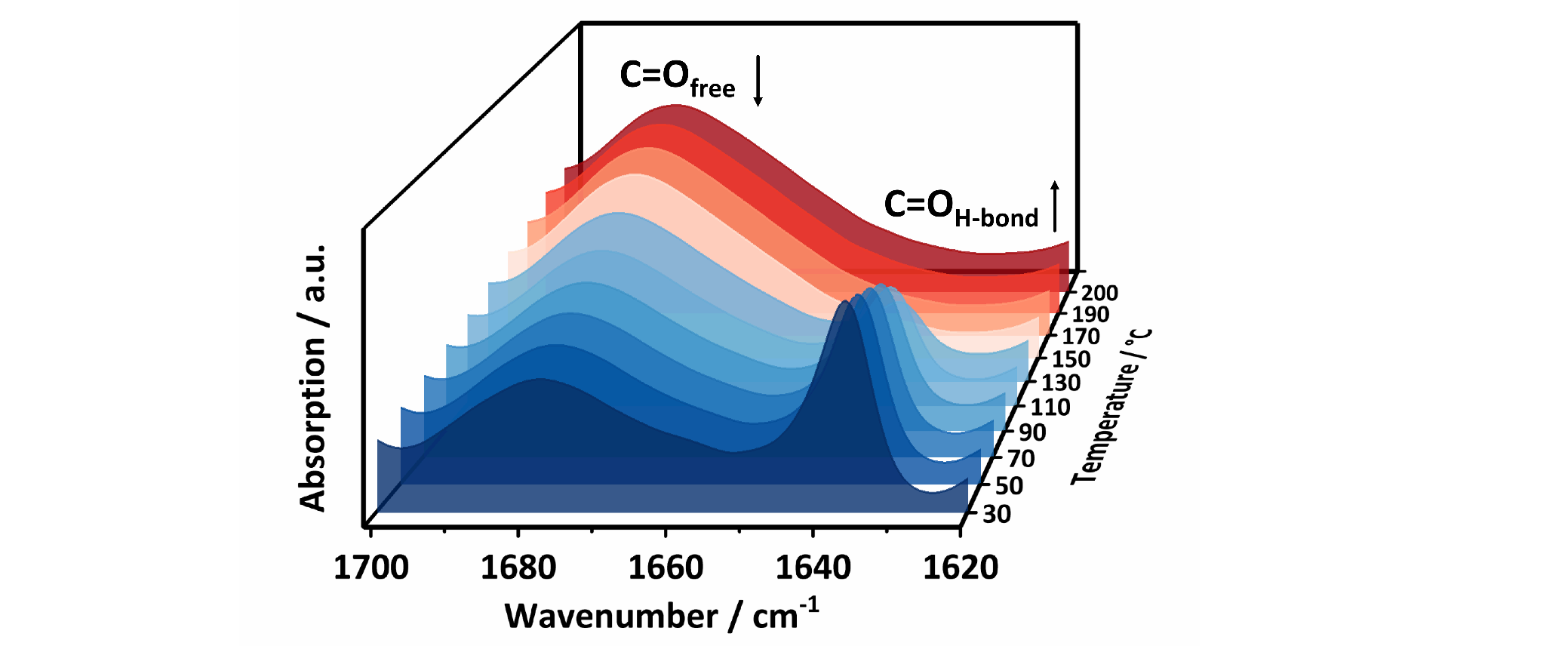


**Figure S8.** Temperature-dependent FT-IR spectra of PULCE during cooling. Gradual reappearance of hydrogen-bonded carbonyl stretching bands and suppression of free carbonyl signals confirm the reformation of supramolecular cross-links.


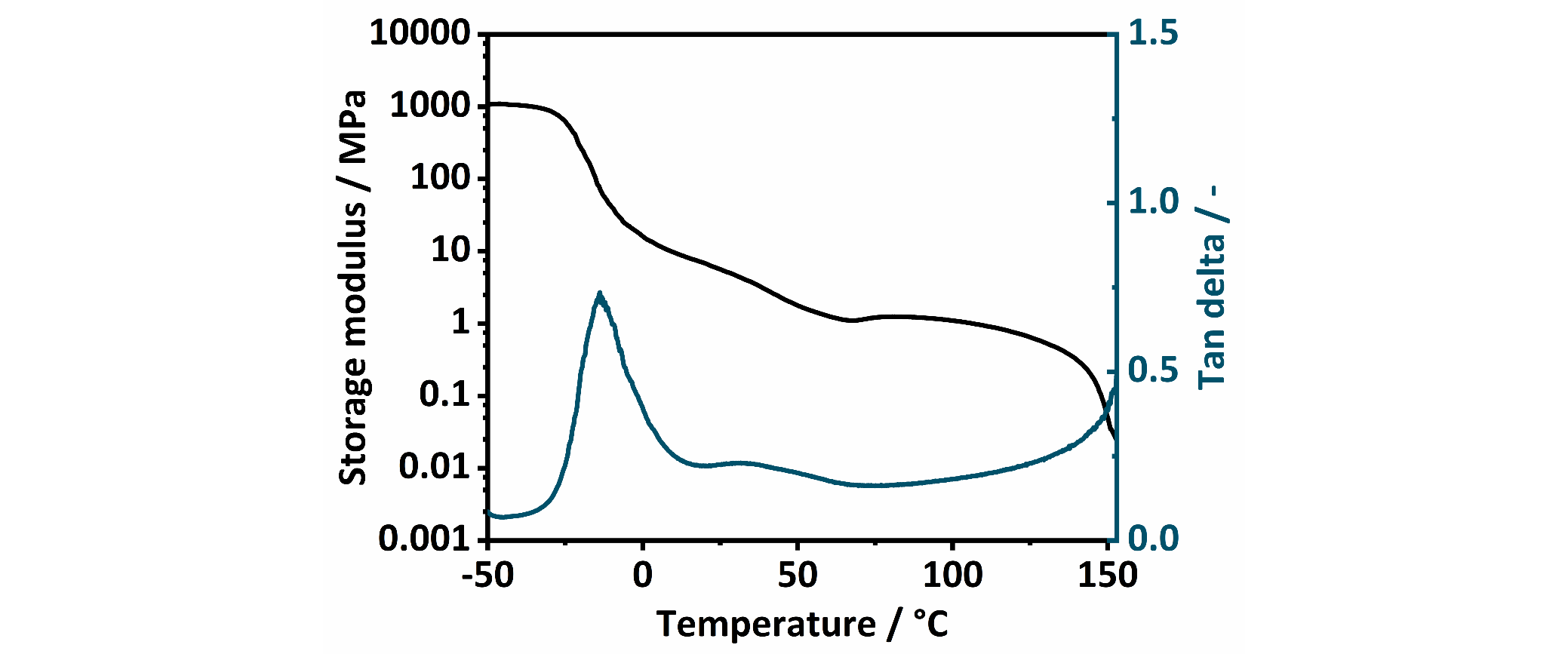


**Figure S9.** Dynamic mechanical thermal analysis (DMTA) of PULCE showing a *T*_g_ below 0 °C, and a modulus drop above 120 °C, corresponding to the onset of hydrogen-bonding dissociation.


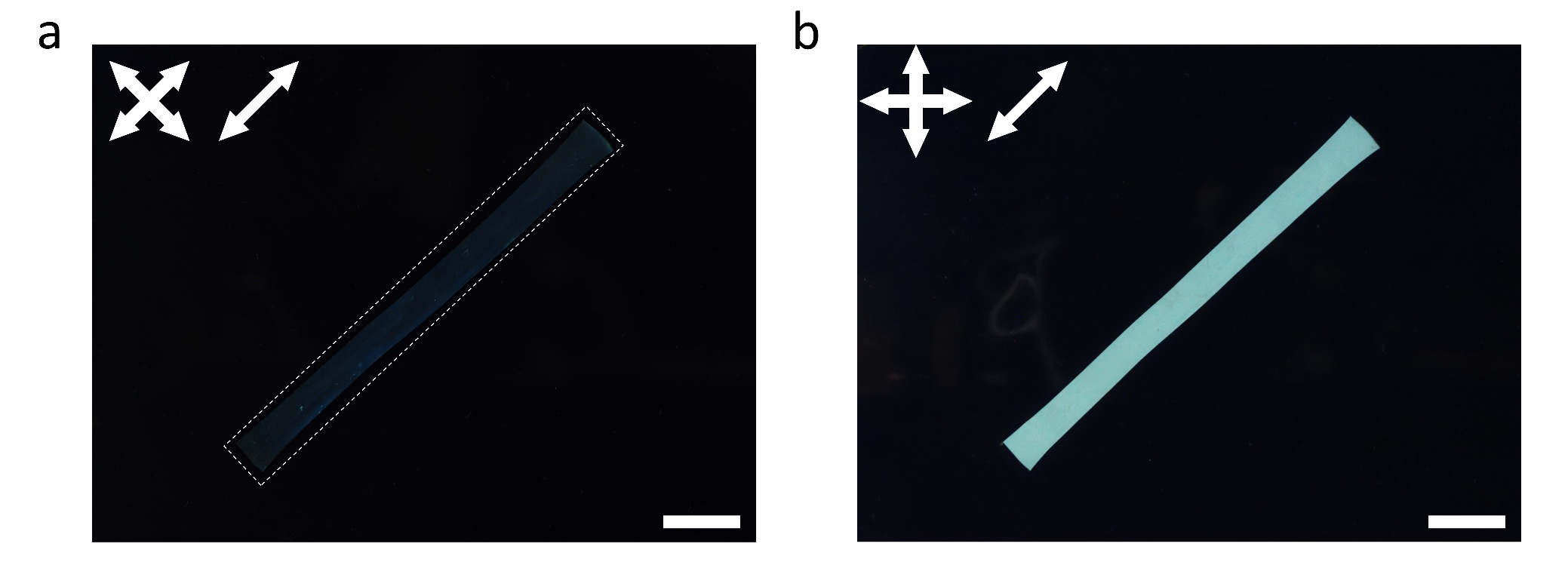


**Figure S10.** Polarized optical microscopy (POM) images of the aligned PULCE film under crossed polarizers a) before and b) after rotating 45° (scale bars = 10 mm). The crossed arrows denote polarizer orientation, and the double headed arrow represents the stretching direction.


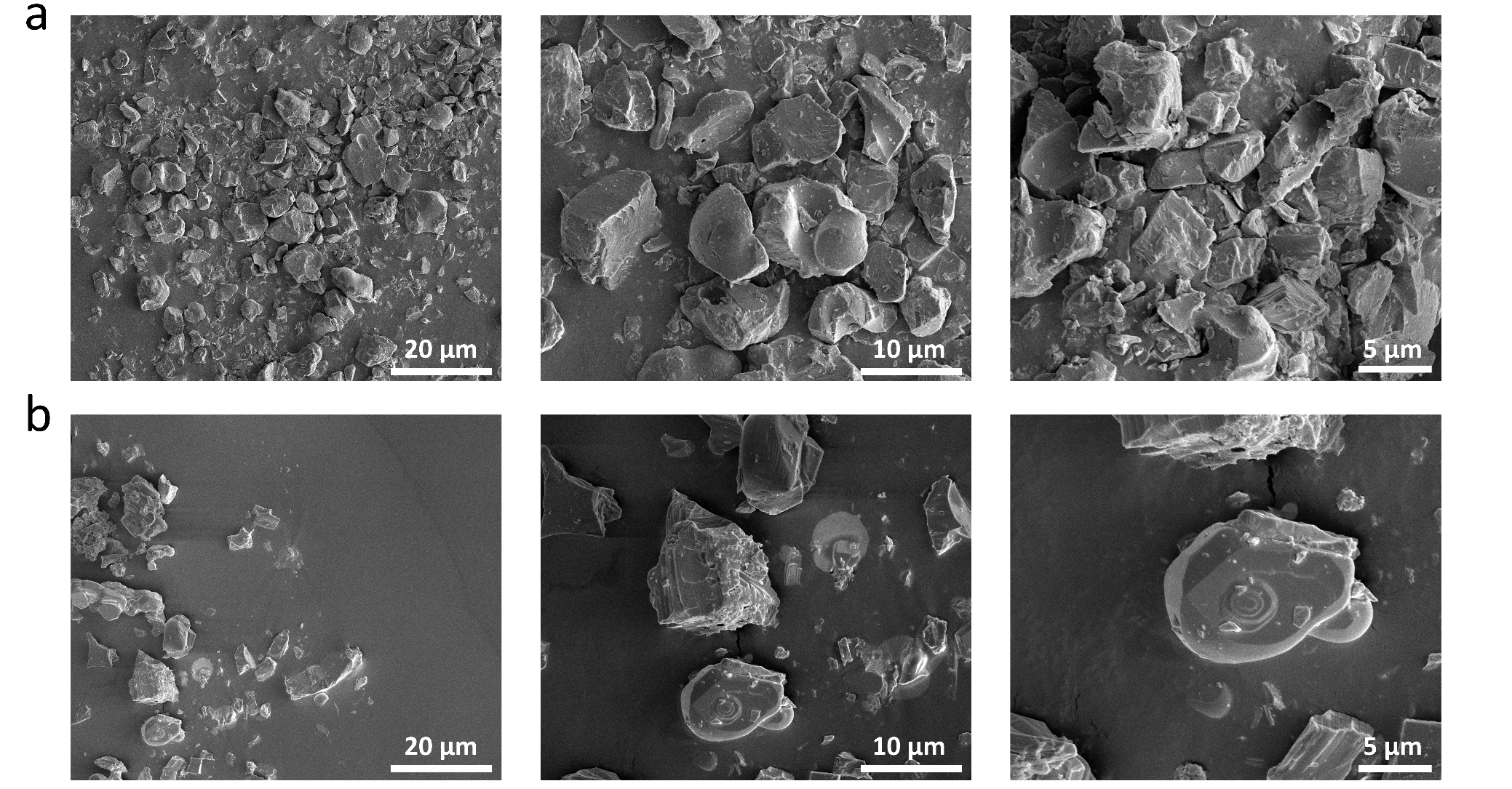


**Figure S11.** Scanning electron microscopy (SEM) images of the a) green phosphor with an average particle size of 8 ± 2 μm, and b) blue phosphor with an average size of 15 ± 5 μm.


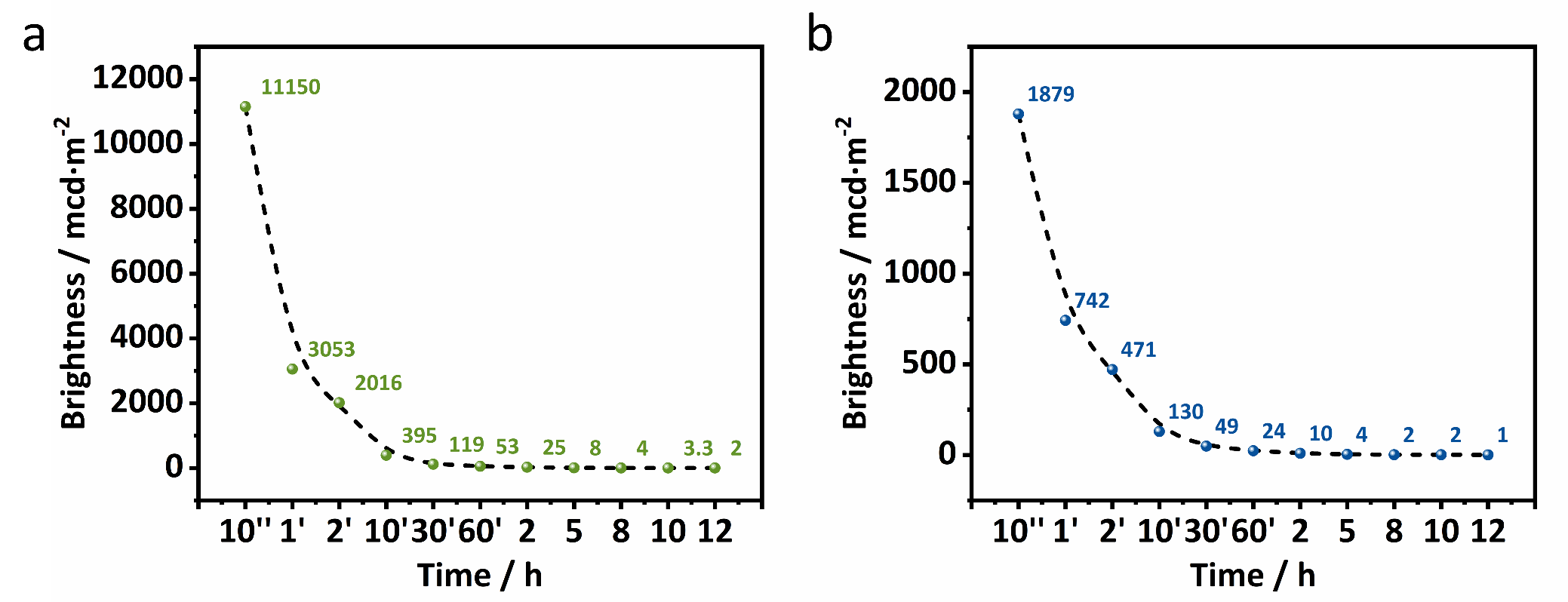


**Figure S12.** Phosphorescence decay curves of the a) green and b) blue phosphors at room temperature. Both phosphors demonstrate persistent afterglow up to 12 h in the dark.


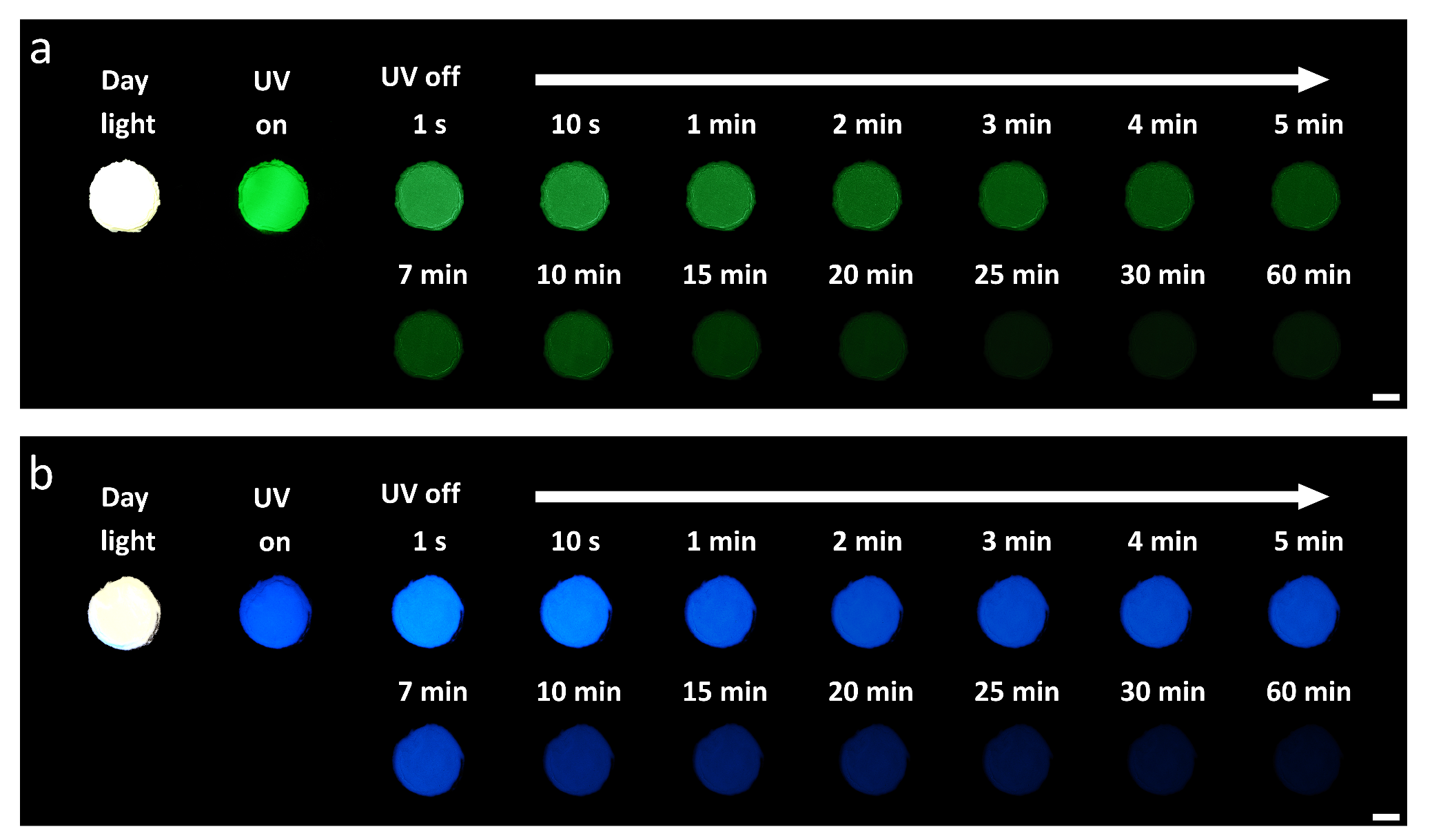


**Figure S13.** Photographs of the a) green and b) blue phosphors under daylight, UV excitation, and after UV-off.


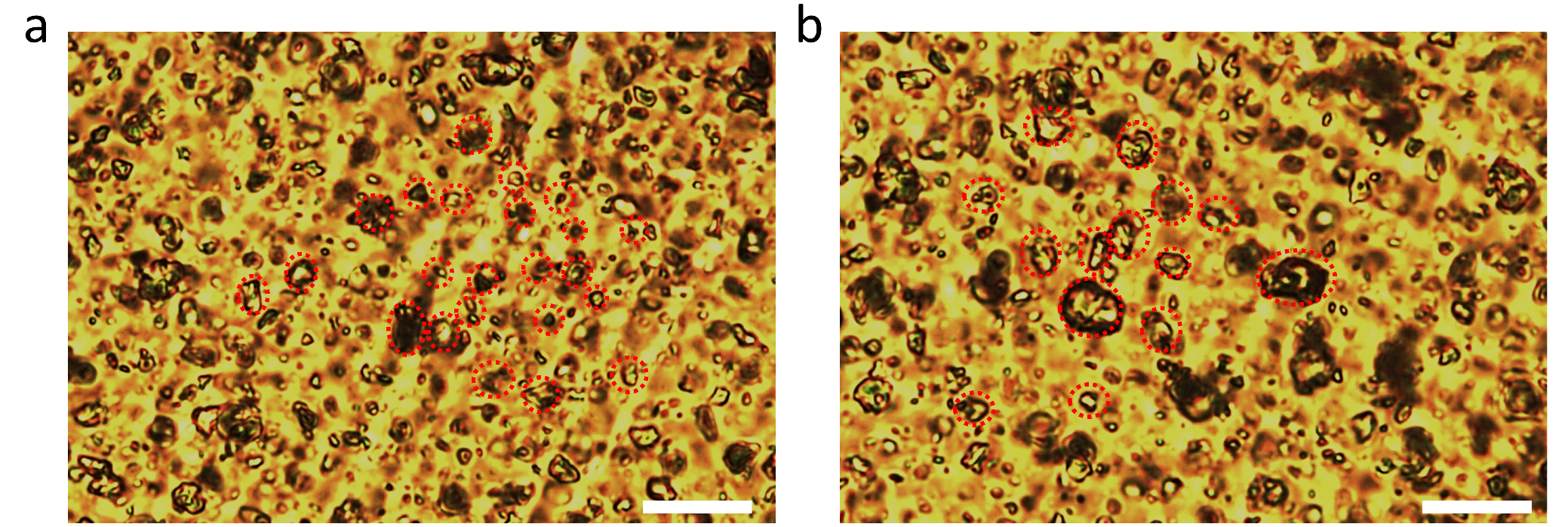


**Figure S14.** Optical microscopy images of a) PULCE-G and b) PULCE-B films showing the dispersion of the phosphor particles (scale bars = 20 µm). Red dashed circles denote representative phosphor particles embedded in the matrix.


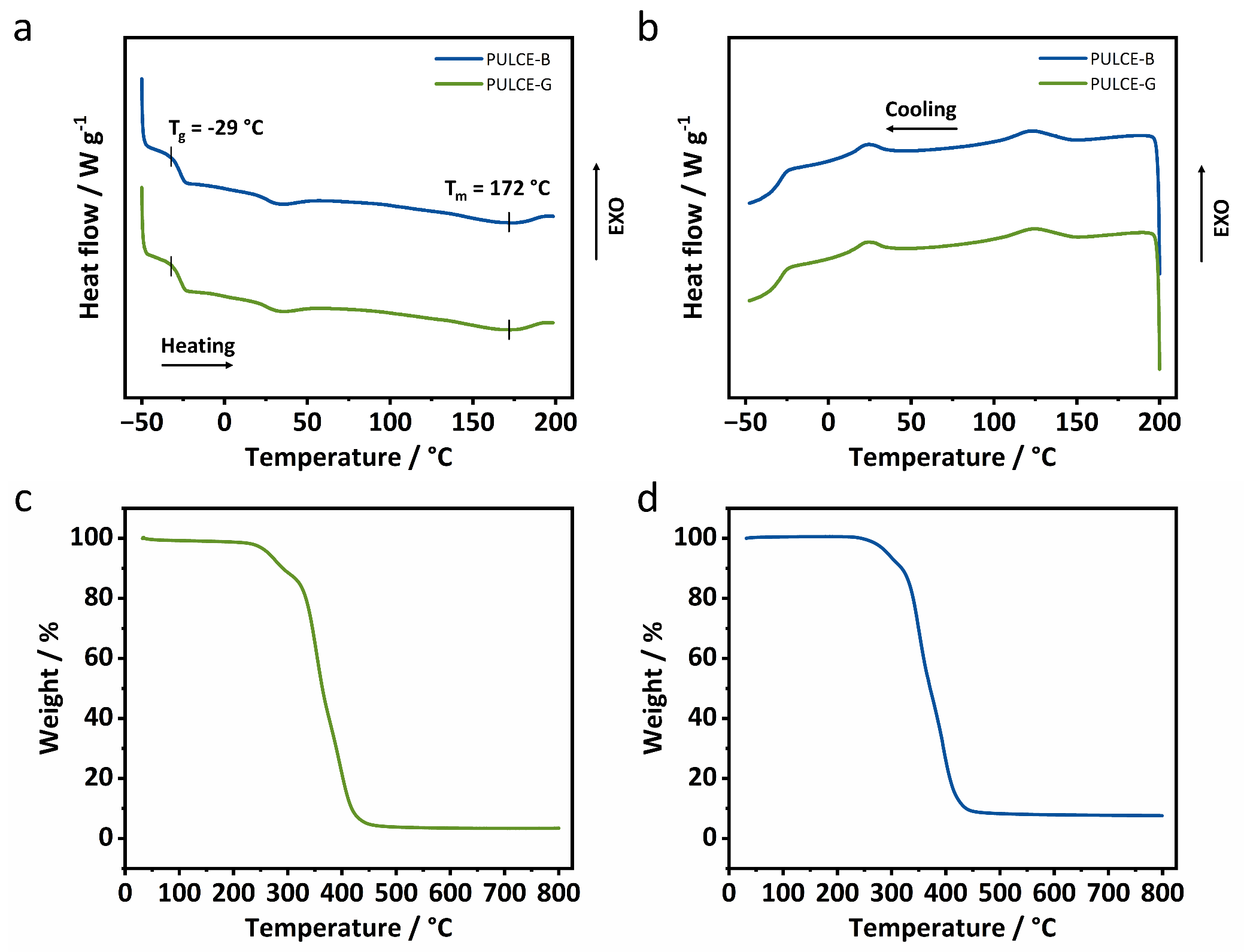


**Figure S15.** DSC thermograms of PULCE-G and PULCE-B during a) heating and b) cooling between -50 and 200 °C. TGA curves of c) PULCE-G and d) PULCE-B. The introduction of phosphors has negligible influence on either *T*_g_ or *T*_m_, indicating excellent compatibility with the polymeric matrix.


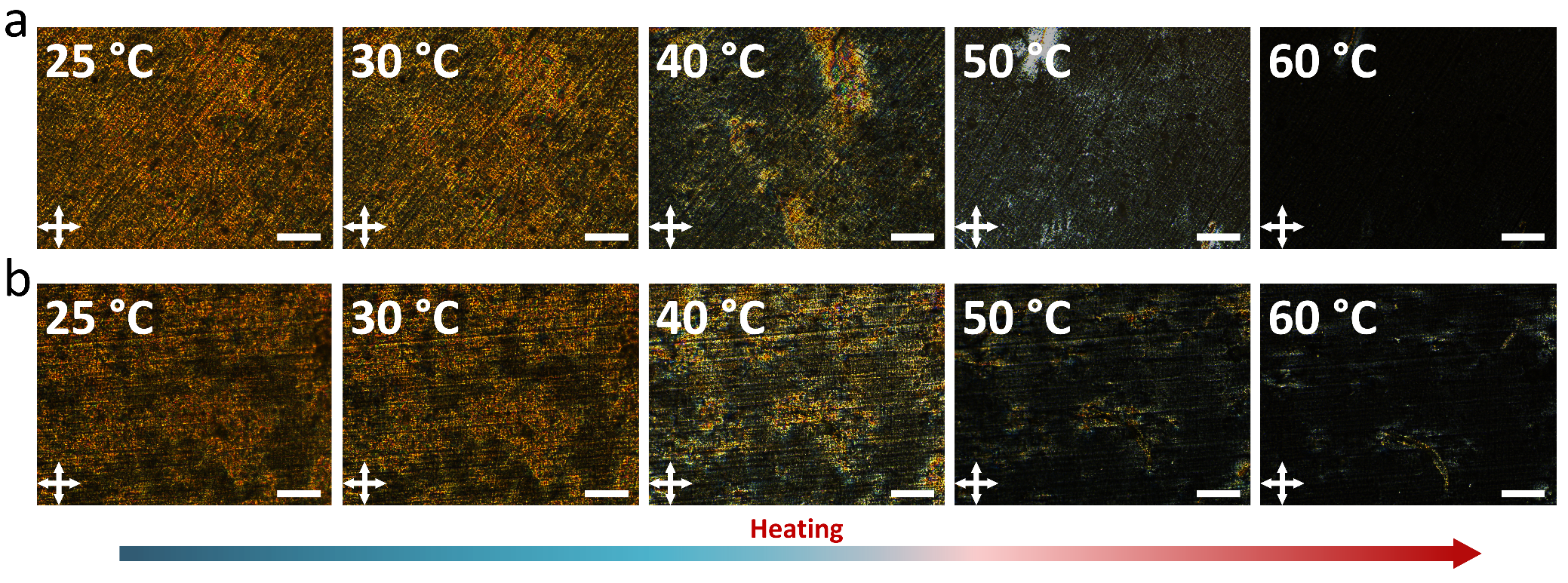


**Figure S16.** POM images of a) PULCE-G and b) PULCE-B films during heating, both exhibiting an obvious disappearance of birefringence at around 60 °C (scale bars = 75 µm).


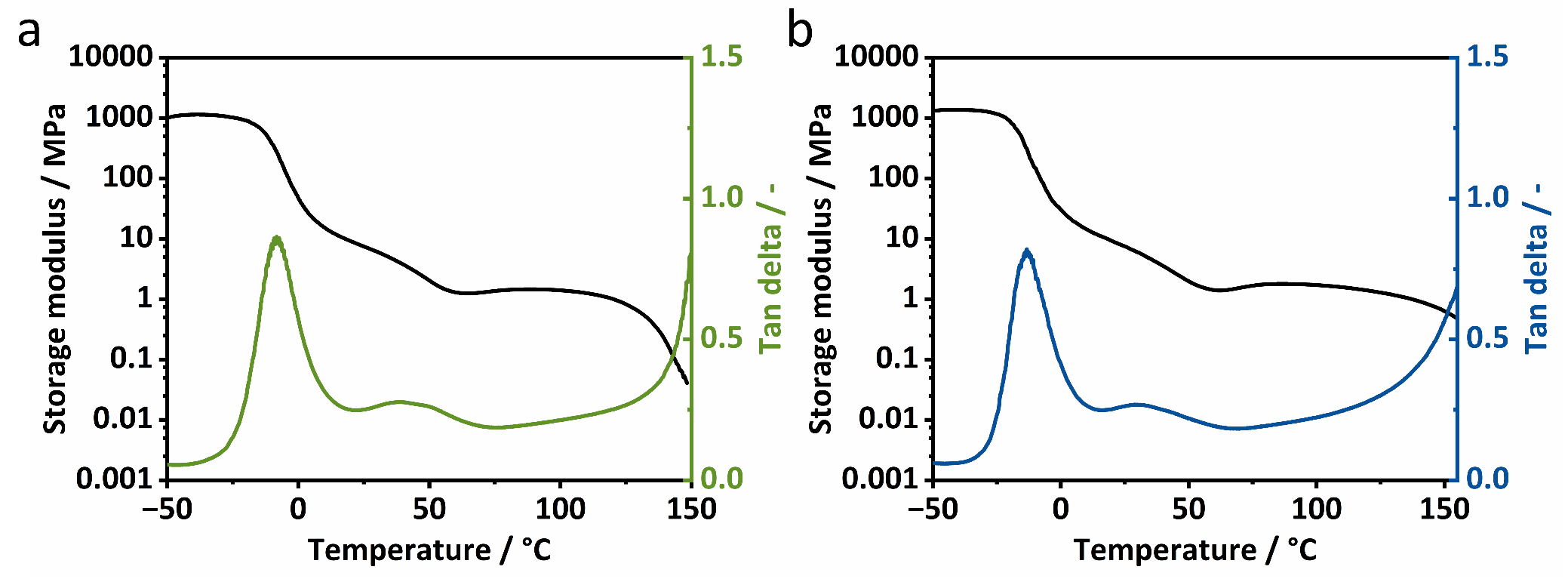


**Figure S17.** DMA curves of a) PULCE-G and b) PULCE-B films, showing an unchanged dynamic hydrogen bonding behavior after phosphor incorporation.


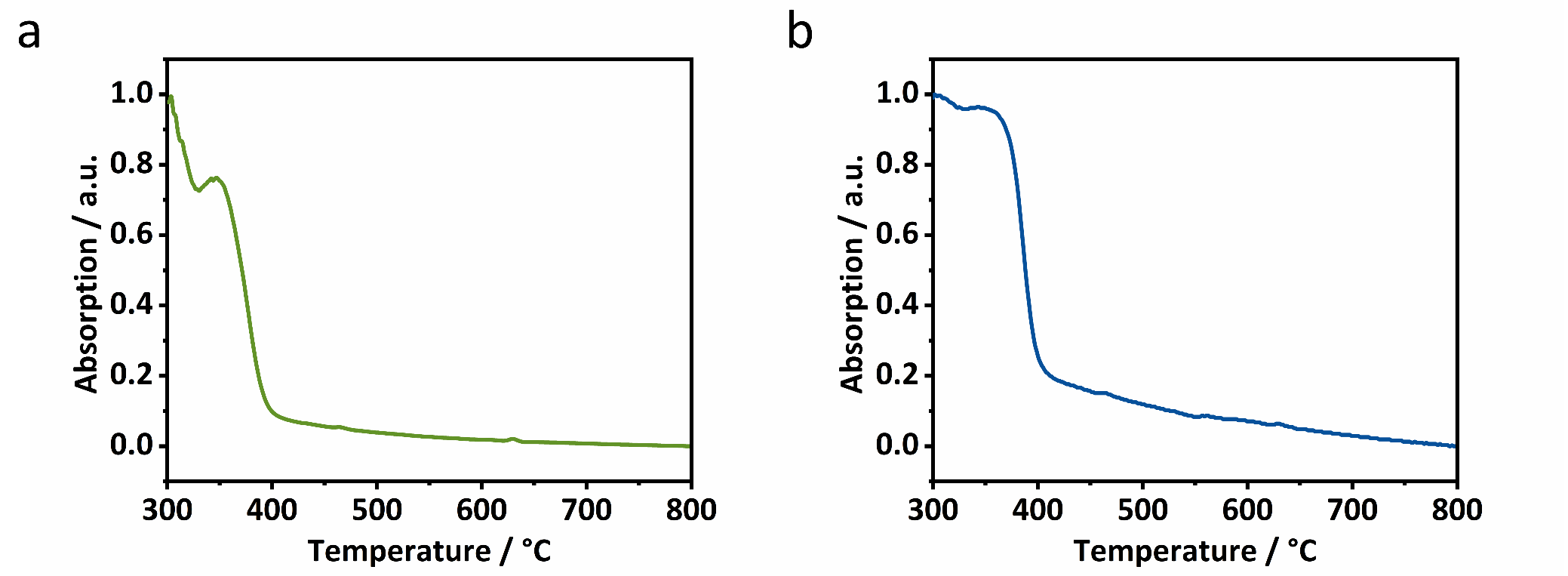


**Figure S18.** UV-Vis absorption spectra of a) PULCE-G and b) PULCE-B films. Both films exhibit broad absorption across the visible region with a distinct peak around 348 nm attributed to the UV absorber.


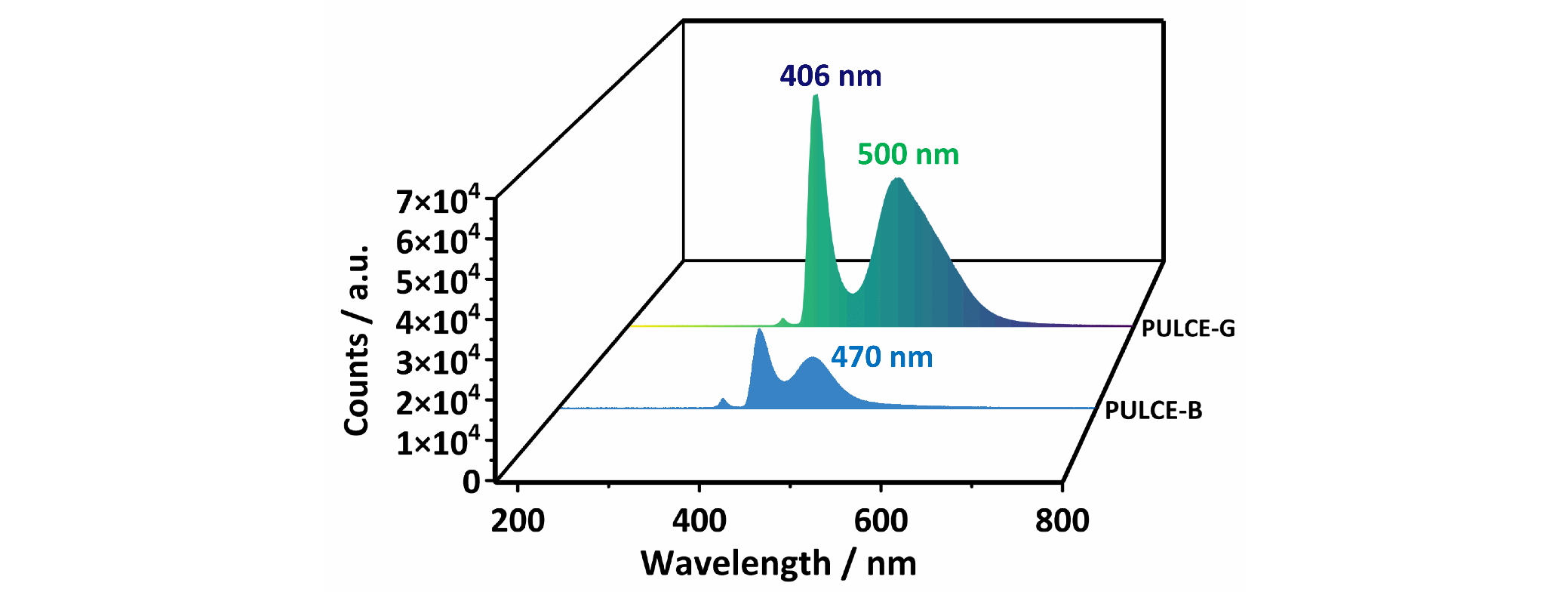


**Figure S19.** Fluorescence spectra of PULCE-G and PULCE-B films under UV excitation.


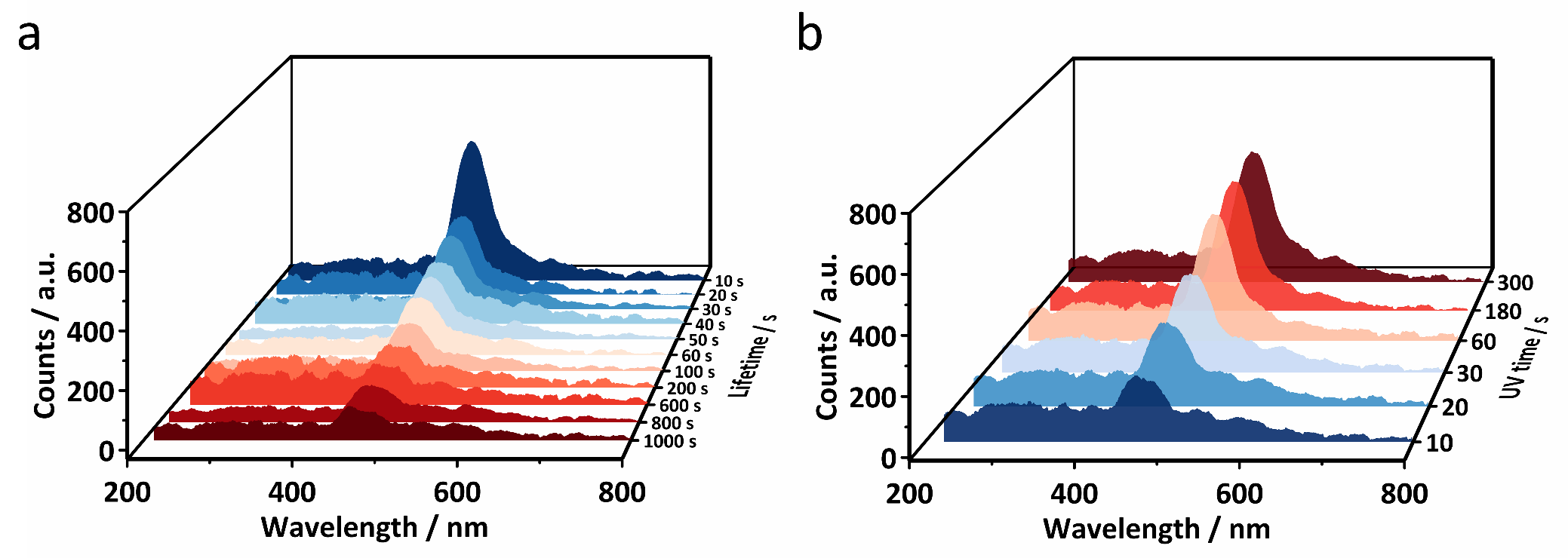


**Figure S20.** a) Phosphorescence decay curves of a PULCE-B film after UV illumination. b) Initial emission intensity of a PULCE-B film under different UV exposure times.


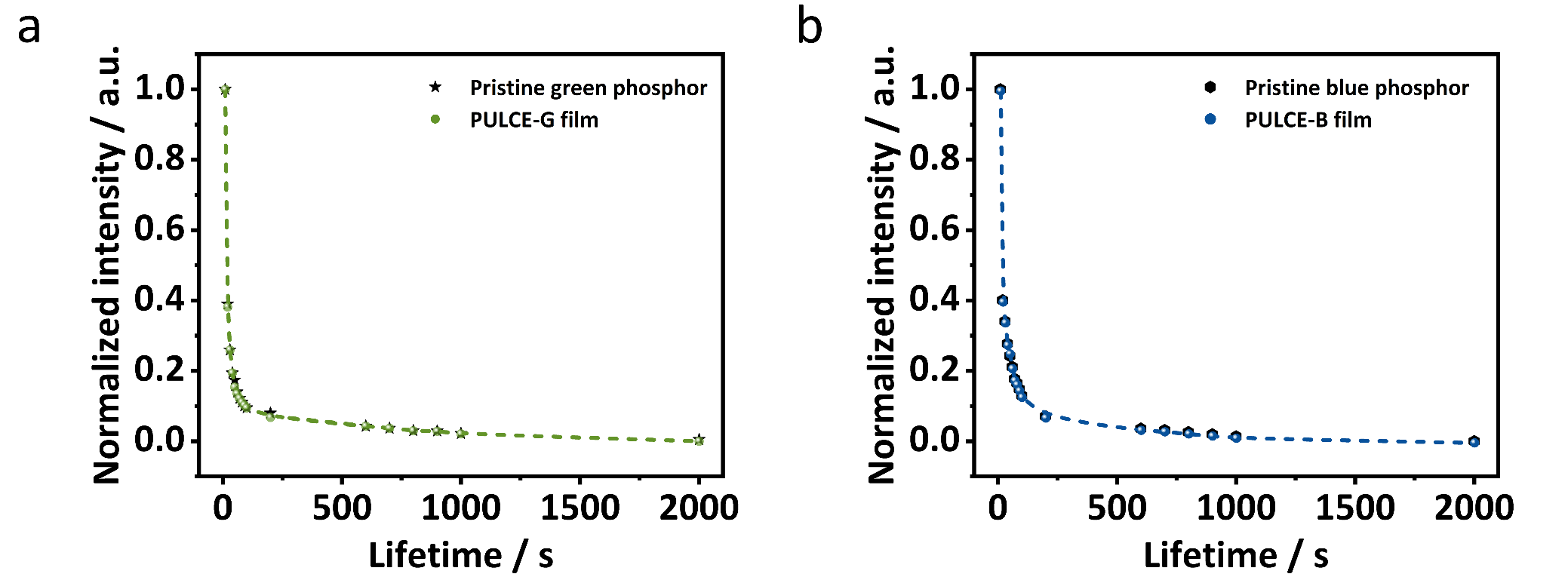


**Figure S21.** Normalized phosphorescence decay curves of a) pristine green phosphors and corresponding PULCE-G film and b) pristine blue phosphors and corresponding PULCE-B film.


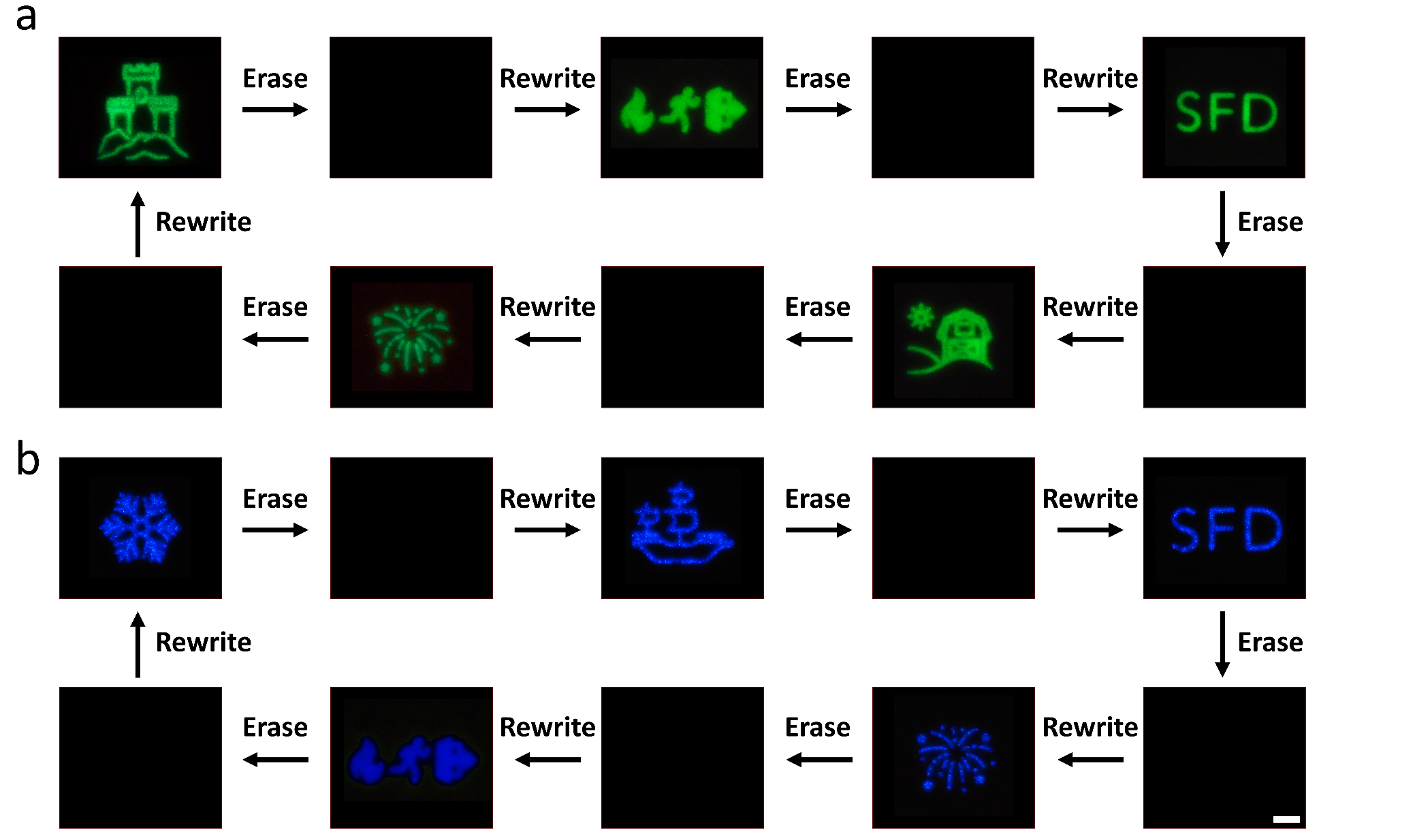


**Figure S22.** Rewritable information storage and erasure in a) a PULCE-G and b) a PULCE-B film using a digital micromirror device (DMD) (scale bar = 5 mm).


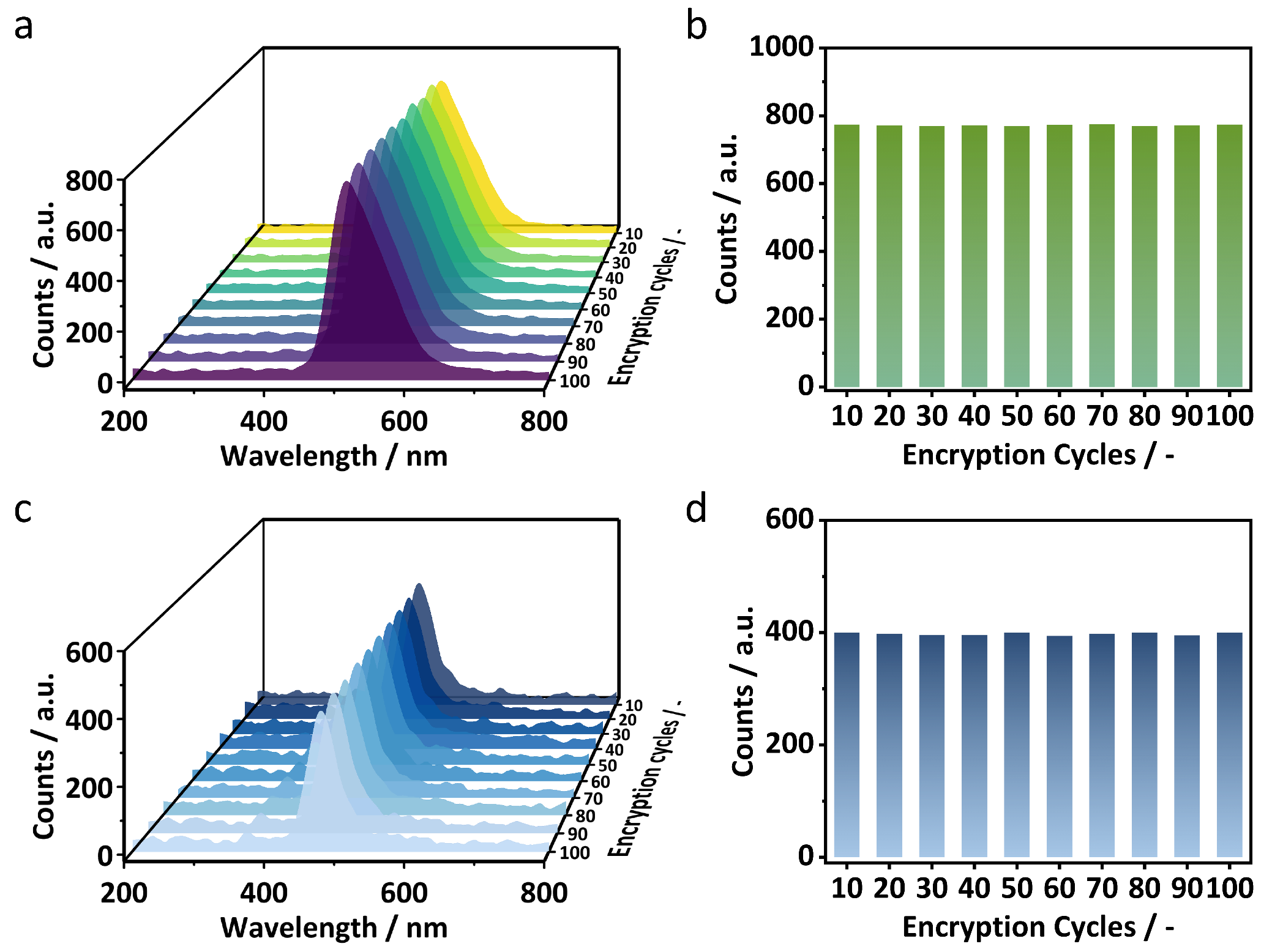


**Figure S23.** Normalized phosphorescence spectra of a) PULCE-G film and c) PULCE-B film collected every 10 encryption-erasing cycles up to 100 cycles. Normalized phosphorescence peak intensity of b) PULCE-G film and d) PULCE-B film, showing stable emission through multiple encryption-erasing cycles.


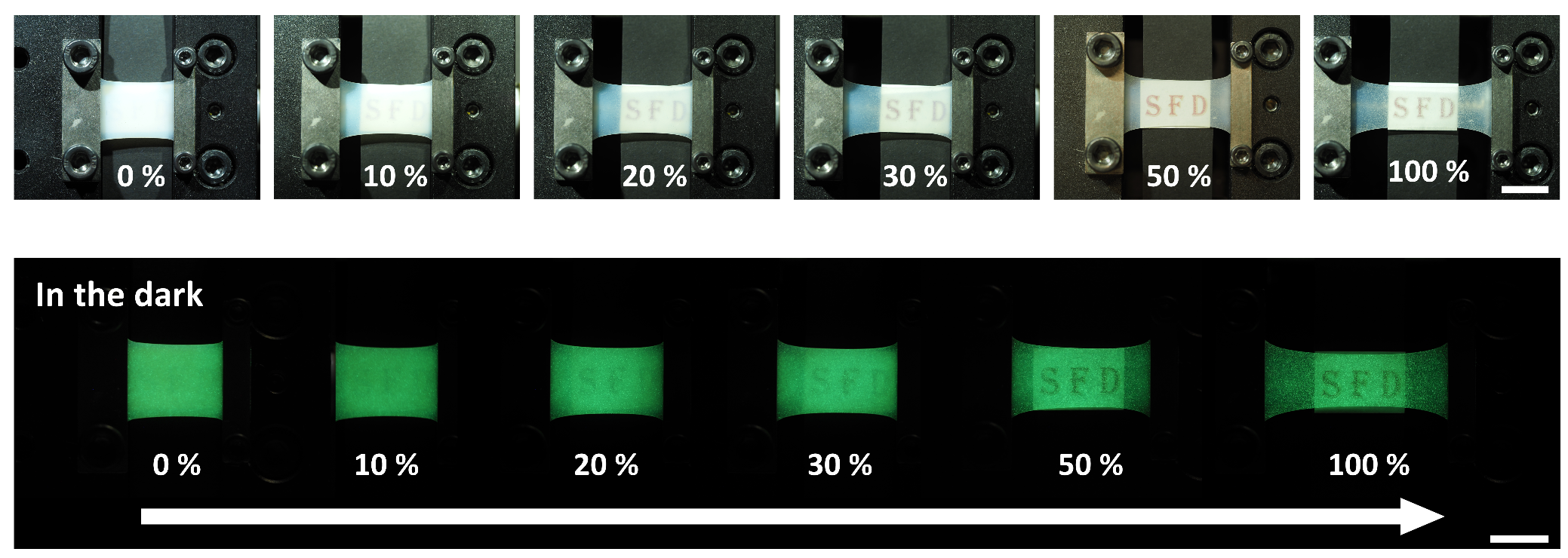


**Figure S24.** Photographs of a polydomain PULCE-G film recorded at various stretches from 0% to 100% under daylight (top) and in the dark (bottom). Printed characters "SFD" on a white background were placed behind the film which gradually become visible with increasing strain (scale bars = 1 cm).


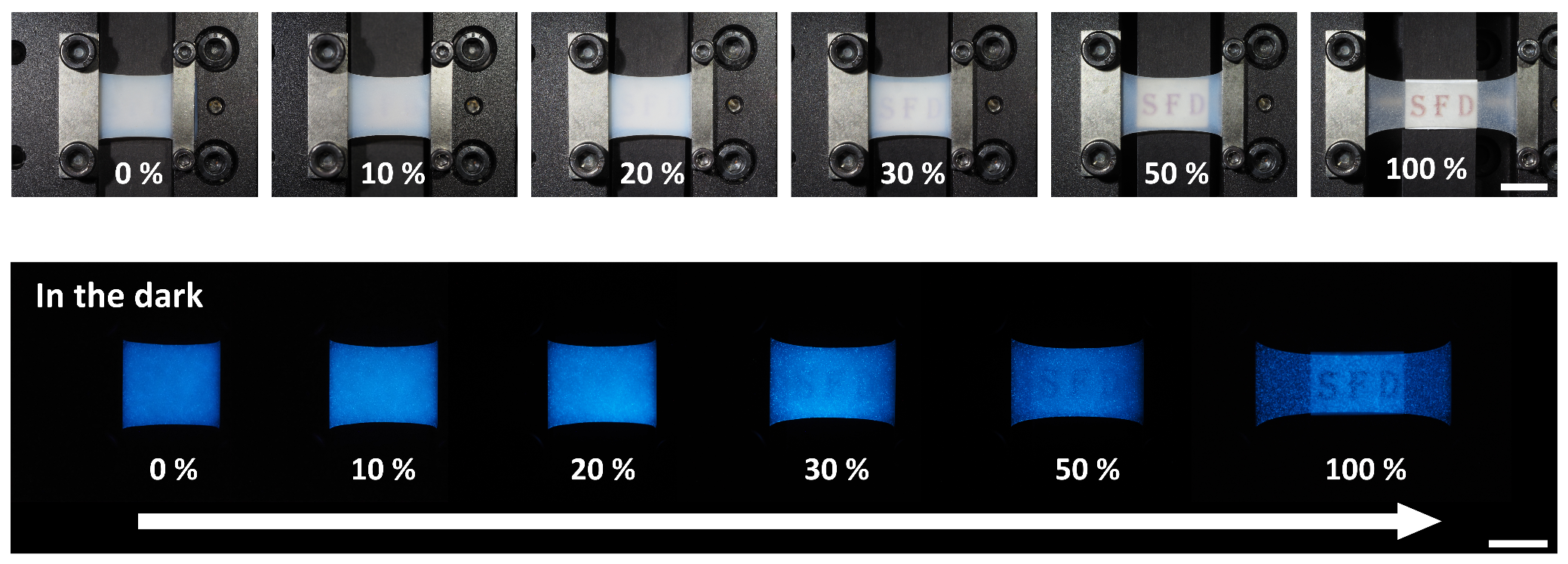


**Figure S25.** Photographs of a polydomain PULCE-B film recorded at various stretches from 0% to 100% under daylight (top) and in the dark (bottom). Printed characters "SFD" on a white background were placed behind the film which gradually become visible with increasing strain (scale bars = 1 cm).


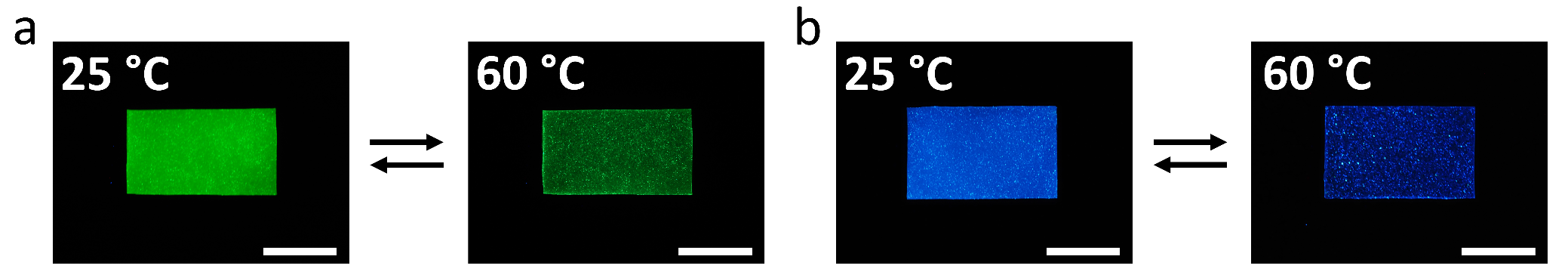


**Figure S26.** Photographs of a) a polydomain PULCE-G film and b) a polydomain PULCE-B film at 25°C and 60 °C after UV illumination in the dark (scale bars = 1 cm).


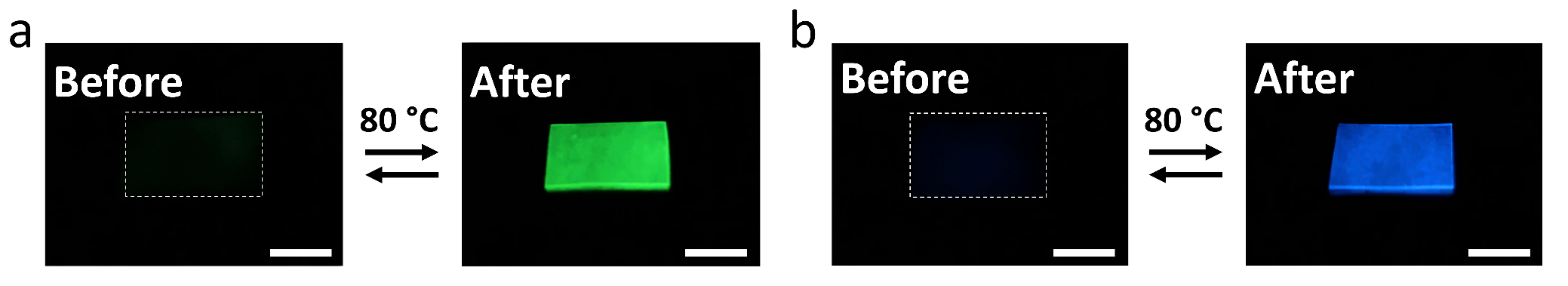


**Figure S27.** Photographs of a) a dimly glowing polydomain PULCE-G film and b) a dimly glowing polydomain PULCE-B film recorded before and immediately after being placed on an 80 °C hot stage (scale bars = 1 cm). Both films exhibited a significant increase in emission brightness upon rapid heating, followed by gradual phosphorescence decay (scale bars = 1 cm).

**
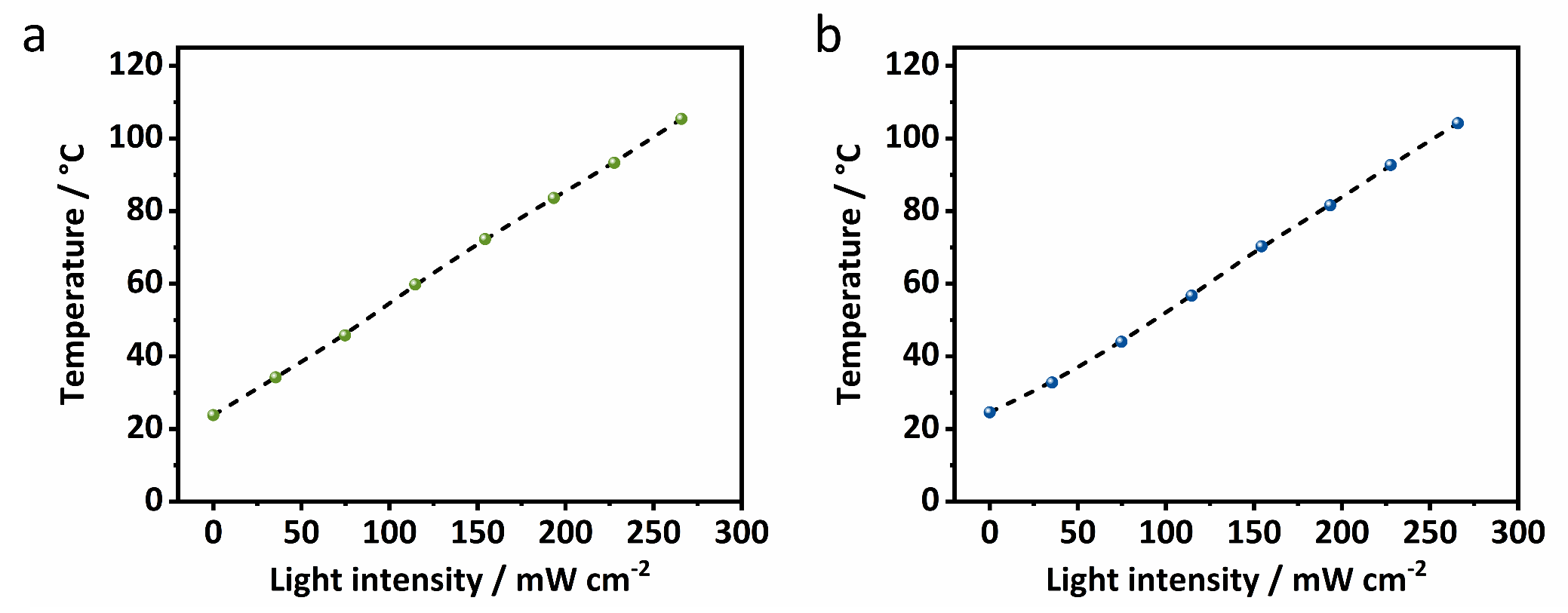
**

**Figure S28.** Measured temperature of a) a PULCE-G film and b) a PULCE-B film exposed to different intensities of UV light for 30 s.


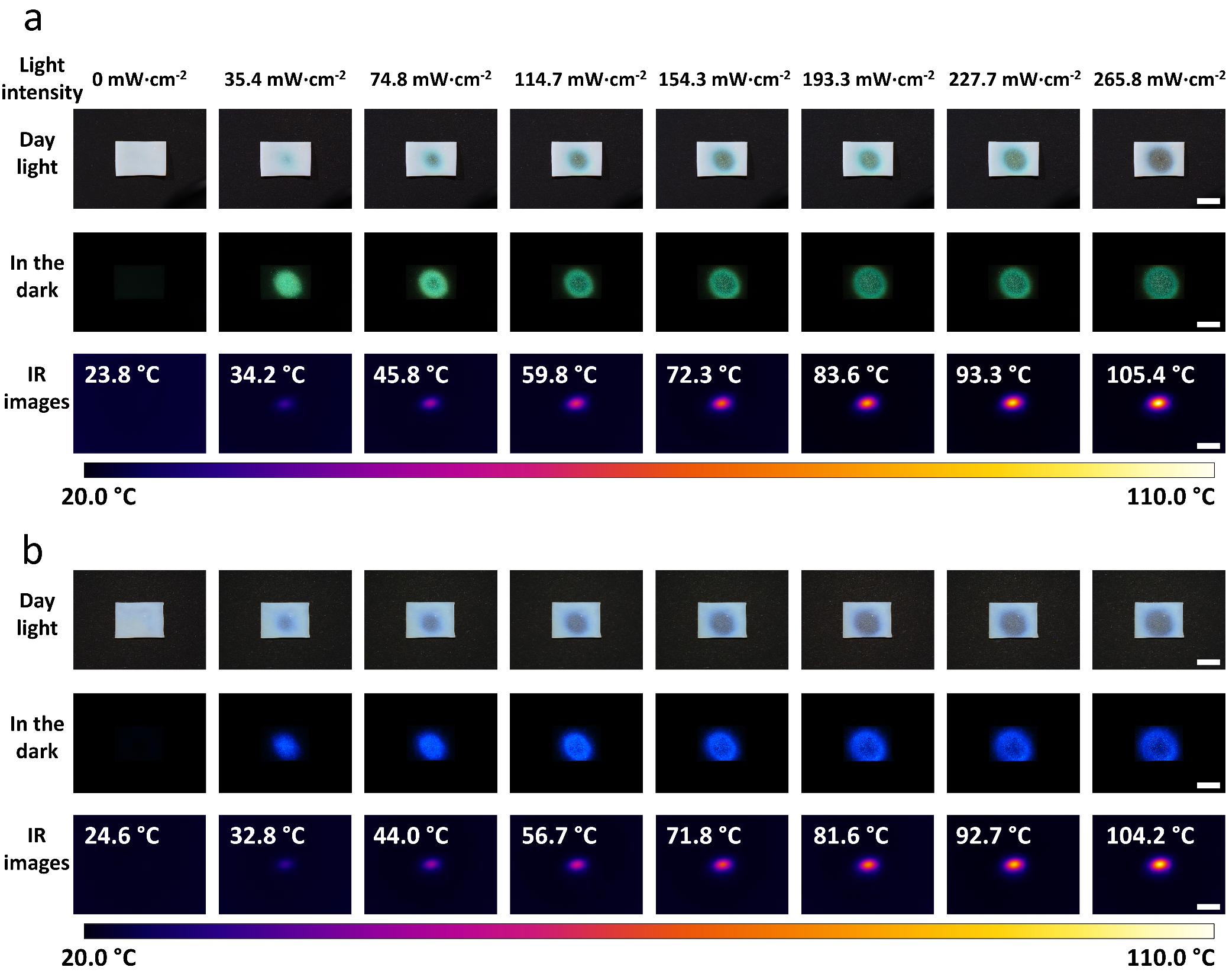


**Figure S29.** a) Photographs under daylight (top), in the dark (middle), and corresponding infrared images (bottom) of a PULCE-G film exposed to UV light at varying intensities. b) Photographs under daylight (top), in the dark (middle), and corresponding infrared images (bottom) of a PULCE-B film exposed to UV light at varying intensities. Temperature of the irradiated region increased due to photothermal conversion (scale bars = 1 cm).

**
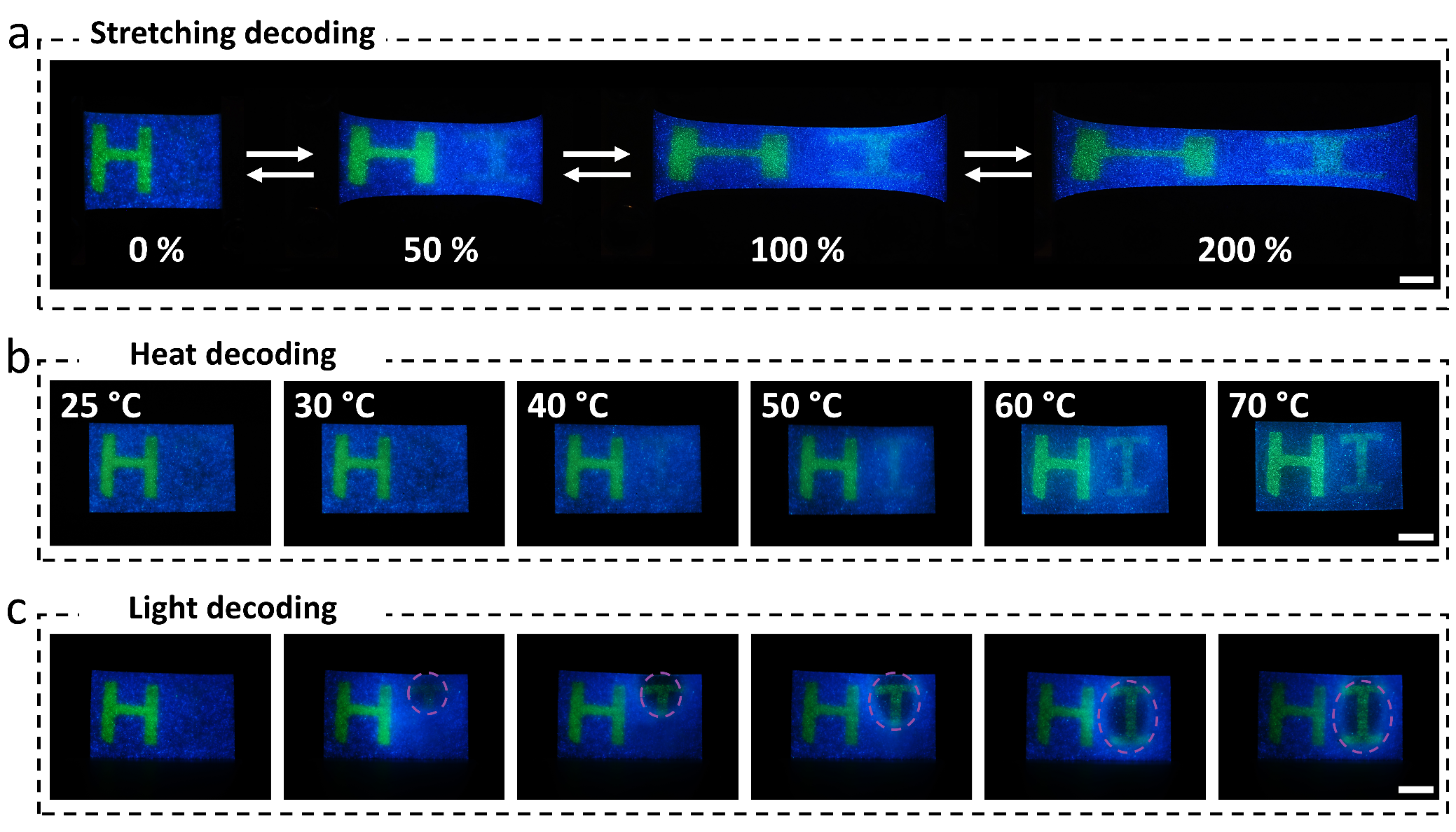
**

**Figure S30.** Demonstration of three distinct decoding strategies for revealing hidden information. a) Mechanical stretching induced decoding: uniaxial elongation (0-200%) makes “I” gradually visible in the dark. b) Thermally induced decoding: gradual uniform heating from 25 to 70 °C transitions the film from a scattering to a transparent state, making “I” visible in the dark. c) Photothermally induced decoding: localized UV irradiation increases the temperature of illuminated area, revealing the hidden “I” in the dark through localized photo-induced transparency change. (scale bars = 5 mm). The violet dashed circle indicates the region locally irradiated with UV light.

**
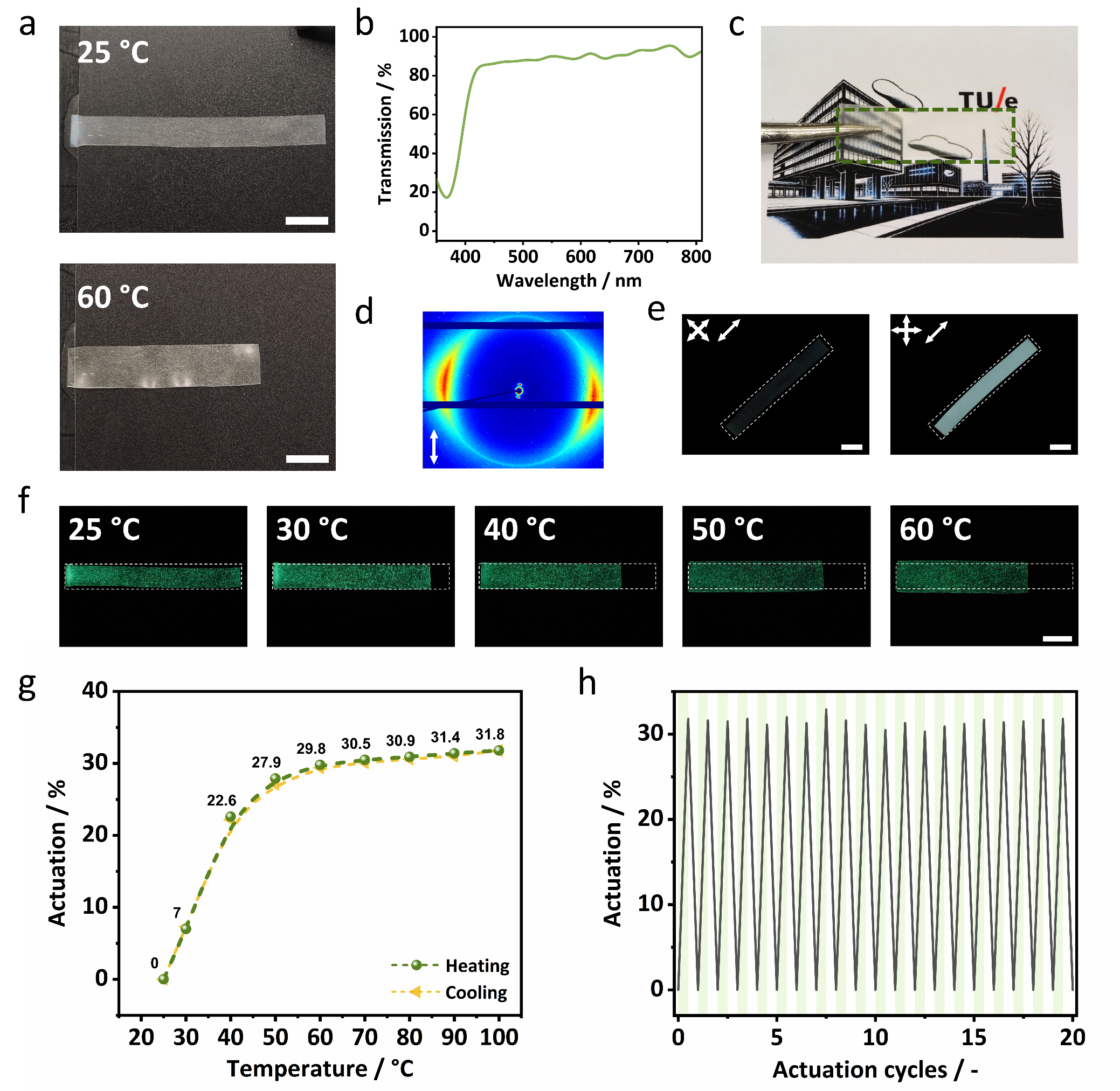
**

**Figure S31.** a) Photographs of a phosphorescent PULCE-G film actuator at 25 °C and 60 °C under daylight, showing contraction along the alignment direction and expansion perpendicular to it. b) UV-Vis transmission spectrum of a PULCE-G film, demonstrating a transmittance of approximately 90% in the visible range. c) Photograph of a PULCE-G film placed over a patterned background. d) 2D-WAXS pattern showing diffraction signal orthogonal to the alignment direction, confirming the uniaxial alignment. e) POM images showing brightness change upon 45° rotation under crossed polarizers, further confirming the uniaxial orientation. f) Photographs of the phosphorescent PULCE-G film actuator during heating (from 25 °C to 60 °C), exhibiting both emission and actuation in the dark. g) Thermal actuation of the phosphorescent PULCE-G film actuator during heating and cooling between 25 °C and 100 °C. h) Reversible deformation over 20 heating-cooling cycles (scale bars = 1 cm).

**
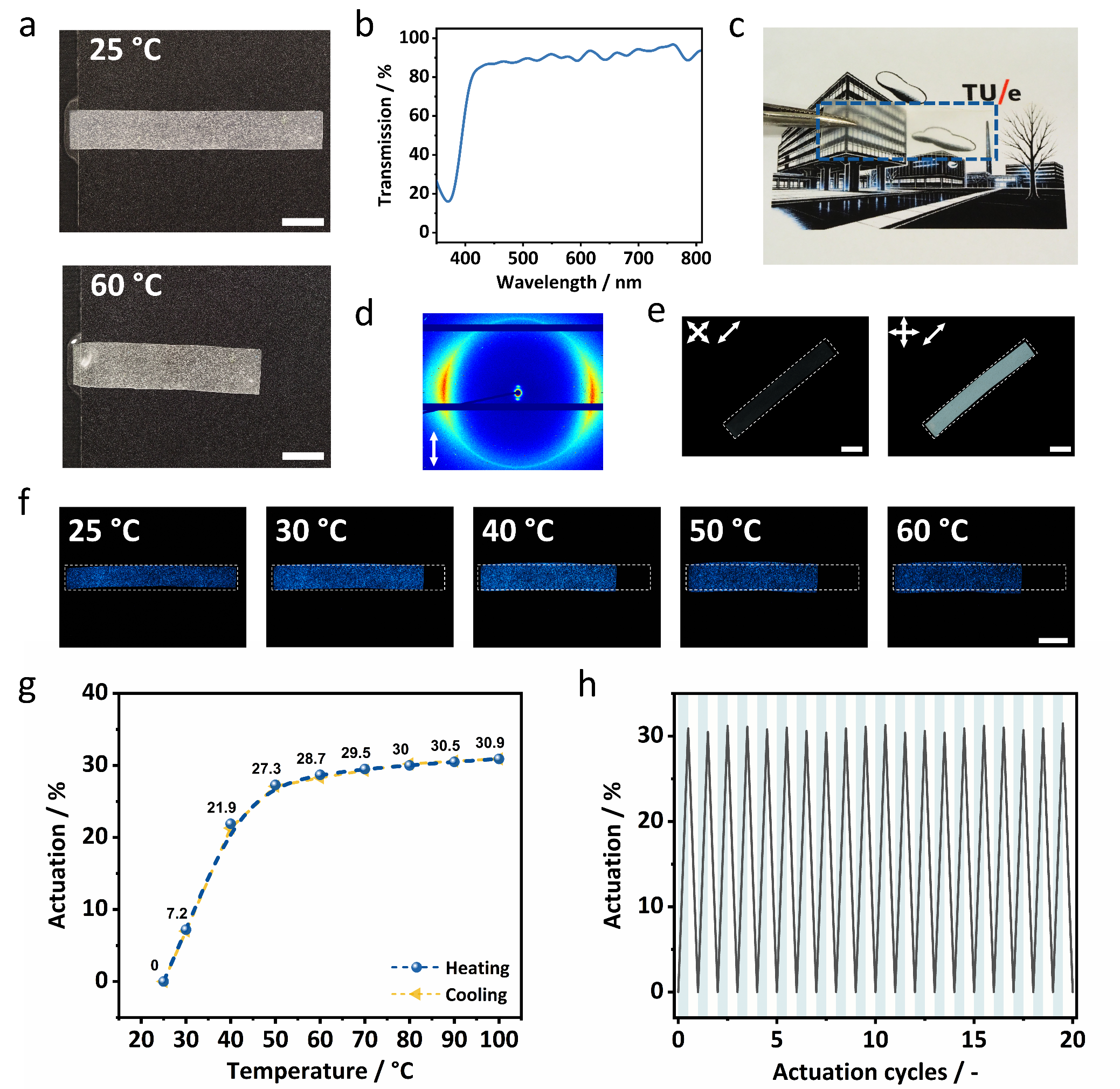
**

**Figure S32.** a) Photographs of a phosphorescent PULCE-B film actuator at 25 °C and 60 °C under daylight, showing contraction along the alignment direction and expansion perpendicular to it. b) UV-Vis transmission spectrum of a PULCE- B film, demonstrating a transmittance of approximately 90% in the visible range. c) Photograph of a PULCE- B film placed over a patterned background. d) 2D-WAXS pattern showing diffraction signal orthogonal to the alignment direction, confirming the uniaxial alignment. e) POM images showing brightness change upon 45° rotation under crossed polarizers, further confirming the uniaxial orientation. f) Photographs of the phosphorescent PULCE-B film actuator during heating (from 25 °C to 60 °C), exhibiting both emission and actuation in the dark. g) Thermal actuation of the phosphorescent PULCE-B film actuator during heating and cooling between 25 °C and 100 °C. h) Reversible deformation over 20 heating-cooling cycles (scale bars = 1 cm).


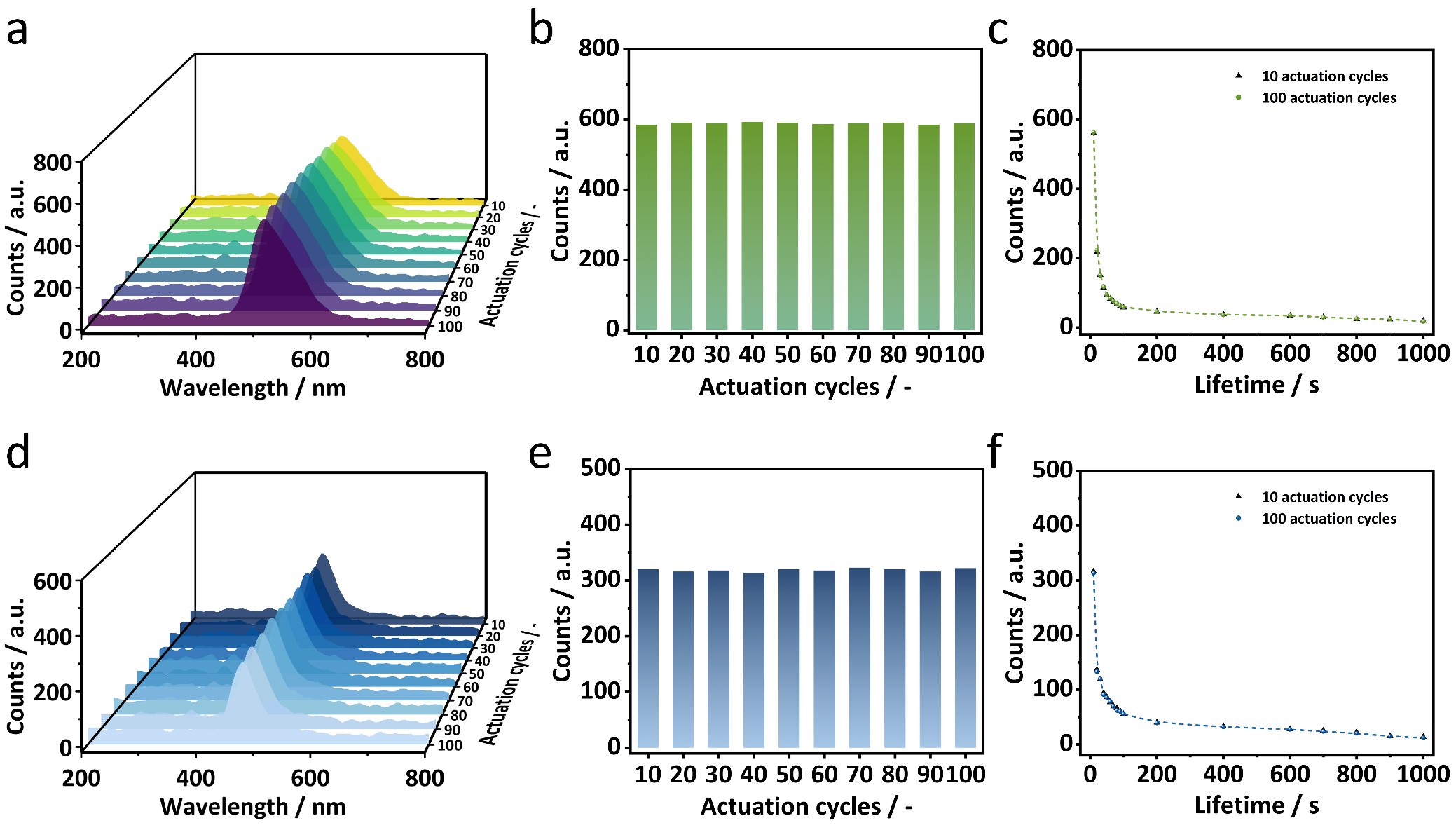


**Figure S33.** Normalized phosphorescence spectra of a) aligned PULCE-G film and d) aligned PULCE-B film recorded every 10 actuation cycles up to 100 cycles. Normalized phosphorescence peak intensity of b) aligned PULCE-G film and e) aligned PULCE-B film, showing stable emission intensity. Phosphorescence decay curves of c) aligned PULCE-G film and f) aligned PULCE-B film after 10 and 100 actuation cycles.

**
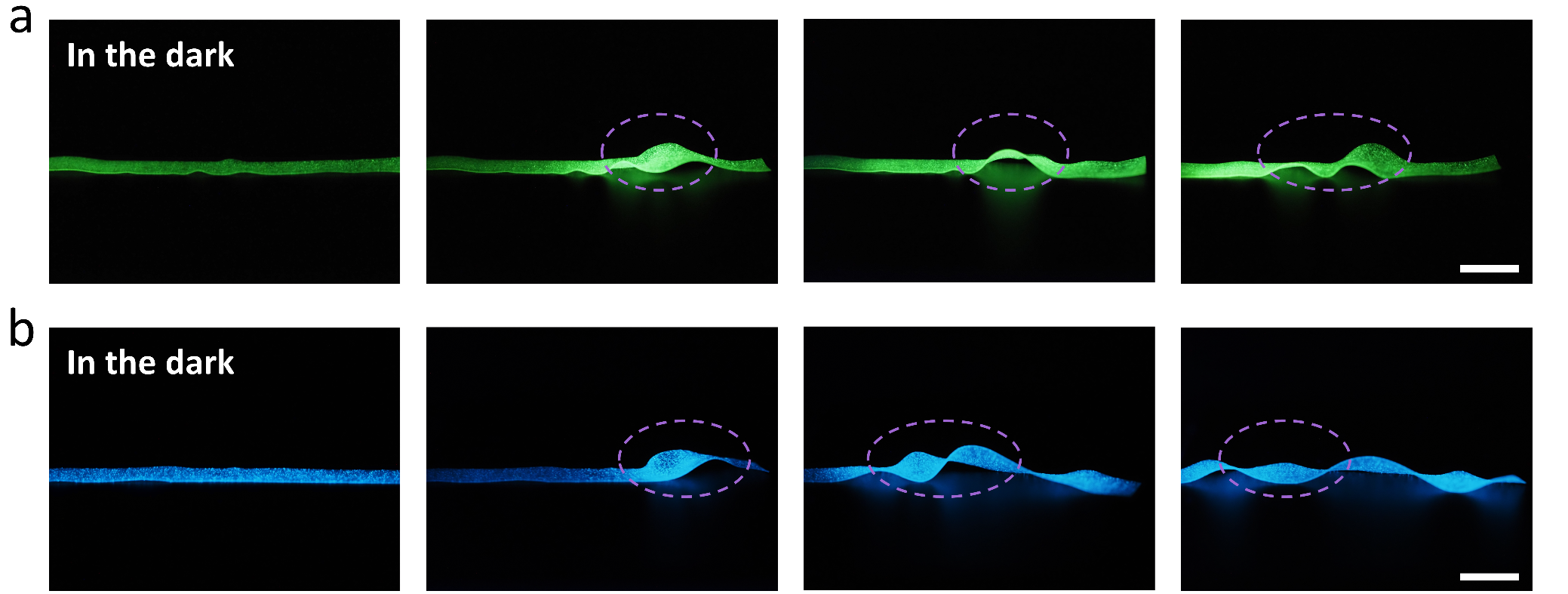
**

**Figure S34.** Photo-response of a) a phosphorescent PULCE-G film actuator and b) a phosphorescent PULCE-B film actuator in the dark (scale bars = 1 cm). The violet dashed circle indicates the region locally irradiated with UV light.


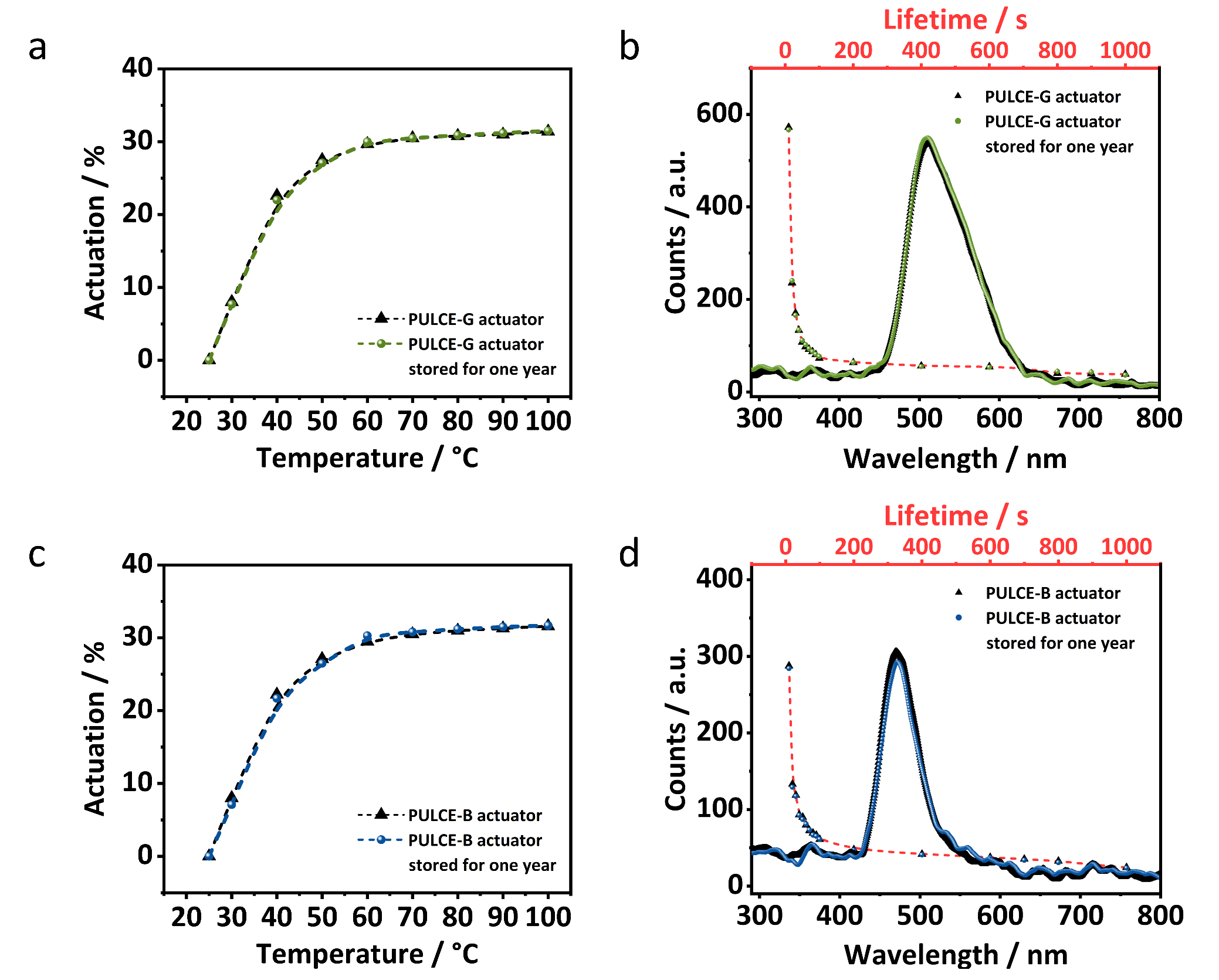


**Figure S35.** Comparison of actuation strain of a) PULCE-G and c) PULCE-B actuators measured before and after one year of storage under ambient conditions. Normalized phosphorescence spectra and corresponding decay profiles of b) PULCE-G and d) PULCE-B actuators measured after fabrication and again after one year of ambient storage.


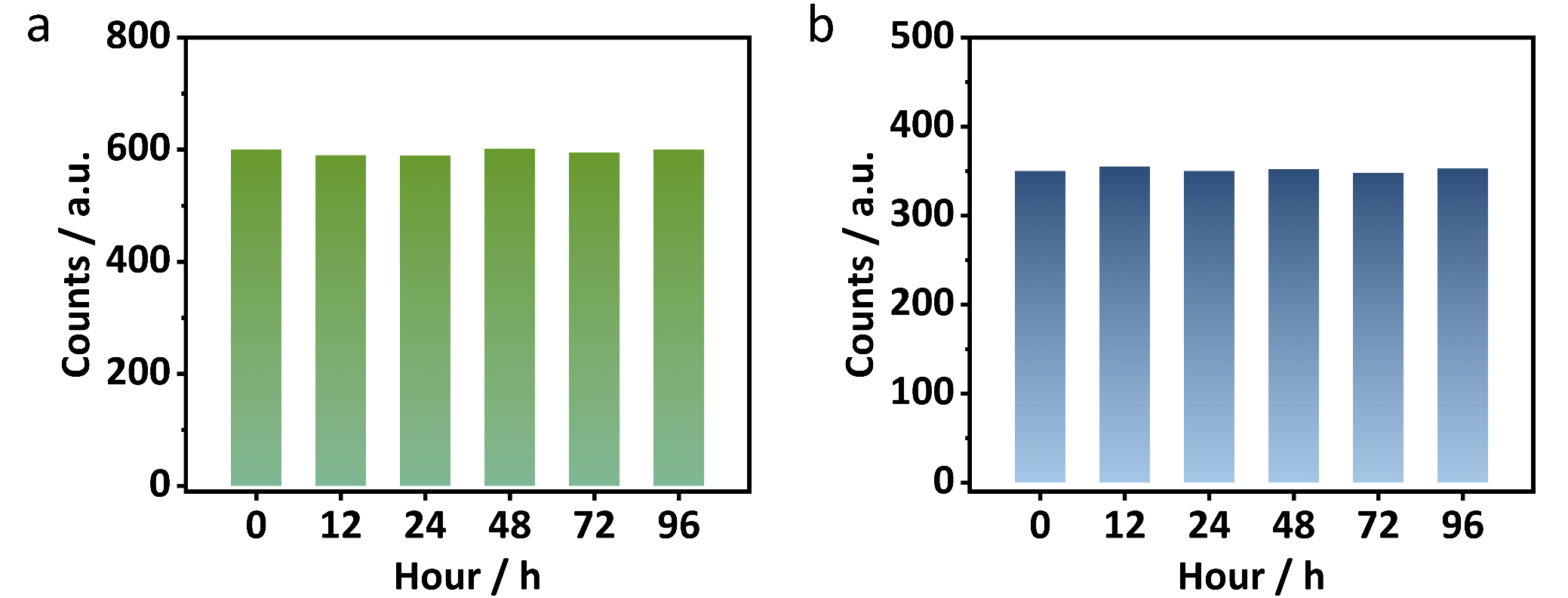


**Figure S36.** Normalized phosphorescence intensity of a) PULCE-G and b) PULCE-B actuators after immersion in water for 12 h, 24 h, 48 h, 72 h, and 96 h.

**
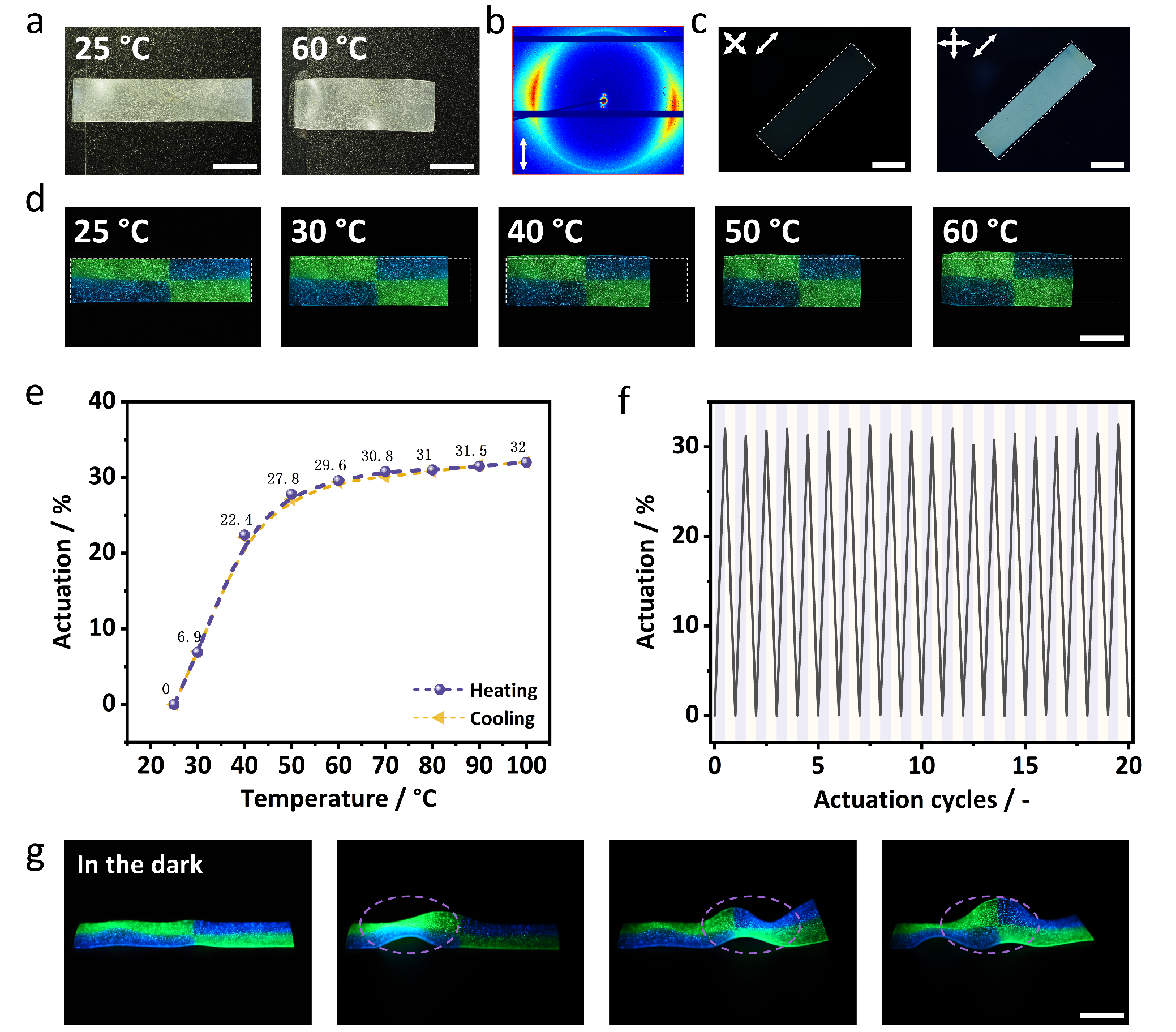
**

**Figure S37.** a) Photographs of the hot-welded encoded, bicolored phosphorescent actuator at 25 °C and 60 °C under daylight, showing contraction along the alignment direction and expansion perpendicular to it. b) 2D-WAXS pattern showing the diffraction signal orthogonal to the alignment direction, confirming the uniaxial alignment. c) POM images showing brightness change upon 45° rotation under crossed polarizers, further confirming the uniaxial orientation. d) Photographs of the encoded bicolored phosphorescent actuator during heating (from 25 °C to 60 °C), exhibiting both emission and actuation in the dark. e) Thermal actuation of the hot-welded actuator during heating and cooling between 25 °C and 100 °C. f) Reversible deformation over 20 heating-cooling cycles. g) Photo-response of the encoded bicolored phosphorescent actuator in the dark (scale bars = 1 cm). The violet dashed circle indicates the region locally irradiated with UV light.

**
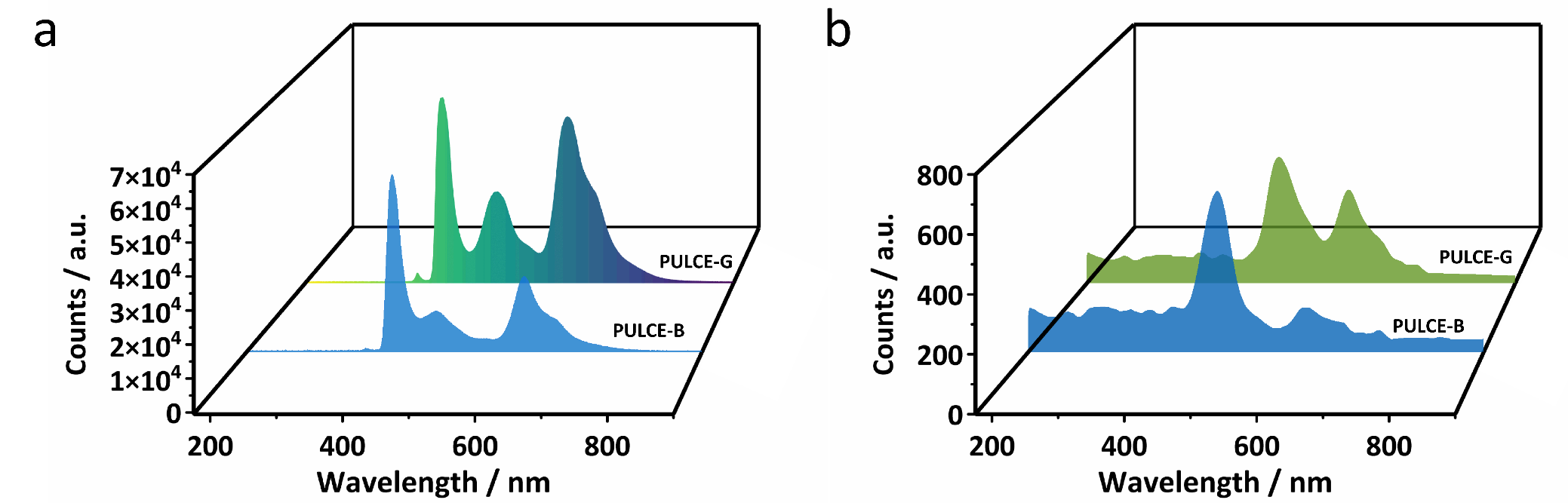
**

**Figure S38.** a) Fluorescence spectra of PULCE-G and PULCE-B films doped with the fluorescent dye Lumogen R305. b) Phosphorescence spectra of PULCE-G and PULCE-B films doped with the fluorescent dye Lumogen R305.

**
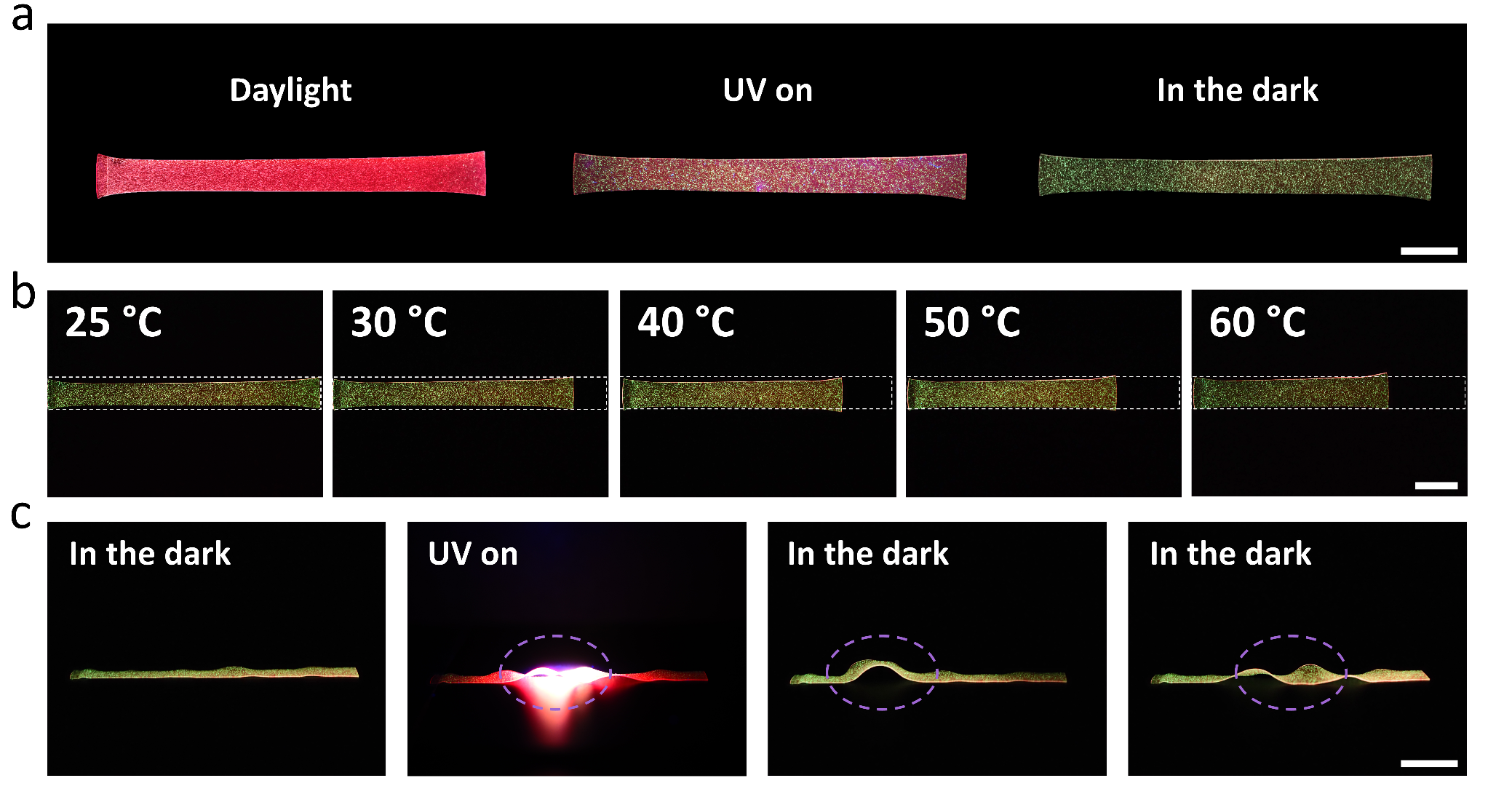
**

**Figure S39.** a) Photographs of a phosphorescent PULCE-G film actuator doped with Lumogen R305 taken under daylight, UV illumination, and in the dark. b) Thermal response of the aligned film measured between 25 °C and 60 °C in the dark, showing both green emission and shape deformation. c) Photo-response of the phosphorescent PULCE-G film actuator doped with Lumogen R305 upon local UV exposure (scale bars = 1 cm). The violet dashed circle indicates the region locally irradiated with UV light.

**
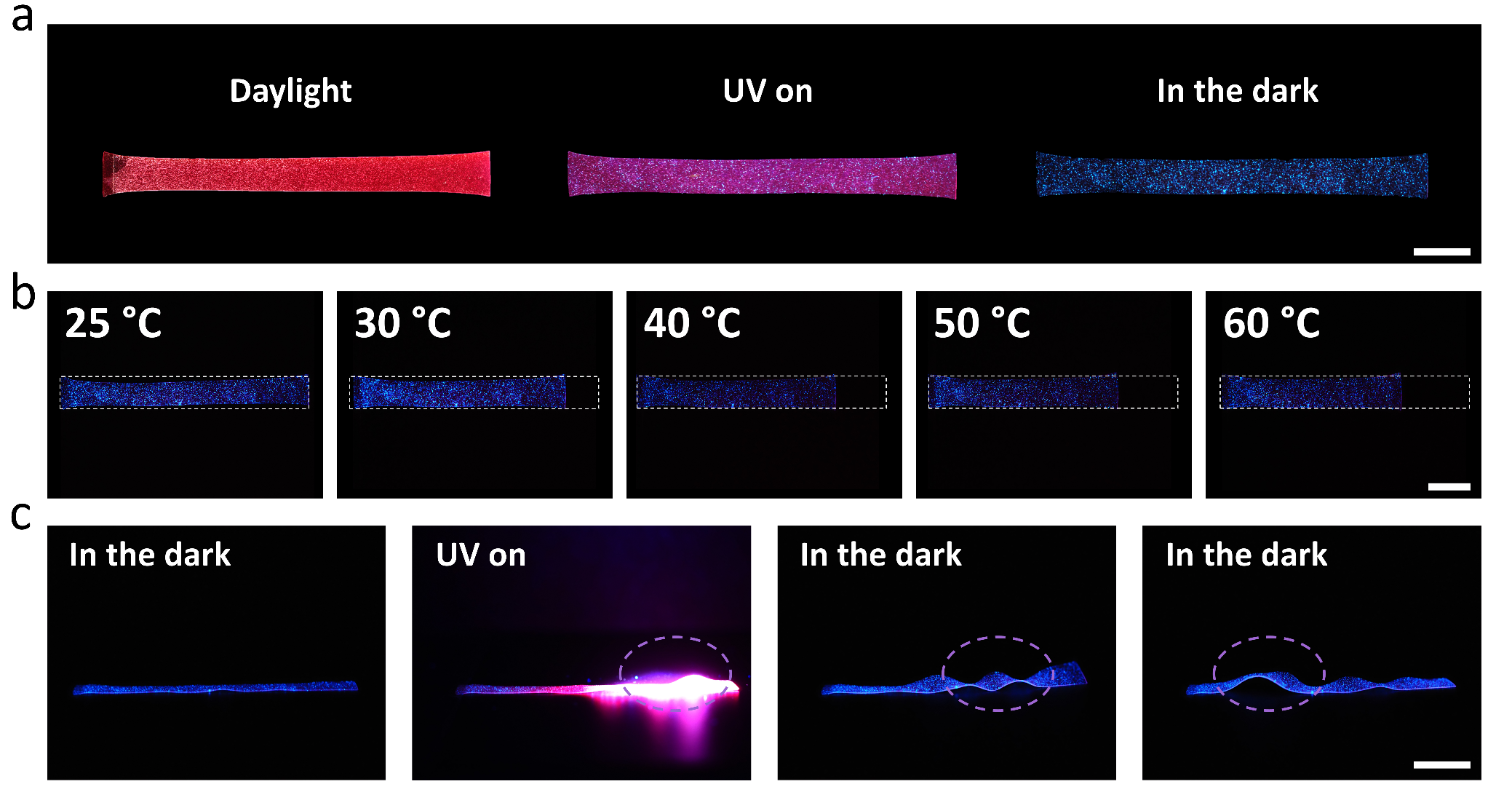
**

**Figure S40.** a) Photographs of a phosphorescent PULCE-B film actuator doped with Lumogen R305, taken under daylight, UV illumination, and in the dark. b) Thermal response of the aligned film measured between 25 °C and 60 °C in the dark, showing both blue emission and shape deformation. c) Photo-response of a phosphorescent PULCE-B film actuator doped with Lumogen R305 upon local UV exposure (scale bars = 1 cm). The violet dashed circle indicates the region locally irradiated with UV light.

**
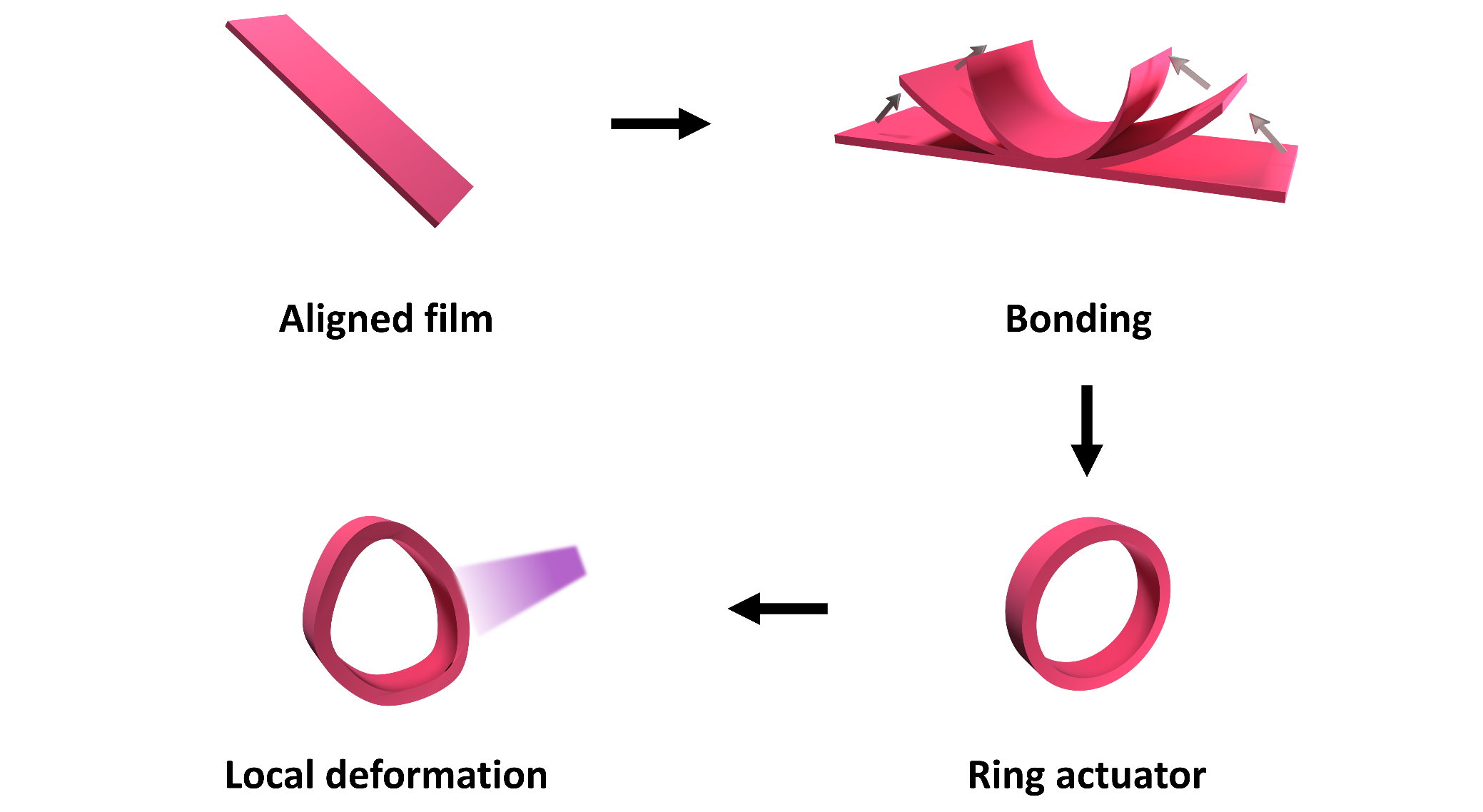
**

**Figure S41.** Schematic illustration of the fabrication of the glowing rolling wheel.

**
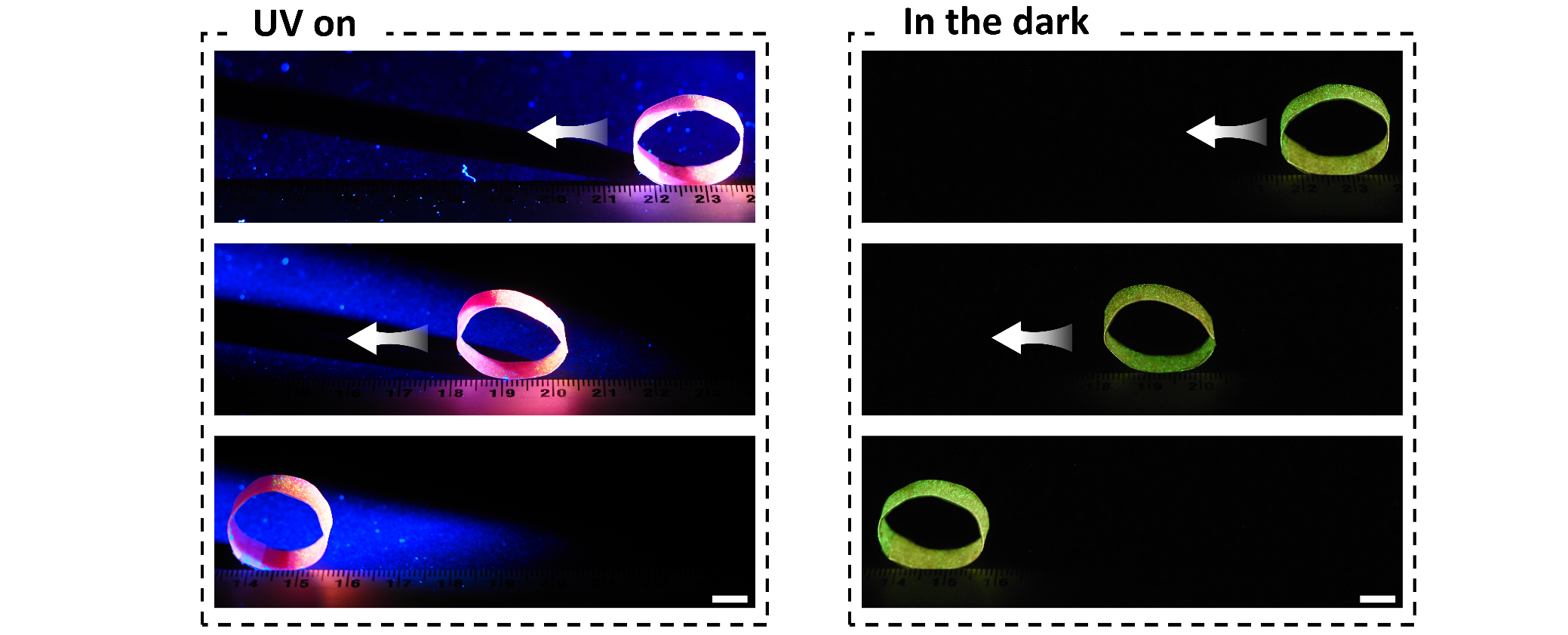
**

**Figure S42.** Photographs of the PULCE-G based ring actuator under UV illumination from the right showing its position (left), and corresponding images in the dark exhibiting its phosphorescent emission before and after movement (right) (scale bars = 5 mm, total time = 30 s).

**
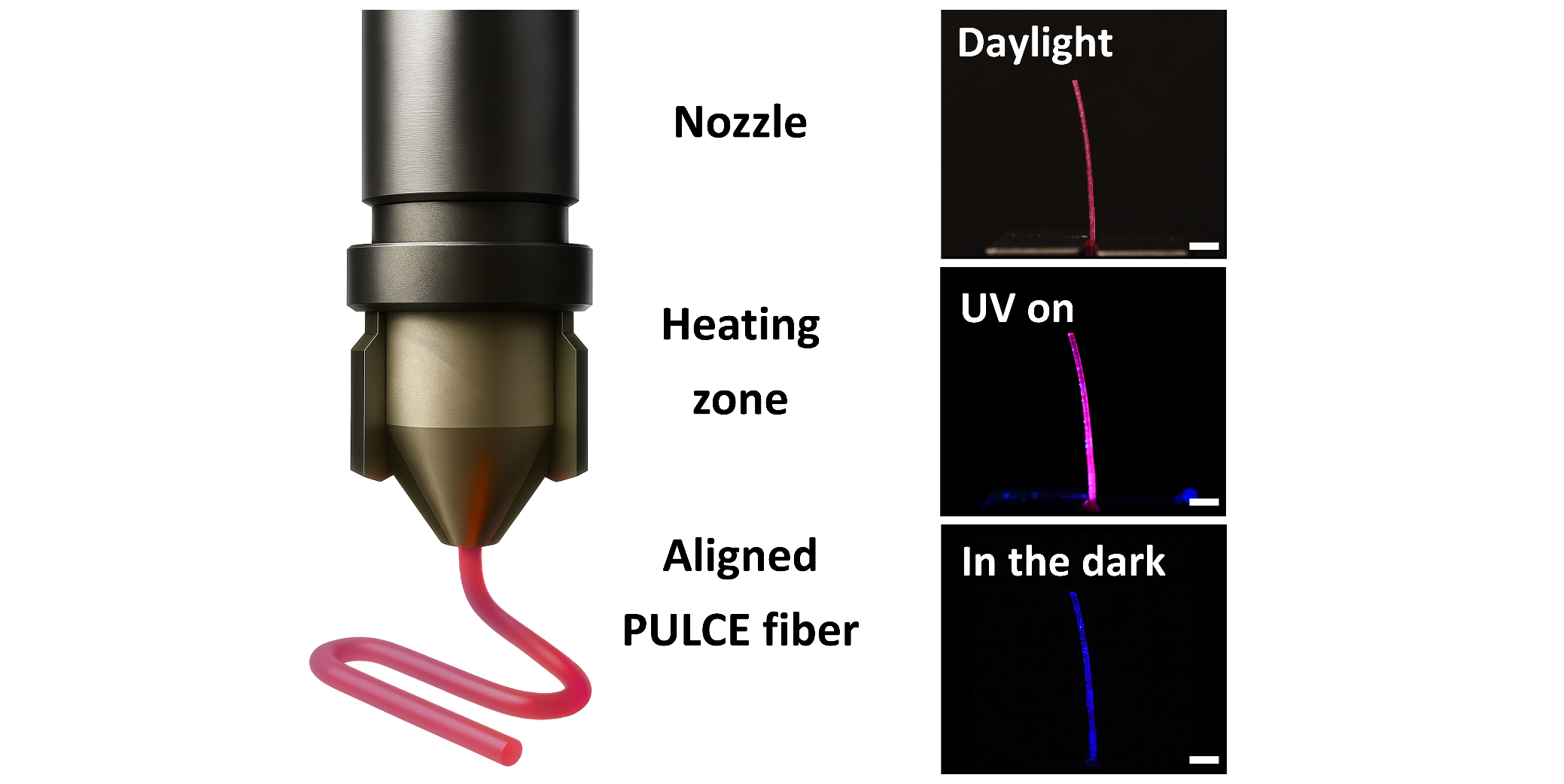
**

**Figure S43.** Schematic of the direct ink writing (DIW) process of phosphorescent PULCE fibers (left) and photographs of the printed fibers under daylight, UV excitation, and in the dark (right) (scale bars = 2 mm).

**
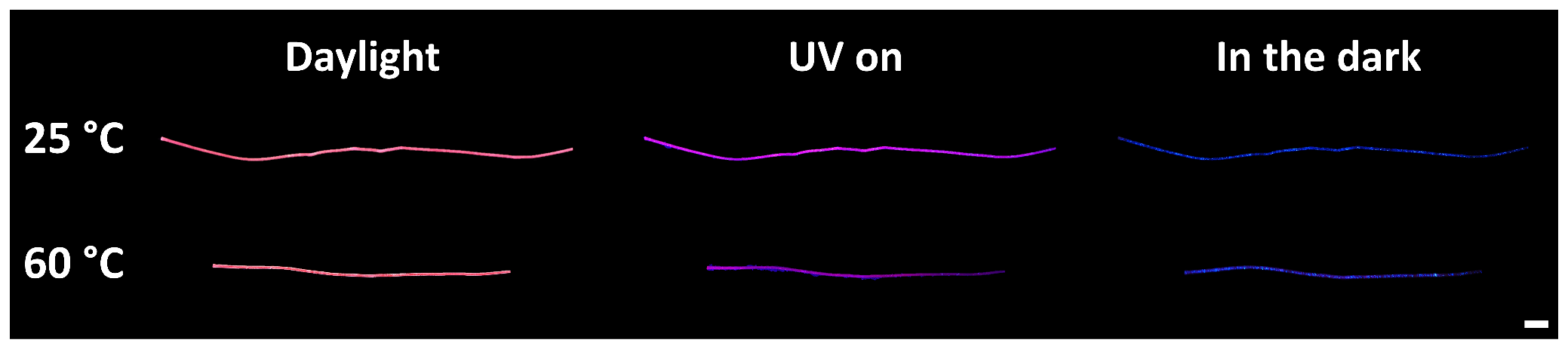
**

**Figure S44.** Thermal response of the printed PULCE fiber under daylight, UV on, and in the dark, exhibiting both blue phosphorescence and ~30% reversible deformation between 25 °C and 60 °C, consistent with film-based thermal actuation behavior (scale bar = 2 mm).

**
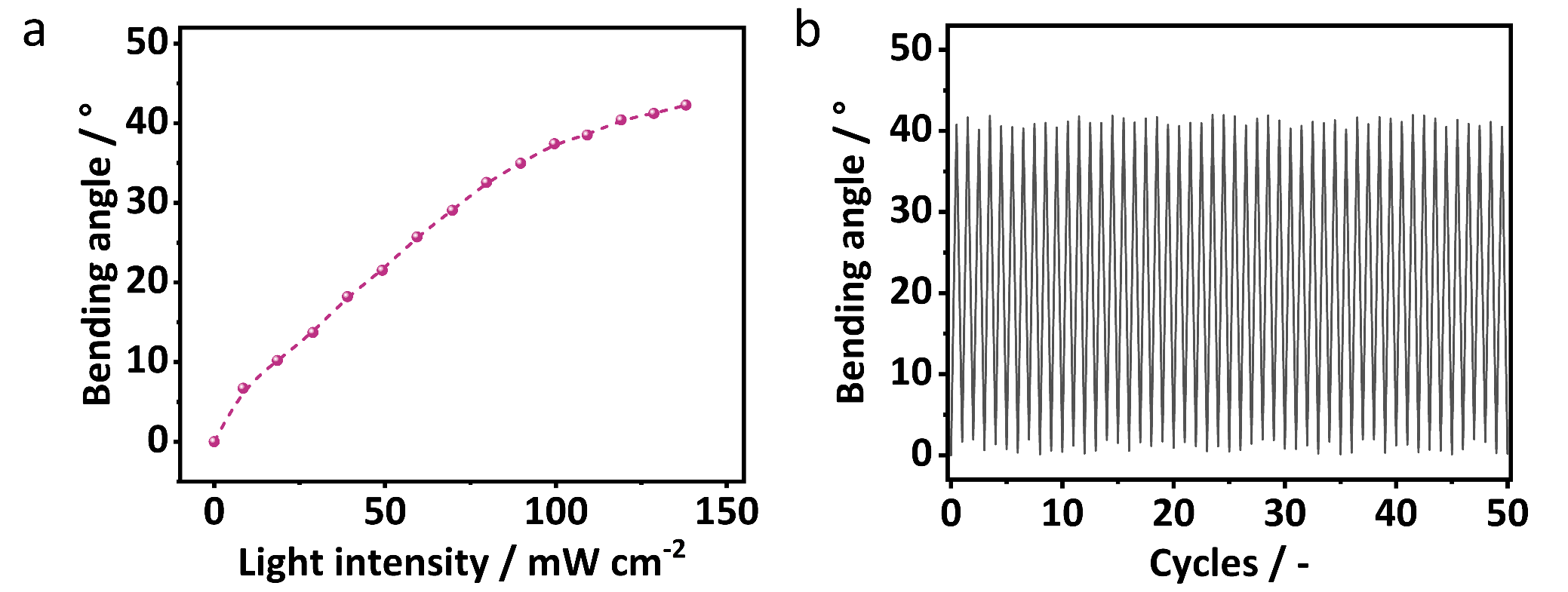
**

**Figure S45.** a) Quantitative bending angle of the luminescent fiber actuator under different UV light intensities, demonstrating a maximum curvature of ~45°. c) Actuation repeatability of the luminescent fiber actuator under 50 photo-induced cycles, showing stable bending behavior.

**
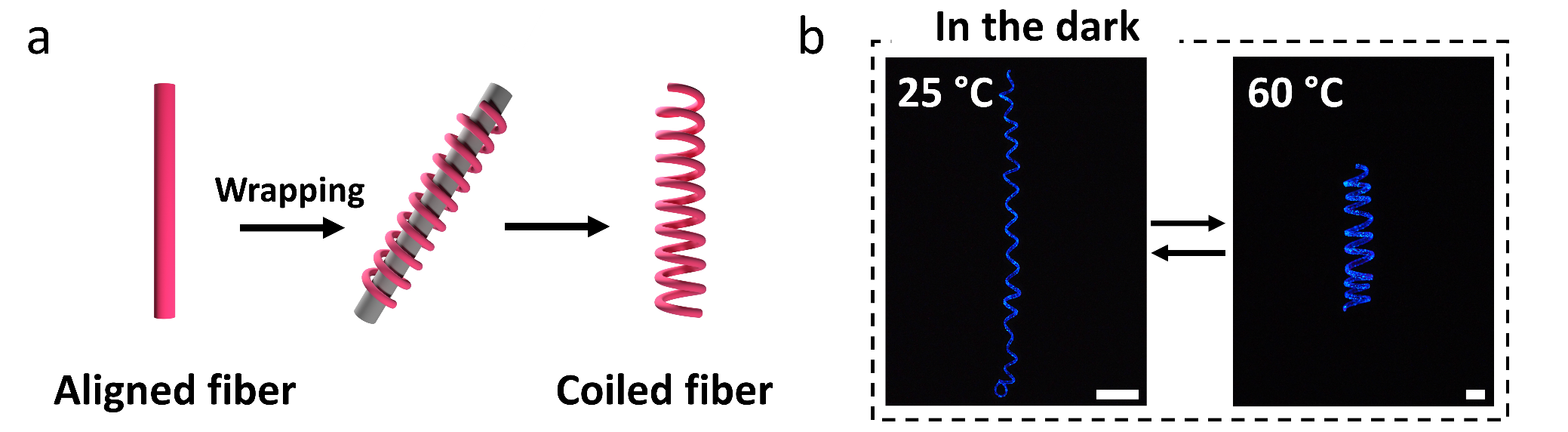
**

**Figure S46.** a) Schematic of the reprogramming of an aligned phosphorescent fiber into a coiled shape. b) Images showing the reprogrammed coiled fiber under thermal cycling between 25 °C and 60 °C, exhibiting fully reversible deformation while maintaining persistent phosphorescence (scale bars = 5 mm).

**
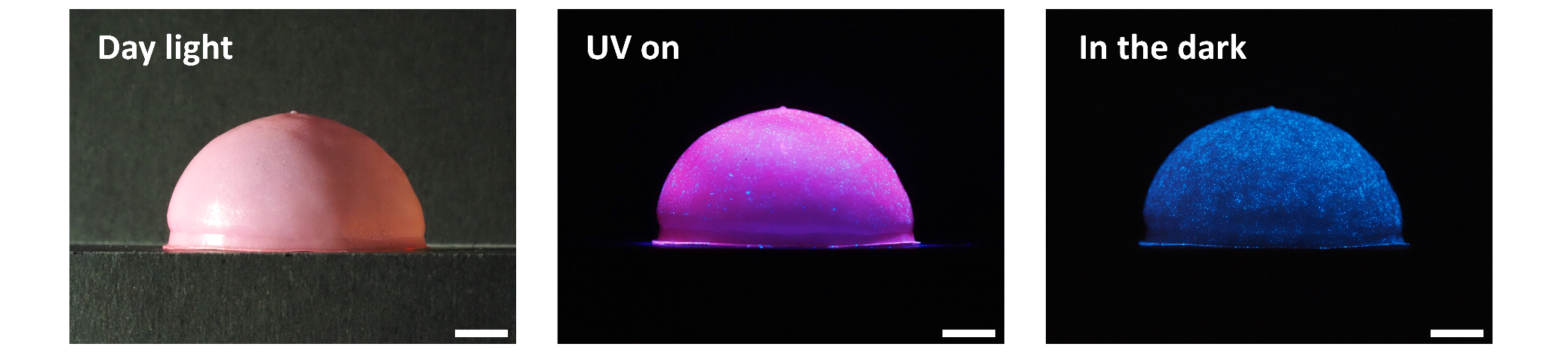
**

**Figure S47.** Photographs of a vacuum thermoformed glowing hemisphere under daylight, UV-on, and in the dark (scale bars = 10 mm).
